# Visible light-promoted CO_2_ fixation with imines to synthesize diaryl α-amino acids

**DOI:** 10.1038/s41467-018-07351-2

**Published:** 2018-11-22

**Authors:** Xinyuan Fan, Xu Gong, Mengyue Ma, Rui Wang, Patrick J. Walsh

**Affiliations:** 1 Institute of Advanced Synthesis, School of Chemistry and Molecular Engineering, Jiangsu National Synergetic Innovation Center for Advanced Materials, Nanjing Tech University, 30 South Puzhu Road, Nanjing, 211816 China; 2 Department of Chemistry, Roy and Diana Vagelos Laboratories, University of Pennsylvania, 231 South 34th Street, Philadelphia, PA 19104-6323 United States

## Abstract

Light-mediated transformations with CO_2_ have recently attracted great attention, with the focus on CO_2_ incorporation into C–C double and triple bonds, organohalides and amines. Herein is demonstrated visible light -mediated umpolung imine reactivity capable of engaging CO_2_ to afford α-amino acid derivatives. By employing benzophenone ketimine derivatives, CO_2_ fixation by hydrocarboxylation of C=N double bonds is achieved. Good to excellent yields of a broad range of α,α–disubstituted α-amino acid derivatives are obtained under mild conditions (rt, atmospheric pressure of CO_2_, visible light). A procedure that avoids tedious chromatographic purification and uses sustainable sunlight is developed to highlight the simplicity of this method.

## Abstract (web summary)

Fixation of CO_2_ in organic molecules is an area of great interest due to the implications in sustainable chemistry. Here, the authors show a visible light-mediated hydrocarboxylation of ketimines with atmospheric CO_2_ to afford a number of α,α–diaryl α-amino acid derivatives.

## Introduction

Sunlight is the most plentiful form of energy on the surface of the Earth, and therefore an attractive power source to drive chemical reactions. Indeed, photosynthesis is the prime example, wherein Nature converts carbon dioxide (CO_2_), an abundant and renewable feedstock, into organic molecules with many applications in nature, including storing energy. Chemists have long sought methods to emulate Nature’s ability to harness light for the conversion of CO_2_ into value-added organic compounds^1–7^. The challenge lies in carbon dioxide’s high kinetic and thermodynamic stability, typically requiring the use of very reactive reagents for direct incorporation of CO_2_ into organic molecules^8–13^. The emergence of photoredox catalysis, however, has enabled the incorporation of CO_2_ into organic compounds, fueling this budding research area^14–21^.

**Fig. 1:**
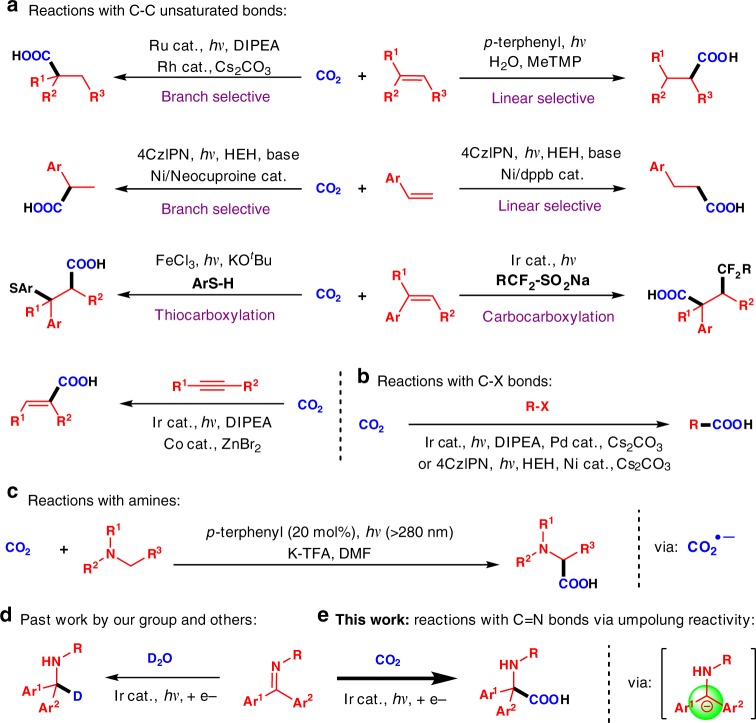
Fixation of CO_2_ under photoredox catalysis. **a** Reaction of CO_2_ with olefins and alkynes. **b** Reactions of C–X bonds with CO_2_. **c** C–H functionalization of amines with CO_2_. **d** Past work by our group and Polyzos. **e** Unpolung hydrocarboxylation of C=N bonds with CO_2_ (this work)

Since photoredox catalysis with CO_2_ often involves one electron reduction processes, olefins are the common radical acceptors in this chemistry. Photoredox catalysis mediated hydrocarboxylations of olefins have been demonstrated to yield linear^22^ and branched^23^ selective products by the Iwasawa and Jamison groups, respectively (Fig. 1a). Under dual photoredox/nickel catalysis ligand-controlled regioselective hydrocarboxylation of olefins was developed by König and coworkers (Fig. 1a)^24^. Difunctionalization of olefins using photoredox catalysis and CO_2_ have been realized by the Yu group^25^ (thiocarboxylation, Fig. 1a) and the Martín group^26^ (carbocarboxylation, Fig. 1a). The Zhao and Wu groups recently revealed that alkynes can undergo cobalt(II) catalyzed photoredox hydrocarboxylations to yield *cis*-α,β-unsaturated carboxylic acids (Fig. 1a)^27^. Aryl and alkyl halides were reported to react with CO_2_ using visible light photoredox in combination with transition metal catalysis by Iwasawa and Martin^28^ and by König^29^ and their coworkers (Fig. 1b).

A related CO_2_ research area with great potential is the photoredox-catalyzed fixation of CO_2_ to forge unnatural α-amino acids^30–35^. In pioneering work, Jamison and coworkers reported the coupling of tertiary amines with CO_2_ to afford α-amino acids mediated by >280 nm light using *p*-terphenyl (Fig. 1c, CO_2_^•–^)^36^. While our manuscript was under review, an example of photoredox catalytic hydrocarboxylation of enamides and imines using CO_2_ was reported by the Yu group, affording unnatural α-amino acids in good yields^37^.

Imines are known to participate in photoredox catalyzed processes, typically undergoing reduction followed by radical coupling reactions at the formerly carbonyl carbon^38–43^. In contrast to this expected reactivity pattern, we^44^, and Polyzos et al.^45^, have recently discovered that under visible light catalysis, benzophenone-based ketimines undergo one electron reductions to generate carbanions that abstract protons from water (or deuterium from D_2_O) via an umpolung reactivity (Fig. 1d)^46^. Based on this reaction, we hypothesized that the nucleophilicity of this intermediate might be sufficient to engage CO_2_ providing α-amino acids. Herein, we report a visible light and sunlight mediated photoredox hydrocarboxylation of imines and the synthesis of α,α-disubstituted α-amino acids (Fig. 1e).

## Results

### Reaction development and catalyst screening

At the outset of this work, we decided to limit ourselves to convenient conditions, so all reactions were conducted with atmospheric pressure of CO_2_ using a balloon at room temperature. Benzophenone imine **1a** was used as the standard substrate to test our abovementioned hypothesis. A mixture of **1a**, CO_2_ gas and Cy_2_NMe (*N,N*-dicyclohexylmethylamine) as sacrificial electron donor in acetonitrile was subjected to irradiation under blue LED in the presence of various photoredox catalysts (2 mol%). Unfortunately, the desired α-amino acid **2a** was not detected when the common photoredox catalysts [Ru(bpy)_3_](PF_6_)_2_ (bpy: 2,2′-bipyridine) or [Ru(bpz)_3_](PF_6_)_2_ (bpz: 2,2′-bipyrazine) were employed (**Ru-1** and **Ru-2**, Table 1, entries 1 and 2). *fac*-Ir(ppy)_3_ (**Ir-1**, ppy: 2-phenylpyridine) and its derivatives (**Ir-2** and **Ir-3**) were tested next, but **2a** was not observed after 24 h (entries 3–5). We then focused on [Ir(ppy)_2_(bpy)]PF_6_ type catalysts, as they generally showed higher catalytic activity in our previous study^44^. To our delight, the target product **2a** was observed with 29% assay yield (AY, determined after reaction workup by ^1^H nuclear magnetic resonance (NMR) integration) when [Ir(2′,4′-F_2_-ppy)_2_(4,4′-*t*Bu_2_-bpy)]PF_6_ (**Ir-4**, *t*Bu: tertiary butyl) was used (entry 6). Improved yields were observed with catalysts **Ir-5** or **Ir-6** (36 and 78% AY, entries 7 and 8, respectively). Finally, it was found that [Ir(ppy)_2_(4,4′-*t*Bu_2_-bpy)]PF_6_ (**Ir-7**) efficiently promoted the hydrocarboxylation of **1a** with CO_2_, affording the desired product **2a** in 92% AY (entry 9).

**Table 1:** Optimization of catalytic hydrocarboxylation of **1a** using CO_2_^a^

Entry	PC (2 mol%)	Amine	AY (%)^b^
1	**Ru-1**	Cy_2_NMe	<5
2	**Ru-2**	Cy_2_NMe	<5
3	**Ir-1**	Cy_2_NMe	<5
4	**Ir-2**	Cy_2_NMe	<5
5	**Ir-3**	Cy_2_NMe	<5
6	**Ir-4**	Cy_2_NMe	29
7	**Ir-5**	Cy_2_NMe	36
8	**Ir-6**	Cy_2_NMe	78
9	**Ir-7**	Cy_2_NMe	92
10^c^	**Ir-7**	Me_3_N	<5
11	**Ir-7**	Et_3_N	31
12	**Ir-7**	DIPEA	42
13	**Ir-7** (1 mol%)	Cy_2_NMe	91
14^d^	**Ir-7** (0.5 mol%)	Cy_2_NMe	92 (89)
15	**Ir-7** (0.1 mol%)	Cy_2_NMe	63
16^e^	None	Cy_2_NMe	<5
17^f^	**Ir-7** (no light)	Cy_2_NMe	<5

^a^Reactions conducted with **1a** (0.1 mmol), CO_2_ (balloon), catalyst (0.1–2 mol%) and amine (0.2 mmol) in 1 mL MeCN at room temperature under 20 W blue LED irradiation for 24 h

^b^Assay yields (AY) determined by ^1^H NMR integration using mesitylene as internal standard

^c^Reduction of **1a** was observed as the major product

^d^AY is 92%, isolated yield is 89%

^e^Reaction was conducted without catalyst

^f^Reaction was conducted in the dark

### Sacrificial electron donor study

Interestingly, when sacrificial electron donors trimethylamine (TMA), triethylamine (TEA), or *N,N*-diisopropylethylamine (DIPEA) were used, we noticed that the reaction solutions remained homogeneous over the course of the reactions. In contrast, with Cy_2_NMe the reaction mixture was initially homogeneous, but a precipitate was observed as the reaction time increased (see Supplementary Figure 1). ^1^H NMR analysis of reactions using TMA, TEA, or DIPEA (entries 10–12) showed that **1a** was completely consumed and the main product was the reduction product shown in Fig. 1d. This is possibly caused by the photocatalysis promoting the decarboxylation of the amino acid product, which has been previously reported in other systems^47–50^. The success of Cy_2_NMe is likely due to the precipitation of the product, which serves to protect it from decarboxylation. Indeed, when the product salt [Ph_2_C(CO_2_)NHBn•H_2_NCy_2_] was dissolved in DMF and then irradiated with blue LED in the presence of catalyst **Ir-7** (0.5 mol%) for 20 h, 85% of the amino acid product was decarboxylated. In contrast, only 30% decarboxylation was observed upon irradiation of a heterogeneous solution of Ph_2_C(CO_2_)NHBn•H_2_NCy_2_ with MeCN and **Ir-7** in the absence of CO_2_.

### Catalyst loading study

Excellent AY was observed with catalyst loading as low as 1 mol% (91% AY, entry 13) or even 0.5 mol% (92% AY and 89% isolated yield, entry 14). Further reducing the loading to 0.1 mol%, however, furnished only 63% AY (entry 15). No desired product was detected in the absence of either catalyst **Ir-7** or light (entries 16–17).

### Substrate scope evaluation

**Fig. 2:**
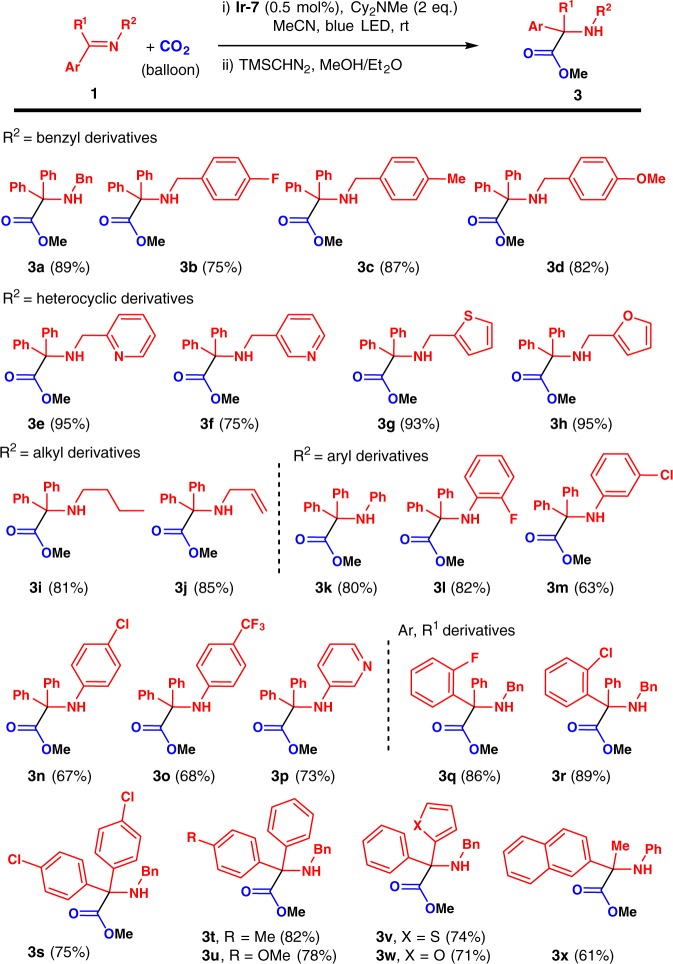
Visible light-mediated catalytic hydrocarboxylation of ketimines using CO_2_. Reactions were conducted with **1** (0.2 mmol), CO_2_ (balloon), **Ir-7** (0.5 mol%), and Cy_2_NMe (0.4 mmol) in 2 mL MeCN at RT under 20 W blue LED irradiation. 2 mol% of **Ir-7** was used in the case of **3x.** Isolated yields

**Fig. 3:**
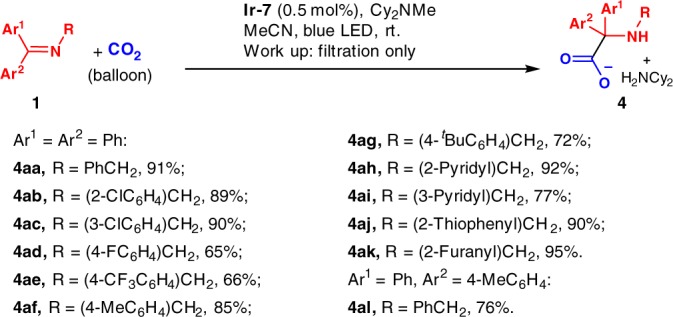
Chromatography-free syntheses. Preparation of α-amino acids using CO_2_ and ketimines without chromatographic purification

With the optimized conditions in hand (Table 1, entry 14), we next examined the substrate scope. To avoid the problematic purification of the highly polar α-amino acid products, their carboxyl groups were transformed into methyl esters **3** by treating the crude products **2** with TMSCHN_2_. In the event, the methyl ester of **2a** was isolated without loss of product yield (Figs. 2 and 3a, 89% yield). Substrates with benzyl groups bearing electron-withdrawing 4-F, or electron-donating 4-Me or 4-OMe groups were smoothly hydrocarboxylated with CO_2_ and esterified, affording the products in good to excellent yields (**3b–3d**, 75–87% yield).

Substitution of the *N-*benzyl’s phenyl group for 2-pyridyl gave **3e** in 95% yield and the 3-pyridyl analogue (**3f**) was obtained in 75% yield. Excellent yields were observed for the 2-thiophenyl (**3g**, 93%) and 2-furanyl substrates (**3h**, 95%). Alkyl ketimines were tested next, with 81% yield obtained for the *n*-butyl ketimine (**3i**). Interestingly, use of *N*-allyl ketimine **1j** provided the *N*-allyl α-amino ester (**3j**) in 85% yield, indicating that the olefin group is tolerated under the reaction conditions. *N*-Aryl substrates were also examined. The *N*-Ph substrate reacted to provide the desired product (**3k**) in 80% yield. Introduction of substituents into the *N*-phenyl group, e.g., 2-F, 3-Cl, 4-Cl, or 4-CF_3_, did not dramatically affect their reactivity (**3l–3o**, 63–82% yields). Reaction with the heterocyclic substrate, *N*-3-pyridyl ketimine, afforded the hydrocarboxylated product **3p** in 73% yield. In cases where yields were lower, byproducts derived from imine reduction were observed^44,45^.

Substrates derived from various benzophenone derivatives were next examined. Thus, benzophenone imines containing a 2-F or 2-Cl substituted aryl reacted smoothly with CO_2_ to give the α-amino esters **3q** and **3r** in 86% and 89% yield, respectively. The 4,4′-dichloro derivative **1s** afforded the amino ester **3s** in 75% yield. The electron-donating 4-Me and 4-OMe groups on the benzophenone imines did not affect their reactivity. The desired products were obtained in 82% for **3t** and 78% for **3u**. Heterocyclic substrates were also tolerated. The 2-thiophenyl and 2-furanyl substrates reacted with CO_2_ to yield the hydrocarboxylation products **3v** and **3w** in 74% and 71% yield, respectively. To our delight, the alkyl ketimine derived from 2-naphthylmethyl ketone reacted with CO_2_ to afford **3x** in 61% yield (catalyst loading 2 mol%). However, attempts to perform the hydrocarboxylation of *N*-phenyl dimethylketimine failed, yielding a complex mixture with no observation of the desired product.

### Nonchromatographic purification

Purification using chromatographic methods is often challenging and costly, particularly on larger scales. As noted above, hydrocarboxylation reactions led to the formation of precipitates. Thus, in the case of **1a**, upon reaction completion, the precipitate was easily isolated by filtration. Analysis using NMR and mass spectroscopy led to the assignment of the precipitate as the Cy_2_NH_2_^+^ salt of α-amino carboxylate (**4aa**, Fig. 3). Isolation of the precipitate in this fashion provided **4aa** in 91% yield with high purity. Additional substrates were tested to examine the generality of this method. Good to excellent yields with high purities were obtained in all cases simply by filtration of the reaction mixtures (65–95% yields, **4ab**–**4al**, Fig. 3).

**Fig. 4:**
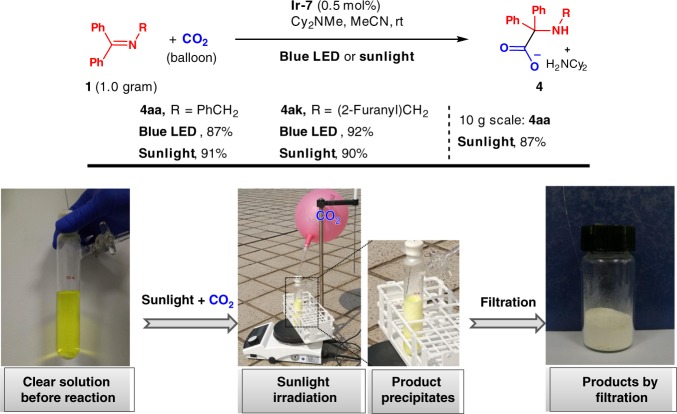
Upscaling experiments. Gram-scale preparation of α-amino acids via fixation of CO_2_ promoted by visible light or sunlight

The two key advantages of this method are: (1) atmospheric pressure of CO_2_ gas is used so that special equipment, such as autoclaves, is not required, and (2) chromatographic purification can be avoided, enabling large scale reactions to be easily conducted. Thus, gram scale reactions with two substrates were performed. Upon reaction completion, the hydrocarboxylation products were isolated by filtration in good yields (87% for **4aa**, 92% for **4ak**, Fig. 4) (also see Supplementary Figure 2).

### Sunlight powered reactions

We next desired to test the use of sunlight to drive our photocatalytic amino acid synthesis. Thus, 1g scale reactions of these substrates were performed outdoors with sunlight instead of blue LED. The reactions were complete in 10 h, and the resulting amino acids were obtained by filtration in high yields (91% for **4aa**, 90% for **4ak**, Fig. 4) (see Supplementary Figure 3). Given the straightforward and practical nature of this method, we were curious if it could be further scaled. Thus reaction with 10 g of **1a** was conducted with outdoor sunlight. The reaction was complete in 18 h, affording the product in 87% yield (Fig. 4, Supplementary Figure 4). The success of these experiments indicates the great practicality of fixation of the greenhouse gas CO_2_ by harvesting sustainable sunlight energy, affording fine chemicals.

### Hydrocarboxylation of enantioenriched amino acid derivatives

**Fig. 5:**
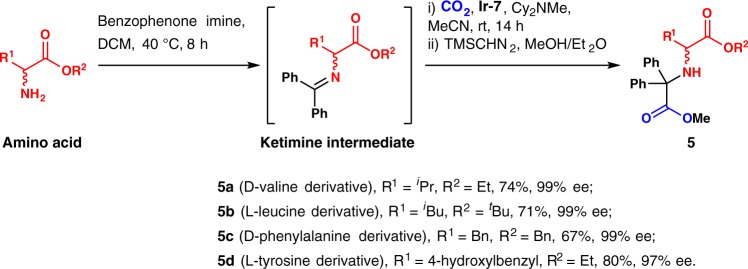
Further synthetic applications. Synthesis of enantioenriched iminodiacetic acid derivatives from α-amino acid derivatives

The diarylacetic acid group can be easily anchored to amines using our method. For instance, hydrocarboxylation of the diphenylketimine of d-valine ethyl ester under standard conditions led to the d-valine iminodiacetic acid derivative in 74% overall yield (**5a**, Fig. 5). Note that the enantiomeric excess (ee) was maintained (99%). Likewise, L-leucine, d-phenylalanine, and l-tyrosine iminodiacetic acid derivatives were obtained in good to high yield with high ee (**5b**–**5d**, 67–80% yield, >97% see, Supplementary Figures 47–50). Iminodiacetic acid derivatives have been broadly used as tridentate chelating ligands for metals^51^.

### Utility of the products

**Fig. 6:**
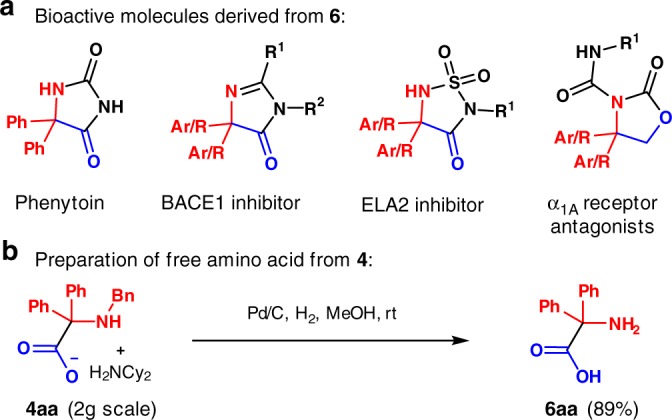
Applications of α,α-diaryl α-amino acids. **a** Potential targets and **b** deprotection of amino acid derivative **4aa** on 2 gram scale

α,α-Diaryl α-amino acids have recently been shown to be excellent amino sources in transamination reactions for the synthesis of bioactive nitrogen-containing compounds^52^. α,α-Diaryl α-amino acids can also be used to prepare phenytoin and its derivatives (Fig. 6a), which is a clinical anticonvulsant drug (Dilantin^®^) on the WHO’s list of essential medicines^53^. Many other biologically active compounds are synthesized using α,α-disubstituted α-amino acids. For instance, these products are used to synthesize imidazol-4-one type molecules, which are BACE1 inhibitors for treating Alzheimer’s disease^54^. Other bioactive molecules, including ELA2 inhibitors for treating obesity^55,56^, and α_1_A receptor antagonists^57^, are also synthesized from α,α-disubstituted α-amino acids (Fig. 6a). The free amino acid **6aa**, which would be used to make the abovementioned compounds, was easily obtained by debenzylation employing hydrogen and catalytic palladium on carbon in 89% yield after filtration and precipitation (Fig. 6b). It is noteworthy that both the hydrocarboxylation and deprotection steps were performed without chromatography, highlighting the practicality of this method for large scale applications.

### Mechanistic studies

**Fig. 7:**
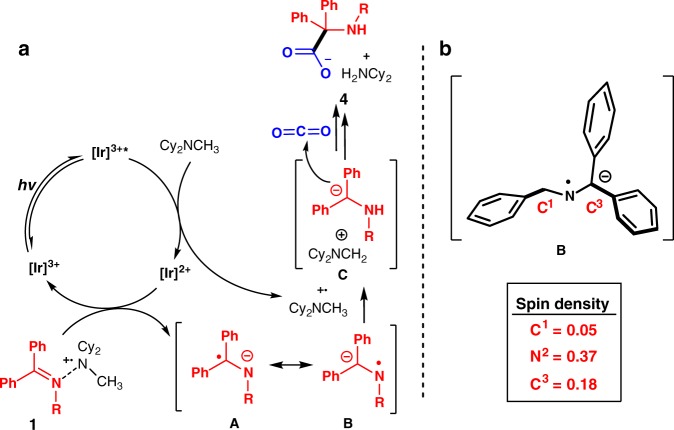
Reaction pathway. **a** Plausible mechanism. **b** Computational spin density of the radical anion intermediate

We next conducted preliminary investigations to probe the mechanism. UV–vis spectra indicated that only [Ir]^3+^ catalyst is capable of absorbing visible light, so the reaction is likely initiated by irradiation of [Ir]^3+^ by light to give its excited state [Ir]^3+^* (see Supplementary Figure 51). Stern–Volmer fluorescence quenching experiments indicate that Cy_2_NMe acts as electron donor and reduces [Ir]^3+^* to [Ir]^2+^ and generating the radical cation [Cy_2_NMe]^+^ (see Supplementary Figure 52). On the basis of previous reports, the resulting amine radical cation coordinates with imines to form a 2-center-3-electron bond^58–60^, facilitating reduction by [Ir]^2+^ to form the radical anion intermediate **A/B** and regenerating [Ir]^3+^^60,61^. Due to the high reactivity of the *N-*radical in resonance form **B**, it is quickly quenched by the amine radical cation via HAT, to give the α-amino carbanion intermediate **C**. **C** acts as a strong nucleophile and attacks CO_2_ to give the product. The iminium ion [Cy_2_N = CH_2_]^+^ reacts with advantageous water to generate Cy_2_NH, which forms insoluble salts **4** with α-amino acid (Fig. 7a). The key factor controlling reactivity in this system is the significant contribution of resonance form **B**. We previously presented computational evidence that the radical anion intermediate has greater spin density on nitrogen (0.37) than on the carbon labeled C3 (0.18) in Fig. 7, indicating that the C3 carbon carries more anion character (Fig. 7b)^62^. In addition, previous experimental results on electrochemical reduction of ketimines by Reed et al.^63^, as well as our photochemical reduction of ketimines also indicated that C3 carbon carries more anionic character^44^.

## Discussion

Carboxylation reactions employing CO_2_ have attracted considerable attention, because CO_2_ is an abundant, renewable, low cost, and nontoxic C1 source. Recent advances include photoredox catalyzed additions of CO_2_ to olefins, alkynes, aryl halides, alkyl halides and C–H functionalization of amines to afford a variety of useful carboxylic acids (Fig. 1). The conceptual advance of this work is that stabilizing groups at the carbonyl carbon can invert the reactivity of the ketiminyl radical anion, enabling nucleophilic addition to CO_2_. Such additions are usually observed with reactive organometallic reagents possessing Lewis acidic metal centers capable of activating CO_2_ toward addition (i.e., Grignard and organolithium reagents).

Our efficient photoredox catalyzed reaction of imines with CO_2_ produces unnatural α-amino acids under mild conditions (rt, atmospheric pressure of CO_2_, visible light, 0.5 mol% air-stable commercial catalyst) accessible in the vast majority of laboratories world-wide. The mildness of these conditions allows the direct use of the sunlight to promote the fixation of CO_2_ gas. Additionally, a straightforward procedure avoiding chromatographic purification of the α-amino acid products has been developed that involves filtration of the reaction mixture. The simplicity of this method is demonstrated by conducting the hydrocarboxylation on scale using both LED and outdoor sunlight. The practicality of this method was further evaluated by synthesizing the α,α-diphenylglycine (**6aa**, a commercial compound that is frequently used as a pharmaceutical drug precursor) on scale using procedures that avoid chromatographic purification.

Based on our interests in protein/peptide modifications using unnatural amino acids^32,33^, the α,α-disubstituted α-amino acids prepared herein are viewed as excellent candidates for further study. In particular, modification of GLP1, which is a peptide drug for the treatment of type 2 diabetes is currently under investigation by genetic code expansion techniques^30,33^ and chemical synthesis in our laboratories (see Supplementary Methods). It has been reported that the replacement of Ala2 of GLP1 with an α,α-disubstituted α-amino acid improves its stability against enzymatic degradation^64^. The straightforward nature of our method for the synthesis of these unnatural amino acids is facilitating investigations onto protein/peptide modifications, which will be reported in due course.

## Methods

### Typical procedure for the gram-scale synthesis of **4** using visible light

Ketimine **1a** (1.0 g, 3.7 mmol), catalyst **Ir-7** (16.6 mg, 0.0185 mmol, 0.5 mol%), Cy_2_NMe (1.58 mL, 7.4 mmol), MeCN (37 mL), and a magnetic stirring bar were charged into an oven-dried 50 mL Schlenk tube under nitrogen. The tube was sealed with a septum. CO_2_ gas in a balloon was bubbled into the mixture under stirring for 2 min through a needle, which was then lifted up out of the solution and was kept in the tube. The mixture was placed under a 20 W blue LED light source and stirred at ambient temperature (15–20 °C). A white precipitate appeared as the reaction proceeded. Upon completion of the reaction as monitored by thin-layer chromatography, the tube was opened and cooled down in an ice bath. The precipitate was collected by filtration, and washed using cold MeCN (3 × 4 mL). The desired compound **4aa** was obtained after drying under reduced pressure (1.6 g, 87%, see Supplementary Figure 2).

## Electronic supplementary material

Supplementary Information

## Data Availability

The authors declare that the data supporting the findings of this study are available within the article and its Supplementary Information files.
