# Supplementary material for: Visible light-promoted CO2 fixation with imines to synthesize diaryl α-amino acids
Source: Nat Commun. 2018 Nov 22;9:4936. doi: 10.1038/s41467-018-07351-2 (PMC6250672; doi:10.1038/s41467-018-07351-2)
Supplement: Supplementary file 1 — Supplementary Information [file 41467_2018_7351_MOESM1_ESM.pdf]

Supplementary Information

Visible Light-Promoted CO_2 Fixation with Imines to Synthesize Diaryl α-Amino Acids

Fan et al.

Supplementary Information

Visible Light-Promoted CO_2 Fixation with Imines to Synthesize Diaryl α-Amino Acids

Contents

Supplementary Methods......s2

General Considerations......s2

Typical Experimental Procedure to Synthesize **3**......s2

Typical Experimental Procedure to Prepare **4**......s11

Gram-Scale Experiments using Visible Light......s16

Gram-Scale Experiments using Sunlight......s17

Procedure to Prepare Enantioenriched Iminodiacetic Acid from α-Amino Acid Derivatives......s17

Debenzylation Procedure to Synthesize Free Amino Acid......s19

UV-visible Absorption Analysis......s20

Stern-Volmer Luminescence Quenching Analysis......s20

Chemical Synthesis of Unnatural GLP1 Fragment......s20

Supplementary Figures......s22

Supplementary Figures of Reactions......s22

Supplementary Figures of ^{1}H, ^{19}F and $^{13}C\{^{1}H\}$ NMR Spectra......s24

Supplementary Figures of HPLC Spectra......s70

Supplementary Figures of Mechanistic Studies......s73

Supplementary References......s73

Supplementary Methods

General Considerations. All chemicals were purchased from J&K Scientific or Energy Chemical unless otherwise specified. All reactions were conducted under a nitrogen atmosphere with oven-dried glassware by using standard Schlenk or vacuum line techniques. All solutions were handled under nitrogen and transferred via syringe. Anhydrous solvents were purchased from Sigma-Aldrich and directly used. Unless otherwise stated, reagents were commercially available and used as purchased. The progress of the reactions was monitored by thin-layer chromatography using TLC plates purchased from commercial suppliers and visualized by short-wave ultraviolet light or by treatment with ninhydrin. Flash chromatography was performed with silica gel (200–300 mesh) or basic aluminum oxide (100–200 mesh). The infrared spectra were obtained with KBr plates by using an IS10 FT-IR Spectrometer (ThermoFisher Corporation). High resolution mass spectrometry (HRMS) data were obtained on a Waters LC-TOF mass spectrometer (Xevo G2-XS QTof) using electrospray ionization (ESI) in positive or negative mode. Melting points were measured using a SGW X-4 Melt-Temp apparatus and were uncorrected. NMR spectra were recorded on a Brüker 400 MHz Fourier transform spectrometer at Nanjing Tech University NMR facility. Chemical shifts in ^{1}H spectra were referenced to TMS, and in ^{13}C{^{1}H} NMR spectra were referenced to residual solvent. All coupling constants are reported in hertz.

Typical Experimental Procedure to Synthesize 3

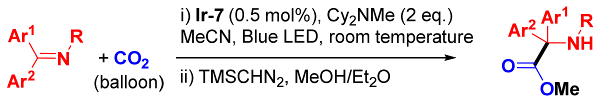

Ketimine **1** (0.2 mmol), catalyst **Ir-7** (0.9 mg, 0.5 mol %), Cy_2NMe (85.6 µL, 0.4 mmol), MeCN (2 mL) and a magnetic stirring bar were charged into an oven-dried 5 mL vial under nitrogen. The vial was sealed with a septum (Note that ahydrous condition is important in order to avoid the imine reduction reactions with water). CO_2 gas in a balloon was bubbled into the mixture under stirring for 30 seconds through a needle, which was then lifted up out of the solution and was kept in the vial. The mixture was placed under a 20 W blue LED light source and stirred at ambient temperature (15—20 ^{o}C). Upon completion of the reaction as monitored by TLC, the reaction vessel was opened to air and the volatile materials were removed using a rotary evaporator under reduced pressure. The crude residue was dissolved in 2 mL MeOH/Et_2O (1/2), $TMSCHN_2$ (0.3 mL, 0.6 mmol, 2 M in hexanes) was added dropwisely. The mixture was stirred at ambient temperature until the completion of the methylation reaction. All the volatile materials were removed using a rotary evaporator under reduced pressure, and the product was purified by flash chromatography on silica gel using ethyl acetate and petroleum ether as eluents.

Methyl 2-(benzylamino)-2,2-diphenylacetate (3a).

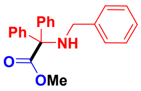

The reaction was performed following the Typical Experimental Procedure with N-benzyl-1,1-diphenylmethanimine (54.4 mg, 0.2 mmol), catalyst **Ir-7** (0.9 mg, 0.5 mol %), Cy_2NMe (85.6 µL, 0.4 mmol), and CO_2 (in balloon) in 2 mL MeCN. Upon completion, the amino acid product was converted into its methyl ester. The crude product was purified by chromatography on silica gel (eluted with petroleum ether/EtOAc = 50/1) to give the desired product (60.0 mg, 89% yield) as white solid.

m.p. 73.1-73.3 °C. R_f = 0.40 (hexanes : ethyl acetate = 20:1). ^{1}H NMR (400 MHz, $CDCl_3$): δ 7.53 (dd, J = 7.4, 1.5 Hz, 4H), 7.40 – 7.21 (m, 11H), 3.76 (s, 3H), 3.45 (s, 2H), 2.54 (s, 1H) ppm; $^{13}C\{^1H\}$ NMR (100 MHz, $CDCl_3$): δ 174.16, 141.56, 140.44, 128.49, 128.37, 128.22, 128.15, 127.53, 127.08, 72.98, 52.76, 48.41 ppm. IR (thin film): 3326, 1716, 1232, 1183, 991, 742, 725, 701 cm^{-1}; HRMS calcd for $C_{22}H_{22}NO_2^+$ 332.1651, observed 332.1658 $[M+H]^+$.

Methyl 2-((4-fluorobenzyl)amino)-2,2-diphenylacetate (**3b**).

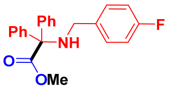

The reaction was performed following the Typical Experimental Procedure with N-(4-fluorobenzyl)-1,1-diphenylmethanimine (57.9 mg, 0.2 mmol), catalyst **Ir-7** (0.9 mg, 0.5 mol %), Cy_2NMe (85.6 µL, 0.4 mmol), and CO_2 (in balloon) in 2 mL MeCN. Upon completion, the amino acid product was converted into its methyl ester. The crude product was purified by chromatography on silica gel (eluted with petroleum ether/EtOAc = 50/1) to give the desired product (52.4 mg, 75% yield) as white solid.

m.p. 82.7-83.4 °C. R_f = 0.39 (hexanes : ethyl acetate = 20 : 1). ^{1}H NMR (400 MHz, $CDCl_3$): δ 7.50 (d, J = 7.4 Hz, 4H), 7.31 (dt, J = 14.5, 6.9 Hz, 8H), 6.99 (t, J = 8.7 Hz, 2H), 3.77 (s, 3H), 3.40 (s, 2H), 2.54 (s, 1H) ppm; $^{13}C\{^1H\}$ NMR (100 MHz, $CDCl_3$): δ 173.82, 161.73 (d, J = 244.4 Hz), 141.16, 135.89, 129.45 (d, J = 7.8 Hz), 128.11, 127.86, 127.30, 114.92 (d, J = 21.2 Hz), 72.71, 52.48, 47.43 ppm. IR (thin film): 1719, 1506, 1232, 1218, 1208, 1180, 755, 701 cm^{-1}; HRMS calcd for $C_{22}H_{21}FNO_2^+$ 350.1556, observed 350.1558 $[M+H]^+$.

Methyl 2-((4-methylbenzyl)amino)-2,2-diphenylacetate (**3c**).

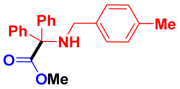

The reaction was performed following the Typical Experimental Procedure with N-(4-methylbenzyl)-1,1-diphenylmethanimine (57.1 mg, 0.2 mmol), catalyst **Ir-7** (0.9 mg, 0.5 mol %), Cy_2NMe (85.6 µL, 0.4 mmol), and CO_2 (in balloon) in 2 mL MeCN. Upon completion, the amino acid product was converted into its methyl ester. The crude product was purified by chromatography on silica gel (eluted with petroleum ether/EtOAc = 50/1) to give the desired product (60.1 mg,87% yield) as colorless oil.

R_f = 0.37 (hexanes : ethyl acetate = 20 : 1). ^{1}H NMR (400 MHz, $CDCl_3$): δ 7.53 (d, J = 8.0 Hz, 4H), 7.33 (t, J = 7.6 Hz, 4H), 7.29 – 7.23 (m, 4H), 7.13 (d, J = 7.8 Hz, 2H), 3.77 (s, 3H), 3.40 (s, 2H), 2.49 (s, 1H), 2.33 (s, 3H) ppm; $^{13}C\{^1H\}$ NMR (100 MHz, $CDCl_3$): δ 174.17, 141.65, 137.41, 136.67, 129.16, 128.35, 128.17, 128.12, 127.48, 73.00, 52.72, 48.16, 21.22 ppm. IR (thin film): 3435, 3054, 1595, 1422, 1265, 1176, 739, 705 cm^{-1}; HRMS calcd for $C_{23}H_{24}NO_2^+$ 346.1807, observed 346.1811 $[M+H]^+$.

Methyl 2-((4-methoxylbenzyl)amino)-2,2-diphenylacetate (**3d**).

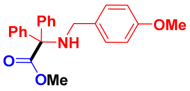

The reaction was performed following the Typical Experimental Procedure with N-(4-methoxybenzyl)-1,1-diphenylmethanimine (60.3 mg, 0.2 mmol), catalyst **Ir-7** (0.9 mg, 0.5 mol %), Cy_2NMe (85.6 µL, 0.4 mmol), and CO_2 (in balloon) in 2 mL MeCN. Upon completion, the amino acid product was converted into its methyl ester. The crude product was purified by chromatography on silica gel (eluted with petroleum ether/EtOAc = 30/1) to give the desired product (57.4 mg, 82% yield) as colorless oil.

R_f = 0.26 (hexanes : ethyl acetate = 20 : 1). ^{1}H NMR (400 MHz, $CDCl_3$): δ 7.53 (d, *J* = 8.3 Hz, 4H), 7.34 (t, *J* = 7.2 Hz, 4H), 7.31 – 7.24 (m, 4H), 6.87 (d, *J* = 8.7 Hz, 2H), 3.81 (s, 3H), 3.78 (s, 3H), 3.39 (s, 2H), 2.50 (s, 1H) ppm; ^{13}C{^{1}H} NMR (100 MHz, $CDCl_3$): δ 174.20, 158.74, 141.63, 132.57, 129.42, 128.38, 128.14, 127.51, 113.86, 73.00, 55.41, 52.77, 47.85 ppm. IR (thin film): 3054, 1732, 1513, 1447, 1421, 1261, 1174, 737 cm^{-1}; HRMS calcd for $C_{23}H_{24}NO_3^+$ 362.1756, observed 362.1752 $[M+H]^+$.

Methyl 2,2-diphenyl-2-((pyridin-2-ylmethyl)amino)acetate (3e).

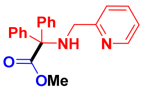

The reaction was performed following the Typical Experimental Procedure with 1,1-diphenyl-N-(pyridin-2-ylmethyl)methanimine (54.5 mg, 0.2 mmol), catalyst **Ir-7** (0.9 mg, 0.5 mol %), Cy_2NMe (85.6 µL, 0.4 mmol), and CO_2 (in balloon) in 2 mL MeCN. Upon completion, the amino acid product was converted into its methyl ester. The crude product was purified by chromatography on silica gel (eluted with petroleum ether/EtOAc = 3/1) to give the desired product (63.2mg, 95% yield) as yellow oil.

R_f = 0.75 (hexanes : ethyl acetate = 1 : 1). ^{1}H NMR (400 MHz, $CDCl_3$): δ 8.52 (d, *J* = 5.5 Hz, 1H), 7.60 (td, *J* = 7.7, 1.8 Hz, 1H), 7.51 (dd, *J* = 8.3, 1.2 Hz, 4H), 7.37 – 7.24 (m, 7H), 7.16 – 7.09 (m, 1H), 3.74 (s, 3H), 3.62 (s, 2H), 3.34 (s, 1H) ppm; ^{13}C{^{1}H} NMR (100 MHz, $CDCl_3$): 174.07, 159.64, 149.16, 141.41, 136.46, 128.60, 128.14, 127.57, 122.26, 121.93, 73.02, 52.78, 49.69 ppm. IR (thin film): 3435, 3054, 1732, 1593, 1434, 1265, 738, 704 cm^{-1}; HRMS calcd for $C_{21}H_{21}N_2O_2^+$ 333.1603 observed 333.1601$[M+H]^+$.

Methyl 2,2-diphenyl-2-((pyridin-3-ylmethyl)amino)acetate (3f).

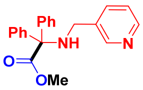

The reaction was performed following the Typical Experimental Procedure with 1,1-diphenyl-N-(pyridin-3-ylmethyl)methanimine (54.5 mg, 0.2 mmol), catalyst **Ir-7** (0.9 mg, 0.5 mol %), Cy_2NMe (85.6 µL, 0.4 mmol), and CO_2 (in balloon) in 2 mL MeCN. Upon completion, the amino acid product was converted into its methyl ester. The crude product was purified by chromatography on silica gel (eluted with petroleum ether/EtOAc = 3/1) to give the desired product (50.0 mg, 75% yield) as white solid.

m.p 89.0-89.1 °C R_f = 0.58 (hexanes : ethyl acetate = 1 : 1). ^{1}H NMR (400 MHz, $CDCl_3$): δ 8.53 (d, *J* = 2.2 Hz, 1H), 8.49 (dd, *J* = 4.8, 1.4 Hz, 1H), 7.75 – 7.67 (m, 1H), 7.50 (dd, *J* = 8.3, 1.2 Hz, 4H), 7.39 – 7.27 (m, 6H), 7.24 (dd, *J* = 7.6, 4.8 Hz, 1H), 3.78 (s, 3H), 3.46 (d, *J* = 5.0 Hz, 2H), 2.60 (s, 1H) ppm; ^{13}C{^{1}H} NMR (100 MHz, $CDCl_3$): δ 174.04, 149.82, 148.60, 141.21,

135.93, 128.47, 128.25, 127.75 (x2), 123.45, 73.06, 52.90, 45.92 ppm. IR (thin film): 1733, 1459, 1203, 1009, 755, 722, 704, 694 cm^{-1}; HRMS calcd for $C_{21}H_{21}N_2O_2^+$ 333.1603, observed 333.1639 $[M+H]^+$.

Methyl 2,2-diphenyl-2-((thiophen-2-ylmethyl)amino)acetate (**3g**).

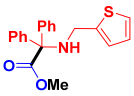

The reaction was performed following the Typical Experimental Procedure with 1,1-diphenyl-N-(thiophen-2-ylmethyl)methanimine (55.5 mg, 0.2 mmol), catalyst **Ir-7** (0.9 mg, 0.5 mol %), Cy_2NMe (85.6 µL, 0.4 mmol), and CO_2 (in balloon) in 2 mL MeCN. Upon completion, the amino acid product was converted into its methyl ester. The crude product was purified by chromatography on silica gel (eluted with petroleum ether/EtOAc = 30/1) to give the desired product (67.5 mg, 93% yield) as white solid.

m.p 88.2-88.4 °C R_f = 0.28 (hexanes : ethyl acetate = 20 : 1). ^1H NMR (400 MHz, $CDCl_3$): δ 7.52 (d, J = 7.9 Hz, 4H), 7.40 – 7.24 (m, 6H), 7.20 (ddd, J = 4.9, 2.4, 1.2 Hz, 1H), 6.93 (dt, J = 5.1, 2.9 Hz, 1H), 6.89 (s, 1H), 3.77 (s, 3H), 3.63 (s, 2H), 2.74 (s, 1H) ppm; $^{13}C\{^1H\}$ NMR (100 MHz, $CDCl_3$): δ 173.90, 144.18, 141.24, 128.32, 128.22, 127.63, 126.70, 124.46, 124.40, 72.83, 52.84, 43.52 ppm. IR (thin film): 3347, 1720, 1479, 1232, 1179, 988, 758, 699 cm^{-1}; HRMS calcd for $C_{20}H_{20}NO_2S^+$ 338.1215, observed 338.1219 $[M+H]^+$.

Methyl 2-((furan-2-ylmethyl)amino)-2,2-diphenylacetate (**3h**).

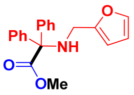

The reaction was performed following the Typical Experimental Procedure with N-(furan-2-ylmethyl)-1,1-diphenylmethanimine (52.3mg, 0.2 mmol), catalyst **Ir-7** (0.9 mg, 0.5 mol %), Cy_2NMe (85.6 µL, 0.4 mmol), and CO_2 (in balloon) in 2 mL MeCN. Upon completion, the amino acid product was converted into its methyl ester. The crude product was purified by chromatography on silica gel (eluted with petroleum ether/EtOAc = 30/1) to give the desired product (61.1 mg, 95% yield) as white solid.

m.p 82.8-83.1 °C R_f = 0.49 (hexanes : ethyl acetate = 10 : 1). ^1H NMR (400 MHz, $CDCl_3$): δ 7.50 (d, J = 7.8 Hz, 4H), 7.41 – 7.24 (m, 7H), 6.29 (s, 1H), 6.17 (s, 1H), 3.75 (s, 3H), 3.44 (s, 2H), 2.73 (s, 1H) ppm; $^{13}C\{^1H\}$ NMR (100 MHz, $CDCl_3$): δ 173.94, 153.86, 141.84, 141.11, 128.50, 128.19, 127.64, 110.28, 106.61, 72.74, 52.83, 41.49 ppm. IR (thin film): 1715, 1236, 1183, 1148, 741, 725, 701, 599 cm^{-1}; HRMS calcd for $C_{20}H_{20}NO_3^+$ 322.1443, observed 322.1437 $[M+H]^+$.

Methyl 2-(butylamino)-2,2-diphenylacetate (**3i**).

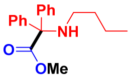

The reaction was performed following the Typical Experimental Procedure with N-butyl-1,1-diphenylmethanimine (47.5 mg, 0.2 mmol), catalyst **Ir-7** (0.9 mg, 0.5 mol %), Cy_2NMe (85.6 µL, 0.4 mmol), and CO_2 (in balloon) in 2 mL MeCN. Upon completion, the amino acid product was converted into its methyl ester. The crude product was purified by chromatography on silica gel (eluted with petroleum ether/EtOAc = 30/1) to give the desired product (48.2 mg, 81% yield) as colorless oil.

R$_f$ = 0.32 (hexanes : ethyl acetate = 20 : 1). ^{1}H NMR (400 MHz, CDCl$_3$): δ 7.45 (d, J = 7.2 Hz, 4H), 7.33 – 7.22 (m, 6H), 3.74 (s, 3H), 2.24 (t, J = 7.1 Hz, 2H), 2.05 (s, 1H), 1.48 (dt, J = 14.7, 7.0 Hz, 2H), 1.32 (dt, J = 14.4, 7.4 Hz, 2H), 0.86 (t, J = 7.3 Hz, 3H) ppm; ^{13}C{^{1}H} NMR (100 MHz, CDCl$_3$): δ 174.41, 141.85, 128.37, 128.01, 127.35, 73.02, 52.64, 43.79, 32.86, 20.55, 14.13 ppm. IR (thin film): 3054, 2958, 2930, 1732, 1265, 1197, 739, 705 cm^{-1}; HRMS calcd for $C_{19}H_{24}NO_2^+$ 298.1807, observed 298.18010 [M+H]$^+$.

Methyl 2-(allylamino)-2,2-diphenylacetate (3j).

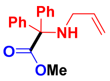

The reaction was performed following the Typical Experimental Procedure with N-allyl-1,1-diphenylmethanimine (44.3 mg, 0.2 mmol), catalyst **Ir-7** (0.9 mg, 0.5 mol %), Cy$_2$NMe (85.6 µL, 0.4 mol), and CO_2 (in balloon) in 2 mL MeCN. Upon completion, the amino acid product was converted into its methyl ester. The crude product was purified by chromatography on silica gel (eluted with petroleum ether/EtOAc = 30/1) to give the desired product (47.8 mg, 85% yield) as colorless oil.

R$_f$ = 0.33 (hexanes : ethyl acetate = 20 : 1). ^{1}H NMR (400 MHz, CDCl$_3$): δ 7.45 (d, J = 7.8 Hz, 4H), 7.33 – 7.24 (m, 6H), 6.10 – 5.77 (m, 1H), 5.21 (d, J = 17.2 Hz, 1H), 5.06 (d, J = 8.0 Hz, 1H), 3.74 (s, 3H), 2.88 (d, J = 4.4 Hz, 2H), 2.33 (s, 1H) ppm; ^{13}C{^{1}H} NMR (100 MHz, CDCl$_3$): δ 174.16, 141.45, 136.71, 128.38, 128.07, 127.48, 115.61, 72.81, 52.71, 46.89 ppm. IR (thin film): 3054, 2986, 1732, 1421, 1265, 895, 734, 705 cm^{-1}; HRMS calcd for $C_{18}H_{20}NO_2^+$ 282.1494, observed 282.1494 [M+H]$^+$.

Methyl 2,2-diphenyl-2-(phenylamino)acetate (3k).

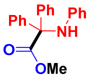

The reaction was performed following the Typical Experimental Procedure with N,1,1-triphenylmethanimine (51.5 mg, 0.2 mmol), catalyst **Ir-7** (0.9 mg, 0.5 mol %), Cy$_2$NMe (85.6 µL, 0.4 mml), and CO_2 (in balloon) in 2 mL MeCN. Upon completion, the amino acid product was converted into its methyl ester. The crude product was purified by chromatography on silica gel (eluted with petroleum ether/EtOAc = 30/1) to give the desired product (50.8 mg, 80% yield) as white solid.

m.p 103.1-104.2 °C R$_f$ = 0.32 (hexanes : ethyl acetate = 20 : 1). ^{1}H NMR (400 MHz, CDCl$_3$): δ 7.55 (d, J = 8.1 Hz, 4H), 7.33 – 7.24 (m, 6H), 6.99 (t, J = 7.2 Hz, 2H), 6.63 (t, J = 7.3 Hz, 1H), 6.43 (d, J = 8.0 Hz, 2H), 5.42 (s, 1H), 3.68 (s, 3H) ppm; ^{13}C{^{1}H} NMR (100 MHz, CDCl$_3$): δ 173.81, 145.33, 140.21, 128.69, 128.50, 128.29, 127.79, 118.17, 115.67, 71.68, 53.27 ppm. IR (thin film): 1737, 1491, 1234, 1201, 1014, 757, 709, 698 cm^{-1}; HRMS calcd for $C_{21}H_{20}NO_2^+$ 318.1494, observed 318.1496 [M+H]$^+$.

Methyl 2-((2-fluorophenyl)amino)-2,2-diphenylacetate (3l).

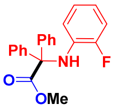

The reaction was performed following the Typical Experimental Procedure with N-(2-fluorophenyl)-1,1-diphenylmethanimine (55.1 mg, 0.2 mmol), catalyst **Ir-7** (0.9 mg, 0.5 mol %), Cy$_2$NMe (85.6 µL, 0.4 mml), and CO_2 (in

balloon) in 2 mL MeCN. Upon completion, the amino acid product was converted into its methyl ester. The crude product was purified by chromatography on silica gel (eluted with petroleum ether/EtOAc = 30/1) to give the desired product (55.0 mg, 82% yield) as white solid.

m.p 116.0-116.2 °C R_f = 0.40(hexanes : ethyl acetate = 20 : 1). ^{1}H NMR (400 MHz, $CDCl_3$): δ 7.54 (d, J = 8.0 Hz, 4H), 7.36 – 7.24 (m, 6H), 7.09 – 6.82 (m, 1H), 6.72 – 6.43 (m, 2H), 6.16 (t, J = 8.4 Hz, 1H), 5.72 (s, 1H), 3.70 (s, 3H) ppm; $^{13}C\{^1H\}$ NMR (100 MHz, $CDCl_3$): δ 173.44, 152.27 (d, J = 239.1 Hz), 139.81, 133.72 (d, J = 10.6 Hz), 128.43, 128.39, 127.96, 123.67, 117.78, 116.07, 114.41 (d, J = 19.0 Hz), 71.39, 53.39 ppm. IR (thin film): 1729, 1618, 1510, 1448, 1259, 1198, 743, 706 cm^{-1}; HRMS calcd for $C_{21}H_{19}FNO_2^+$ 336.1400, observed 336.1435 $[M+H]^+$.

Methyl 2-((3-chlorophenyl)amino)-2,2-diphenylacetate (**3m**).

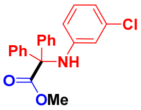

The reaction was performed following the Typical Experimental Procedure with N-(3-chlorophenyl)-1,1-diphenylmethanimine (58.4mg, 0.2 mmol), catalyst **Ir-7** (0.9 mg, 0.5 mol %), Cy_2NMe (85.6 µL, 0.4 mml), and CO_2 (in balloon) in 2 mL MeCN. Upon completion, the amino acid product was converted into its methyl ester. The crude product was purified by chromatography on silica gel (eluted with petroleum ether/EtOAc = 30/1) to give the desired product (44.3 mg, 63% yield) as white solid.

m.p 122.0-123.7 °C . R_f = 0.31(hexanes : ethyl acetate = 20 : 1). ^{1}H NMR (400 MHz, $CDCl_3$): δ 7.52 (d, J = 7.7 Hz, 4H), 7.43 – 7.21 (m, 6H), 6.85 (td, J = 8.2, 1.9 Hz, 1H), 6.58 (d, J = 7.9 Hz, 1H), 6.45 (d, J = 1.9 Hz, 1H), 6.24 (d, J = 8.2 Hz, 1H), 5.63 (s, 1H), 3.71 (s, 3H) ppm; $^{13}C\{^1H\}$ NMR (100 MHz, $CDCl_3$): δ 173.56, 146.33, 139.23, 134.31, 129.63, 128.61, 128.40, 128.04, 118.07, 115.63, 113.68, 71.51, 53.51 ppm. IR (thin film): 3387, 1724, 1595, 1500, 1482, 1249, 1238, 698 cm^{-1}; HRMS calcd for $C_{21}H_{19}ClNO_2^+$ 352.1104, observed 352.1107 $[M+H]^+$.

Mthyl 2-((4-chlorophenyl)amino)-2,2-diphenylacetate (**3n**).

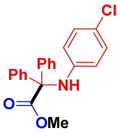

The reaction was performed following the Typical Experimental Procedure with N-(4-chlorophenyl)-1,1-diphenylmethanimine (58.4 mg, 0.2 mmol), catalyst **Ir-7** (0.9 mg, 0.5 mol %), Cy_2NMe (85.6 µL, 0.4 mml), and CO_2 (in balloon) in 2 mL MeCN. Upon completion, the amino acid product was converted into its methyl ester. The crude product was purified by chromatography on silica gel (eluted with petroleum ether/EtOAc = 30/1) to give the desired product (47.1 mg, 67% yield) as white solid.

m.p 135.4-136.1 °C R_f = 0.33 (hexanes : ethyl acetate = 20 : 1). ^{1}H NMR (400 MHz, $CDCl_3$): δ 7.52 (d, J = 7.9 Hz, 4H), 7.42 – 7.17 (m, 6H), 6.92 (d, J = 7.0 Hz, 2H), 6.34 (d, J = 7.0 Hz, 2H), 5.52 (s, 1H), 3.69 (s, 3H) ppm; $^{13}C\{^1H\}$ NMR (100 MHz, $CDCl_3$): δ 173.60, 143.79, 139.52, 128.56, 128.53, 128.37, 127.98, 122.89, 116.77, 71.61, 53.41 ppm. IR (thin film): 3371, 1725, 1493, 1262, 1238, 1170, 825, 723 cm^{-1}; HRMS calcd for $C_{21}H_{19}ClNO_2^+$ 352.1104, observed 352.1100 $[M+H]^+$.

Methyl 2,2-diphenyl-2-((4-(trifluoromethyl)phenyl)amino)acetate (**3o**).

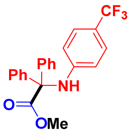

The reaction was performed following the Typical Experimental Procedure with 1,1-diphenyl-N-(4-(trifluoromethyl)phenyl)methanimine (65.1 mg, 0.2 mmol), catalyst **Ir-7** (0.9 mg, 0.5 mol %), Cy_2NMe (85.6 µL, 0.4 mml), and CO_2 (in balloon) in 2 mL MeCN. Upon completion, the amino acid product was converted into its methyl ester. The crude product was purified by chromatography on silica gel (eluted with petroleum ether/EtOAc = 30/1) to give the desired product (52.4 mg, 68% yield) as white solid.

m.p 109.9-110.2 °C R_f = 0.29 (hexanes : ethyl acetate = 20 : 1). ^{1}H NMR (400 MHz, $CDCl_3$): δ 7.63 – 7.47 (m, 4H), 7.40 – 7.28 (m, 6H), 7.20 (d, J = 8.6 Hz, 2H), 6.43 (d, J = 8.6 Hz, 2H), 5.91 (s, 1H), 3.72 (s, 3H) ppm; ^{13}C{^{1}H} NMR (100 MHz, $CDCl_3$): δ 173.48, 147.72, 138.72, 128.62, 128.49, 128.18, 126.04 (q, J = 3.6 Hz), 124.92 (q, J = 269.0 Hz), 119.49 (q, J = 32.7 Hz), 114.73, 71.26, 53.68 ppm. IR (thin film): 3368, 1728, 1616, 1325, 1275, 1242, 1110, 1063 cm^{-1}; HRMS calcd for $C_{22}H_{19}F_3NO_2^+$ 386.1368, observed 386.1369 $[M+H]^+$.

Methyl 2,2-diphenyl-2-(pyridin-3-ylamino)acetate (**3p**).

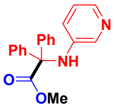

The reaction was performed following the Typical Experimental Procedure with 1,1-diphenyl-N-(pyridin-3-yl)methanimine (51.7 mg, 0.2 mmol), catalyst **Ir-7** (0.9 mg, 0.5 mol %), Cy_2NMe (85.6 µL, 0.4 mml), and CO_2 (in balloon) in 2 mL MeCN. Upon completion, the amino acid product was converted into its methyl ester. The crude product was purified by chromatography on silica gel (eluted with petroleum ether/EtOAc = 3/1) to give the desired product (46.5 mg, 73% yield) as colorless oil.

R_f = 0.65 (hexanes : ethyl acetate = 1 : 1). ^{1}H NMR (400 MHz, $CDCl_3$): δ 7.97 (d, J = 2.9 Hz, 1H), 7.87 (dd, J = 4.8, 1.0 Hz, 1H), 7.52 (d, J = 7.0 Hz, 4H), 7.44 – 7.27 (m, 6H), 6.82 (dd, J = 8.4, 4.7 Hz, 1H), 6.55 (dd, J = 8.3, 3.7 Hz, 1H), 5.65 (s, 1H), 3.73 (s, 3H) ppm; ^{13}C{^{1}H} NMR (100 MHz, $CDCl_3$): δ 173.50, 141.24, 139.49, 138.76, 138.70, 128.64, 128.49, 128.19, 123.00, 121.42, 71.39, 53.63 ppm. IR (thin film): 3054, 2986, 1732, 1587, 1421, 1265, 737, 705 cm^{-1}; HRMS calcd for $C_{20}H_{19}N_2O_2^+$ 319.1447, observed 319.1449 $[M+H]^+$.

Methyl 2-(benzylamino)-2-(2-fluorophenyl)-2-phenylacetate (**3q**).

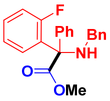

The reaction was performed following the Typical Experimental Procedure with N-benzyl-1-(2-fluorophenyl)-1-phenylmethanimine (57.9 mg, 0.2 mmol), catalyst **Ir-7** (0.9 mg, 0.5 mol %), Cy_2NMe (85.6 µL, 0.4 mml), and CO_2 (in balloon) in 2 mL MeCN. Upon completion, the amino acid product was converted into its methyl ester. The crude product

was purified by chromatography on silica gel (eluted with petroleum ether/EtOAc = 30/1) to give the desired product (60.1 mg, 86% yield) as white solid.

m.p 104.9-105.1 °C R_f = 0.35(hexanes : ethyl acetate = 20 : 1). ^{1}H NMR (400 MHz, $CDCl_3$): δ 7.76 (d, J = 7.7 Hz, 2H), 7.47 – 7.43 (m, 1H), 7.40 – 7.22 (m, 9H), 7.12 – 6.95 (m, 2H), 3.77 (s, 3H), 3.68 – 3.35 (m, 2H), 2.48 (s, 1H) ppm; $^{13}C\{^1H\}$ NMR (100 MHz, $CDCl_3$): 173.09, 160.60 (d, J = 247.2 Hz), 139.74 (d, J = 92.3 Hz), 130.40 (d, J = 11.6 Hz), 129.46 (d, J = 8.8 Hz), 129.24 (d, J = 3.6 Hz), 128.67 (d, J = 9.3 Hz), 128.55, 128.35 (d, J = 1.3 Hz), 128.24, 128.12, 127.83, 127.16, 123.95, 115.98 (d, J = 22.9 Hz), 69.24, 52.77, 48.04 ppm. IR (thin film): 1748, 1455, 1228, 1192, 1016, 753, 722, 696 cm^{-1}; HRMS calcd for $C_{22}H_{21}FNO_2^+$ 350.1556, observed 350.1556 $[M+H]^+$.

Methyl 2-(benzylamino)-2-(2-chlorophenyl)-2-phenylacetate (**3r**).

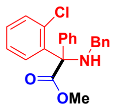

The reaction was performed following the Typical Experimental Procedure with N-benzyl-1-(2-chlorophenyl)-1-phenylmethanimine (61.2 mg, 0.2 mmol), catalyst **Ir-7** (0.9 mg, 0.5 mol %), Cy_2NMe (85.6 µL, 0.4 mml), and CO_2 (in balloon) in 2 mL MeCN. Upon completion, the amino acid product was converted into its methyl ester. The crude product was purified by chromatography on silica gel (eluted with petroleum ether/EtOAc = 30/1) to give the desired product (65.1 mg, 89% yield) as colorless oil.

R_f = 0.36 (hexanes : ethyl acetate = 20 : 1). ^{1}H NMR (400 MHz, $CDCl_3$): δ 7.83 (d, J = 7.6 Hz, 2H), 7.49 (dd, J = 6.0, 3.3 Hz, 1H), 7.41 – 7.15 (m, 11H), 3.73 (s, 3H), 3.51 (d, J = 13.0 Hz, 1H), 3.20 (d, J = 13.0 Hz, 1H), 2.72 (s, 1H) ppm; $^{13}C\{^1H\}$ NMR (100 MHz, $CDCl_3$): δ 172.93, 140.16, 138.46, 133.96, 130.87, 130.42, 129.10, 128.92, 128.51, 128.15, 128.14, 127.89, 127.12, 126.47, 71.59, 52.85, 48.17 ppm. IR (thin film): 3368, 3054, 1736, 1433, 1265, 1239, 739, 704 cm^{-1}; HRMS calcd for $C_{22}H_{21}ClNO_2^+$ 366.1261, observed 366.1261 $[M+H]^+$.

Methyl 2-(benzylamino)-2,2-bis(4-chlorophenyl)acetate (**3s**).

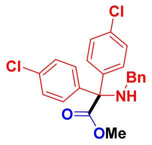

The reaction was performed following the Typical Experimental Procedure with N-benzyl-1,1-bis(4-chlorophenyl)methanimine (68.1 mg, 0.2 mmol), catalyst **Ir-7** (0.9 mg, 0.5 mol %), Cy_2NMe (85.6 µL, 0.4 mml), and CO_2 (in balloon) in 2 mL MeCN. Upon completion, the amino acid product was converted into its methyl ester. The crude product was purified by chromatography on silica gel (eluted with petroleum ether/EtOAc = 30/1) to give the desired product (60.0 mg, 75% yield) as colorless oil.

R_f = 0.42 (hexanes : ethyl acetate = 20 : 1). ^{1}H NMR (400 MHz, $CDCl_3$): δ 7.47 (d, J = 8.8 Hz, 4H), 7.40 – 7.23 (m, 10H), 3.80 (s, 3H), 3.43 (s, 2H), 2.56 (s, 1H) ppm; $^{13}C\{^1H\}$ NMR (100 MHz, $CDCl_3$): δ 173.43, 139.87, 139.80, 133.65, 129.60, 128.60, 128.47, 128.16, 127.31, 72.09, 53.02, 48.44 ppm. IR (thin film): 3053, 1734, 1490, 1265, 1094, 1013, 739, 704 cm^{-1}; HRMS calcd for $C_{22}H_{20}Cl_2NO_2^+$ 400.0871, observed 400.0872 $[M+H]^+$.

Methyl 2-(benzylamino)-2-phenyl-2-(p-tolyl)acetate (**3t**).

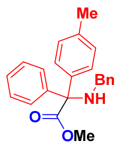

The reaction was performed following the Typical Experimental Procedure with N-benzyl-1-phenyl-1-(p-tolyl)methanimine (57.1mg, 0.2 mmol), catalyst **Ir-7** (0.9 mg, 0.5 mol %), Cy_2NMe (85.6 μL, 0.4 mml), and CO_2 (in balloon) in 2 mL MeCN. Upon completion, the amino acid product was converted into its methyl ester. The crude product was purified by chromatography on silica gel (eluted with petroleum ether/EtOAc = 30/1) to give the desired product (56.7 mg, 82% yield) as colorless oil.

R_f = 0.34 (hexanes : ethyl acetate = 10 : 1). ^{1}H NMR (400 MHz, $CDCl_3$): δ 7.53 (dd, J = 8.3, 1.2 Hz, 2H), 7.44 – 7.21 (m, 10H), 7.14 (d, J = 8.0 Hz, 2H), 3.77 (s, 3H), 3.44 (s, 2H), 2.54 (brs, 1H), 2.33 (s, 3H) ppm; $^{13}C\{^1H\}$ NMR (100 MHz, $CDCl_3$): δ 174.32, 141.71, 140.54, 138.61, 137.24, 128.88, 128.49, 128.38, 128.29, 128.25, 128.13, 127.48, 127.06, 72.79, 52.76, 48.41, 21.19 ppm. IR (thin film): 3054, 1731, 1599, 1421, 1265, 739, 705 cm^{-1}; HRMS calcd for $C_{23}H_{24}NO_2^+$ 346.1807, observed 346.1801 $[M+H]^+$.

Methyl 2-(benzylamino)-2-(4-methoxyphenyl)-2-phenylacetate (**3u**).

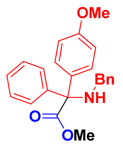

The reaction was performed following the Typical Experimental Procedure with N-benzyl-1-(4-methoxyphenyl)-1-phenylmethanimine (60.3 mg, 0.2 mmol), catalyst **Ir-7** (0.9 mg, 0.5 mol %), Cy_2NMe (85.6 μL, 0.4 mml), and CO_2 (in balloon) in 2 mL MeCN. Upon completion, the amino acid product was converted into its methyl ester. The crude product was purified by chromatography on silica gel (eluted with petroleum ether/EtOAc = 30/1) to give the desired product (54.6 mg, 78% yield) as colorless oil.

R_f = 0.41 (hexanes : ethyl acetate = 10 : 1). ^{1}H NMR (400 MHz, $CDCl_3$): δ 7.50 (d, J = 7.2 Hz, 2H), 7.45 (d, J = 8.9 Hz, 2H), 7.38 – 7.22 (m, 8H), 6.86 (d, J = 8.9 Hz, 2H), 3.80 (s, 3H), 3.77 (s, 3H), 3.43 (s, 2H), 2.53 (brs, 1H) ppm; $^{13}C\{^1H\}$ NMR (100 MHz, $CDCl_3$): δ 174.40, 158.84, 141.90, 140.54, 133.52, 129.71, 128.49, 128.29, 128.25, 128.16, 127.50, 127.07, 113.43, 72.52, 55.37, 52.78, 48.42 ppm. IR (thin film): 3435, 3054, 2986, 1421, 1265, 895, 740, 705 cm^{-1}; HRMS calcd for $C_{23}H_{24}NO_3^+$ 362.1756, observed362.1754 $[M+H]^+$.

Methyl 2-(benzylamino)-2-phenyl-2-(thiophen-2-yl)acetate (**3v**).

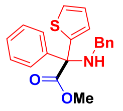

The reaction was performed following the Typical Experimental Procedure with N-benzyl-1-phenyl-1-(thiophen-2-yl)methanimine (55.5 mg, 0.2 mmol), catalyst **Ir-7** (0.9 mg, 0.5 mol %), Cy_2NMe (85.6 μL, 0.4 mml), and CO_2 (in balloon) in 2 mL MeCN. Upon completion, the amino acid product was converted into its methyl ester. The crude product was

purified by chromatography on silica gel (eluted with petroleum ether/EtOAc = 30/1) to give the desired product (49.9mg, 74% yield) as colorless oil.

R$_f$ = 0.37 (hexanes : ethyl acetate = 20 : 1). ^{1}H NMR (400 MHz, $CDCl_3$): δ 7.50 (d, J = 7.4 Hz, 2H), 7.39 (d, J = 7.4 Hz, 2H), 7.35 – 7.24 (m, 8H), 6.99 (dd, J = 4.9, 3.8 Hz, 1H), 3.80 (s, 3H), 3.57 (d, J = 6.4 Hz, 2H), 2.77 (s, 1H) ppm; ^{13}C{^{1}H} NMR (100 MHz, $CDCl_3$): δ 173.44, 146.02, 142.07, 140.23, 128.49, 128.45, 128.35, 127.96, 127.49, 127.16, 127.06, 126.83, 125.72, 70.89, 53.00, 48.81 ppm. IR (thin film): 3431, 3054, 1733, 1636, 1434, 1265, 741, 704 cm^{-1}; HRMS calcd for $C_{20}H_{20}NO_2S^+$ 338.1215, observed 338.1206 [M+H]$^+$.

Methyl 2-(benzylamino)-2-(furan-2-yl)-2-phenylacetate (**3w**).

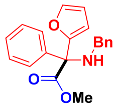

The reaction was performed following the Typical Experimental Procedure with N-benzyl-1-(furan-2-yl)-1-phenylmethanimine (52.3 mg, 0.2 mmol), catalyst **Ir-7** (0.9 mg, 0.5 mol %), Cy_2NMe (85.6 μL, 0.4 mml), and CO_2 (in balloon) in 2 mL MeCN. Upon completion, the amino acid product was converted into its methyl ester. The crude product was purified by chromatography on silica gel (eluted with petroleum ether/EtOAc = 30/1) to give the desired product (45.6 mg, 71% yield) as colorless oil.

R$_f$ = 0.27 (hexanes : ethyl acetate = 20 : 1). ^{1}H NMR (400 MHz, $CDCl_3$): δ 7.59 (d, J = 8.4 Hz, 2H), 7.43 (s, 1H), 7.38 – 7.25 (m, 8H), 6.47 (d, J = 3.5 Hz, 1H), 6.38 (dd, J = 3.1, 1.8 Hz, 1H), 3.76 (s, 3H), 3.52 (s, 2H), 2.62 (s, 1H) ppm; ^{13}C{^{1}H} NMR (100 MHz, $CDCl_3$): δ 172.26, 153.72, 142.58, 140.30, 139.18, 128.49, 128.39, 128.38, 128.24, 127.80, 127.12, 110.39, 110.10, 69.46, 53.07, 48.62 ppm. IR (thin film): 3446, 3054, 2986, 1421, 1265, 895, 737, 705 cm^{-1}; HRMS calcd for $C_{20}H_{20}NO_3^+$ 322.1443, observed 322.1439 [M+H]$^+$.

Methyl 2-(naphthalen-2-yl)-2-(phenylamino)propanoate (**3x**).

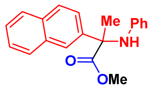

The reaction was performed following the Typical Experimental Procedure with 1-(naphthalen-2-yl)-N-phenylethan-1-imine (49 mg, 0.2 mmol), catalyst **Ir-7** (3.6 mg, 2 mol %), Cy_2NMe (85.6 μL, 0.4 mml), and CO_2 (in balloon) in 2 mL MeCN. Upon completion, the amino acid product was converted into its methyl ester. The crude product was purified by chromatography on silica gel (eluted with petroleum ether/EtOAc = 30/1) to give the desired product (37.2 mg, 61% yield) as colorless oil.

R$_f$ = 0.43 (hexanes : ethyl acetate = 20 : 1). ^{1}H NMR (400 MHz, $CDCl_3$): δ 8.05 (s, 1H), 7.88 – 7.79 (m, 3H), 7.68 (dd, J = 8.7, 1.9 Hz, 1H), 7.51 – 7.45 (m, 2H), 6.99 (t, J = 7.6 Hz, 2H), 6.63 (t, J = 7.3 Hz, 1H), 6.43 (d, J = 7.7 Hz, 2H), 5.34 (s, 1H), 3.66 (s, 3H), 2.09 (s, 3H) ppm; ^{13}C{^{1}H} NMR (100 MHz, $CDCl_3$): δ 175.18, 144.72, 138.96, 133.45, 132.86, 129.01, 128.56, 128.49, 127.68, 126.36, 126.28, 126.06, 124.95, 117.89, 115.51, 63.27, 53.35, 22.95 ppm. IR (thin film): 3054, 1732, 1602, 1504, 1434, 1271, 1120, 703 cm^{-1}; HRMS calcd for $C_{20}H_{20}NO_2^+$ 306.1494, observed 306.1495 [M+H]$^+$.

Typical Experimental Procedure to Prepare 4

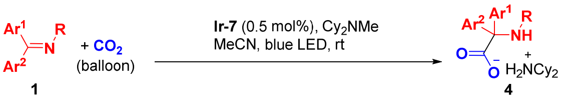

Ketimine **1** (0.2 mmol), catalyst **Ir-7** (0.9 mg, 0.5 mol %), Cy_2NMe (85.6 µL, 0.4 mmol), MeCN (2 mL) and a magnetic stirring bar were charged into an oven-dried 5 mL vial under nitrogen. The vial was sealed with a septum. CO_2 gas in a balloon was bubbled into the mixture under stirring for 30 seconds through a needle, which was then lifted up out of the solution and was kept in the vial. The mixture was placed under a 20 W blue LED light source and stirred at ambient temperature (15—20 °C). White precipitates appeared as the reaction proceeded. Upon completion of the reaction as monitored by TLC, the vial was opened and cooled down in an ice bath. The precipitates were collected by filtration, and washed using cold MeCN (3 X 0.4 mL). The desired compound **4** was obtained after drying under reduced pressure.

2-(benzylamino)-2,2-diphenylacetic acid dicyclohexylamine salt (4a)

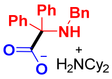

The reaction was performed following the Typical Experimental Procedure with ketimine N-benzyl-1,1-diphenylmethanimine (54.2 mg, 0.2 mmol), catalyst **Ir-7** (0.9 mg, 0.5 mol %), Cy_2NMe (85.6 µL, 0.4 mml), and CO_2 (in balloon) in 2 mL MeCN. Upon completion, the desired product was obtained by filtration as white solid.

m.p 154.9-155.5 °C (90.8 mg, 91% yield). ^1H NMR (400 MHz, $CDCl_3$): δ 7.60 (d, J = 7.2 Hz, 4H), 7.31 (d, J = 7.0 Hz, 2H), 7.28 – 7.22 (m, 6H), 7.21 – 7.14 (m, 3H), 3.31 (s, 2H), 2.66 (t, J = 10.8 Hz, 2H, Cy), 1.85 – 1.43 (m, 10H, Cy), 1.34 – 0.81 (m, 10H, Cy) ppm; $^{13}C\{^1H\}$ NMR (100 MHz, $CDCl_3$): δ 177.06, 144.50, 141.82, 129.44, 128.47, 128.20, 127.23, 126.57, 126.11, 74.16, 52.25 (Cy), 48.80, 28.95 (Cy), 25.17 (Cy), 24.87 (Cy) ppm. IR (thin film): 2928, 2853, 1632, 1360, 1347, 1117, 1098, 702 cm^{-1}; HRMS (2-(benzylamino)-2,2-diphenylacetic acid) calcd for $C_{21}H_{20}NO_2^+$ 318.1494, observed 318.1492 $[M+H]^+$. HRMS (dicyclohexylamine) calcd for $C_{12}H_{24}N^+$ 182.1909, observed 182.1907 $[M+H]^+$.

2-((2-chlorobenzyl)amino)-2,2-diphenylacetic acid dicyclohexylamine salt (4b)

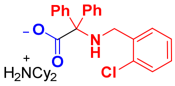

The reaction was performed following the Typical Experimental Procedure with ketimine N-(2-chlorobenzyl)-1,1-diphenylmethanimine (61.2 mg, 0.2 mmol), catalyst **Ir-7** (0.9 mg, 0.5 mol %), Cy_2NMe (85.6 µL, 0.4 mml), and CO_2 (in balloon) in 2 mL MeCN. Upon completion, the desired product was obtained by filtration as white solid.

m.p 138.5-139.6 °C (94.9 mg, 89% yield). ^1H NMR (400 MHz, $CDCl_3$): δ 7.62 (d, J = 7.2 Hz, 4H), 7.53 (dd, J = 7.6, 1.7 Hz, 1H), 7.30 – 7.22 (m, 5H), 7.22 – 7.09 (m, 4H), 3.40 (s, 2H), 2.67 (t, J = 10.9 Hz, 2H, Cy), 1.81 – 1.28 (m, 10H, Cy), 1.20 – 0.70 (m, 10H, Cy) ppm; $^{13}C\{^1H\}$ NMR (100 MHz, $CDCl_3$): δ 7.62 (d, J = 7.2 Hz, 4H), 7.53 (dd, J = 7.6, 1.7 Hz, 1H), 7.30 – 7.22 (m, 5H), 7.22 – 7.09 (m, 4H), 3.40 (s, 2H), 2.67 (t, J = 10.9 Hz, 2H, Cy), 1.81 – 1.28 (m, 10H, Cy), 1.20 – 0.70 (m, 10H, Cy) ppm. IR (thin film): 2940, 1626, 1566, 1445, 1359, 754, 709, 699 cm^{-1}; HRMS (2-((2-chlorobenzyl)amino)-2,2-diphenylacetic acid) calcd for $C_{21}H_{19}ClNO_2^+$ 352.1104, observed 352.1102 $[M+H]^+$. HRMS (dicyclohexylamine) calcd for $C_{12}H_{24}N^+$ 182.1909, observed 182.1906 $[M+H]^+$.

2-((3-chlorobenzyl)amino)-2,2-diphenylacetic acid dicyclohexylamine salt (4c)

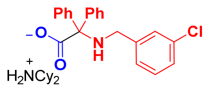

The reaction was performed following the Typical Experimental Procedure with ketimine N-(3-chlorobenzyl)-1,1-diphenylmethanimine (61.2 mg, 0.2 mmol), catalyst **Ir-7** (0.9 mg, 0.5 mol %), Cy_2NMe (85.6 µL, 0.4 mml), and CO_2 (in balloon) in 2 mL MeCN. Upon completion, the desired product was obtained by filtration as white solid.

m.p 148.0-149.6 °C (96.0 mg, 90% yield). ^{1}H NMR (400 MHz, $CDCl_3$): δ 7.58 (d, J = 7.4 Hz, 4H), 7.36 – 7.11 (m, 10H), 3.27 (s, 2H), 2.66 (brs, 2H, Cy), 1.84 – 1.45 (m, 10H, Cy), 1.16 – 0.93 (m, 10H, Cy) ppm; ^{13}C{^{1}H} NMR (100 MHz, $CDCl_3$): δ 176.95, 144.33, 144.05, 133.98, 129.41 (x2), 128.42, 127.28, 126.67, 126.59, 126.21, 74.08, 52.28 (Cy), 48.39, 28.90 (Cy), 25.17 (Cy), 24.85 (Cy) ppm. IR (thin film): 2935, 1627, 1551, 1447, 1358, 1349, 810, 705cm^{-1}; HRMS (2-((3-chlorobenzyl)amino)-2,2-diphenylacetic acid) calcd for $C_{21}H_{19}ClNO_2^+$ 352.1104, observed 352.1105 [M+H]$^+$. HRMS (dicyclohexylamine) calcd for $C_{12}H_{24}N^+$ 182.1909, observed 182.1906 [M+H]$^+$.

2-((4-fluorobenzyl)amino)-2,2-diphenylacetic acid dicyclohexylamine salt (4d)

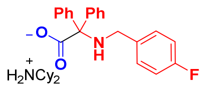

The reaction was performed following the Typical Experimental Procedure with ketimine N-(4-fluorobenzyl)-1,1-diphenylmethanimine (57.9 mg, 0.2 mmol), catalyst **Ir-7** (0.9 mg, 0.5 mol %), Cy_2NMe (85.6 µL, 0.4 mml), and CO_2 (in balloon) in 2 mL MeCN. Upon completion, the desired product was obtained by filtration as white solid.

m.p 155.7-156.3 °C (67.2 mg, 65% yield). ^{1}H NMR (400 MHz, $CDCl_3$): δ 7.59 (d, J = 7.9 Hz, 4H), 7.22 (dt, J = 29.1, 7.2 Hz, 8H), 6.92 (t, J = 8.4 Hz, 2H), 3.26 (s, 2H), 2.66 (brs, 2H, Cy), 1.87 – 1.36 (m, 10H, Cy), 1.02 (d, J = 54.4 Hz, 10H, Cy) ppm; ^{13}C{^{1}H} NMR (100 MHz, $CDCl_3$): δ 176.98, 161.76 (d, J = 243.7 Hz), 144.39, 137.50, 129.89 (d, J = 7.8 Hz), 129.42, 127.24, 126.18, 114.87 (d, J = 21.1 Hz), 74.17, 52.26 (Cy), 48.09, 28.85 (Cy), 25.14 (Cy), 24.86 (Cy) ppm. IR (thin film): 2931, 1629, 1507, 1361, 1350, 1219, 759, 702 cm^{-1}; HRMS (2-((4-fluorobenzyl)amino)-2,2-diphenylacetic acid) calcd for $C_{21}H_{19}FNO_2^+$ 336.1400, observed 336.1403 [M+H]$^+$ HRMS (dicyclohexylamine) calcd for $C_{12}H_{24}N^+$ 182.1909, observed 182.1908 [M+H]$^+$.

2,2-diphenyl-2-((4-(trifluoromethyl)benzyl)amino)acetic acid dicyclohexylamine salt (4e)

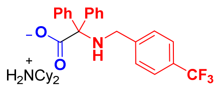

The reaction was performed following the Typical Experimental Procedure with ketimine 1,1-diphenyl-N-(4-(trifluoromethyl)benzyl)methanimine (67.9 mg, 0.2 mmol), catalyst **Ir-7** (0.9 mg, 0.5 mol %), Cy_2NMe (85.6 µL, 0.4 mml), and CO_2 (in balloon) in 2 mL MeCN. Upon completion, the desired product was obtained by filtration as white solid.

m.p 138.2-140.1 °C (74.8mg, 66% yield). ^{1}H NMR (400 MHz, $CDCl_3$): δ 7.58 (d, J = 7.4 Hz, 4H), 7.50 (d, J = 8.2 Hz, 2H), 7.42 (d, J = 8.1 Hz, 2H), 7.26 (t, J = 7.5 Hz, 4H), 7.19 (t, J = 7.2 Hz, 2H), 3.36 (s, 2H), 2.66 (t, J = 10.5 Hz, 2H, Cy), 1.86 – 1.40 (m, 10H, Cy), 1.26 – 0.81 (m, 10H, Cy) ppm; ^{13}C{^{1}H} NMR (100 MHz, $CDCl_3$): δ 176.96, 146.07, 144.25, 129.40,

128.77 (q, J = 31.8 Hz), 128.53, 127.31, 126.29, 125.07 (q, J = 3.6 Hz), 124.46 (q, J = 273.0 Hz), 74.13, 52.32 (Cy), 48.41, 28.80 (Cy), 25.11 (Cy), 24.83 (Cy) ppm. IR (thin film): 2929, 2857, 1630, 1362, 1326, 1123, 1066, 705cm^{-1}; HRMS (2,2-diphenyl-2-((4-(trifluoromethyl)benzyl)amino)acetic acid) calcd for $C_{22}H_{19}F_3NO_2^+$ 386.1368, observed 386.1363 $[M+H]^+$. HRMS (dicyclohexylamine) calcd for $C_{12}H_{24}N^+$ 182.1909, observed 182.1906 $[M+H]^+$.

2-((4-methylbenzyl)amino)-2,2-diphenylacetic acid dicyclohexylamine salt (4f)

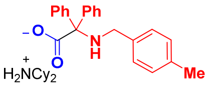

The reaction was performed following the Typical Experimental Procedure with ketimine N-(4-methylbenzyl)-1,1-diphenylmethanimine (57.1 mg, 0.2 mmol), catalyst **Ir-7** (0.9 mg, 0.5 mol %), Cy_2NMe (85.6 μL, 0.4 mml), and CO_2 (in balloon) in 2 mL MeCN. Upon completion, the desired product was obtained by filtration as white solid.

m.p 139.6-140.3 °C (87.2 mg, 85% yield). ^{1}H NMR (400 MHz, $CDCl_3$): δ 7.60 (d, J = 7.2 Hz, 4H), 7.30 – 7.13 (m, 8H), 7.05 (d, J = 7.8 Hz, 2H), 3.27 (s, 2H), 2.66 (t, J = 10.8 Hz, 2H, Cy), 2.30 (s, 3H), 1.81 – 1.43 (m, 10H, Cy), 1.23 – 0.82 (m, 10H, Cy) ppm; ^{13}C{^{1}H} NMR (100 MHz, $CDCl_3$): δ 176.97, 144.56, 138.72, 136.03, 129.45, 128.87, 128.41, 127.20, 126.08, 74.21, 52.29 (Cy), 48.53, 28.94 (Cy), 25.18 (Cy), 24.87 (Cy), 21.21 ppm. IR (thin film): 2942, 1629, 1489, 1456, 1360, 802, 750, 706 cm^{-1}; HRMS (2-((4-methylbenzyl)amino)-2,2-diphenylacetic acid) calcd for $C_{22}H_{22}NO_2^+$ 332.1651, observed 332.1650 $[M+H]^+$. HRMS (dicyclohexylamine) calcd for $C_{12}H_{24}N^+$ 182.1909, observed 182.1907 $[M+H]^+$.

2-((4-(tert-butyl)benzyl)amino)-2,2-diphenylacetic acid dicyclohexylamine salt (4g)

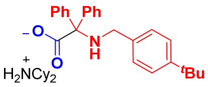

The reaction was performed following the Typical Experimental Procedure with ketimine N-(4-(tert-butyl)benzyl)-1,1-diphenylmethanimine (65.5 mg, 0.2 mmol), catalyst **Ir-7** (0.9 mg, 0.5 mol %), Cy_2NMe (85.6 μL, 0.4 mml), and CO_2 (in balloon) in 2 mL MeCN. Upon completion, the desired product was obtained by filtration as white solid.

m.p 164.1-165.0 °C (79.9mg, 72% yield). ^{1}H NMR (400 MHz, $CDCl_3$): δ 7.60 (d, J = 7.8 Hz, 4H), 7.29 – 7.20 (m, 8H), 7.17 (t, J = 7.2 Hz, 2H), 3.27 (s, 2H), 2.81 – 2.51 (m, 2H, Cy), 1.94 – 1.41 (m, 10H, Cy), 1.29 (s, 9H), 1.16 – 0.93 (m, 10H, Cy) ppm; ^{13}C{^{1}H} NMR (100 MHz, $CDCl_3$): δ 177.05, 149.36, 144.49, 138.79, 129.45, 128.16, 127.22, 126.08, 125.09, 74.15, 52.22 (Cy), 48.33, 34.51, 31.53, 28.88 (Cy), 25.16 (Cy), 24.87 (Cy) ppm. IR (thin film): 2933, 2856, 1633, 1514, 1444, 1362, 729, 699 cm^{-1}; HRMS (2-((4-(tert-butyl)benzyl)amino)-2,2-diphenylacetic acid) calcd for $C_{25}H_{28}NO_2^+$ 374.2120, observed 374.2114$[M+H]^+$. HRMS (dicyclohexylamine) calcd for $C_{12}H_{24}N^+$ 182.1909, observed 182.1907 $[M+H]^+$.

2,2-diphenyl-2-((pyridin-2-ylmethyl)amino)acetic acid dicyclohexylamine salt (4h)

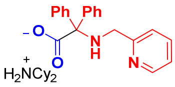

The reaction was performed following the Typical Experimental Procedure with ketimine 1,1-diphenyl-N-(pyridin-2-ylmethyl)methanimine (54.5 mg, 0.2 mmol), catalyst **Ir-7** (0.9 mg, 0.5 mol %), Cy_2NMe (85.6 μL, 0.4 mml), and CO_2 (in balloon) in 2 mL MeCN. Upon completion, the desired product was obtained by filtration as white solid.

m.p 136.5-137.1 °C (91.9 mg, 92% yield). ^{1}H NMR (400 MHz, $CDCl_3$): δ 8.65 – 8.30 (m, 1H), 7.59 (d, *J* = 8.5 Hz, 5H), 7.33 – 7.06 (m, 8H), 3.48 (s, 2H), 2.69 (t, *J* = 10.6 Hz, 2H, Cy), 1.80 – 1.51 (m, 10H, Cy), 1.19 – 0.92 (m, 10H, Cy) ppm; ^{13}C{^{1}H} NMR (100 MHz, $CDCl_3$): δ 176.83, 160.98, 148.85, 144.08, 136.22, 129.54, 127.25, 126.17, 122.39, 121.54, 74.13, 52.26 (Cy), 50.09, 29.01 (Cy), 25.13 (Cy), 24.89 (Cy) ppm. IR (thin film): 2927, 2852, 1624, 1455, 1446, 1117, 1098, 698 cm^{-1}; HRMS (2,2-diphenyl-2-((pyridin-2-ylmethyl)amino)acetic acid) calcd for $C_{20}H_{19}N_2O_2^+$ 319.1447, observed 319.1448 $[M+H]^+$. HRMS (dicyclohexylamine) calcd for $C_{12}H_{24}N^+$ 182.1909, observed 182.1907 $[M+H]^+$.

2,2-diphenyl-2-((pyridin-3-ylmethyl)amino)acetic acid dicyclohexylamine salt (4i)

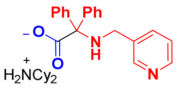

The reaction was performed following the Typical Experimental Procedure with ketimine 1,1-diphenyl-N-(pyridin-3-ylmethyl)methanimine (54.5 mg, 0.2 mmol), catalyst **Ir-7** (0.9 mg, 0.5 mol %), Cy_2NMe (85.6 μL, 0.4 mml), and CO_2 (in balloon) in 2 mL MeCN. Upon completion, the desired product was obtained by filtration as white solid.

m.p 132.2-132.8 °C (77.0 mg, 77% yield). ^{1}H NMR (400 MHz, $CDCl_3$): δ 8.50 (d, *J* = 1.6 Hz, 1H), 8.44 (dd, *J* = 4.8, 1.6 Hz, 1H), 7.77 – 7.50 (m, 5H), 7.31 – 7.16 (m, 7H), 3.31 (s, 2H), 2.67 (brs, 2H, Cy), 1.83 – 1.52 (m, 10H, Cy), 1.17 – 0.93 (m, 10H, Cy) ppm; ^{13}C{^{1}H} NMR (100 MHz, $CDCl_3$): δ 176.90, 149.95, 148.05, 144.17, 137.16, 136.11, 129.41, 127.31, 126.29, 123.24, 74.15, 52.28 (Cy), 46.25, 28.91 (Cy), 25.13 (Cy), 24.83 (Cy) ppm. IR (thin film): 2932, 1632, 1448, 1360, 734, 714, 702, 487 cm^{-1}; HRMS (2,2-diphenyl-2-((pyridin-3-ylmethyl)amino)acetic acid) calcd for $C_{20}H_{19}N_2O_2^+$ 319.1447, observed 319.1445 $[M+H]^+$. HRMS (dicyclohexylamine) calcd for $C_{12}H_{24}N^+$ 182.1909, observed 182.1905 $[M+H]^+$.

2,2-diphenyl-2-((thiophen-2-ylmethyl)amino)acetic acid dicyclohexylamine salt (4j)

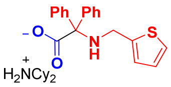

The reaction was performed following the Typical Experimental Procedure with ketimine 1,1-diphenyl-N-(thiophen-2-ylmethyl)methanimine (55.5 mg, 0.2 mmol), catalyst **Ir-7** (0.9 mg, 0.5 mol %), Cy_2NMe (85.6 μL, 0.4 mml), and CO_2 (in balloon) in 2 mL MeCN. Upon completion, the desired product was obtained by filtration as white solid.

m.p 141.6-141.9 °C (90.9 mg, 90% yield). ^{1}H NMR (400 MHz, $CDCl_3$): δ 7.60 (d, *J* = 7.1 Hz, 4H), 7.28 – 7.09 (m, 7H), 6.94 – 6.77 (m, 2H), 3.48 (s, 2H), 2.70 (brs, 2H, Cy), 1.80 – 1.52 (m, 10H, Cy), 1.23 – 0.94 (m, 10H, Cy) ppm; ^{13}C{^{1}H} NMR (100 MHz, $CDCl_3$): δ 176.70, 145.93, 144.38, 129.36, 127.26, 126.49, 126.14, 123.94, 123.68, 73.94, 52.24 (Cy), 43.83, 28.95 (Cy), 25.19 (Cy), 24.89 (Cy) ppm. IR (thin film): 2933, 2855, 1627, 1548, 1447, 1359, 753, 699 cm^{-1}; HRMS (2,2-diphenyl-2-((thiophen-2-ylmethyl)amino)acetic acid) calcd for $C_{19}H_{18}NO_2S^+$ 324.1058, observed 324.1059 $[M+H]^+$. HRMS (dicyclohexylamine) calcd for $C_{12}H_{24}N^+$ 182.1909, observed 182.1908 $[M+H]^+$.

2-((furan-2-ylmethyl)amino)-2,2-diphenylacetic acid dicyclohexylamine salt (4k)

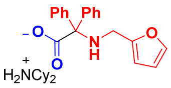

The reaction was performed following the Typical Experimental Procedure with ketimine N-(furan-2-ylmethyl)-1,1-diphenylmethanimine (52.3 mg, 0.2 mmol), catalyst **Ir-7** (0.9 mg, 0.5 mol %), Cy_2NMe (85.6 μL, 0.4 mml), and CO_2 (in balloon) in 2 mL MeCN. Upon completion, the desired product was obtained by filtration as white solid.

m.p 153.8-154.0 °C (92.8 mg, 95% yield). ^1H NMR (400 MHz, $CDCl_3$): δ 7.58 (d, J = 7.2 Hz, 4H), 7.40 – 7.02 (m, 7H), 6.25 (dd, J = 3.1, 1.9 Hz, 1H), 6.17 – 5.95 (m, 1H), 3.27 (s, 2H), 2.70 (brs, 2H), 1.80 – 1.50 (m, 10H, Cy), 1.20 – 0.94 (m, 10H, Cy) ppm; $^{13}C\{^1H\}$ NMR (100 MHz, $CDCl_3$): δ 176.83, 155.34, 144.05, 141.24, 129.46, 127.25, 126.17, 110.15, 106.00, 73.84, 52.21 (Cy), 41.71, 28.85 (Cy), 25.13 (Cy), 24.87 (Cy) ppm. IR (thin film): 2940, 2857, 1625, 1566, 1447, 1359, 720, 697 cm^{-1}; HRMS (2-((furan-2-ylmethyl)amino)-2,2-diphenylacetic acid) calcd for $C_{19}H_{18}NO_3^+$ 308.1287, observed 308.1285 $[M+H]^+$. HRMS (dicyclohexylamine) calcd for $C_{12}H_{24}N^+$ 182.1909, observed 182.1907 $[M+H]^+$.

2-(benzylamino)-2-phenyl-2-(p-tolyl)acetic acid dicyclohexylamine salt (4al)

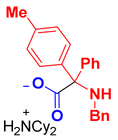

The reaction was performed following the Typical Experimental Procedure with ketimine N-(furan-2-ylmethyl)-1,1-diphenylmethanimine (57.1 mg, 0.2 mmol), catalyst **Ir-7** (0.9 mg, 0.5 mol %), Cy_2NMe (85.6 μL, 0.4 mml), and CO_2 (in balloon) in 2 mL MeCN. Upon completion, the desired product was obtained by filtration as white solid.

m.p 144.0-145.1 °C (77.9 mg, 76% yield). ^1H NMR (400 MHz, $CDCl_3$): δ 7.60 (d, J = 7.9 Hz, 2H), 7.49 (d, J = 7.8 Hz, 2H), 7.37 – 7.13 (m, 8H), 7.06 (d, J = 7.7 Hz, 2H), 3.29 (d, J = 4.3 Hz, 2H), 2.65 (brs, 2H, Cy), 2.31 (s, 3H), 1.77 – 1.47 (m, 10H, Cy), 1.18 – 0.85 (m, 10H, Cy) ppm; $^{13}C\{^1H\}$ NMR (100 MHz, $CDCl_3$): δ 177.14, 144.64, 141.83, 141.38, 135.42, 129.43, 129.36, 128.51, 128.16, 127.94, 127.19, 126.54, 126.03, 73.91, 52.16 (Cy), 48.78, 28.85 (Cy), 25.10 (Cy), 24.87 (Cy), 21.15 ppm. IR (thin film): 2936, 2858, 1633, 1449, 1359, 1346, 775, 700 cm^{-1}; HRMS (2-(benzylamino)-2-phenyl-2-(p-tolyl)acetic acid) calcd for $C_{22}H_{22}NO_2^+$ 332.1651, observed 332.1649 $[M+H]^+$. HRMS (dicyclohexylamine) calcd for $C_{12}H_{24}N^+$ 182.1909, observed 182.1907 $[M+H]^+$.

Gram-Scale Synthesis of 4 Using Visible Light

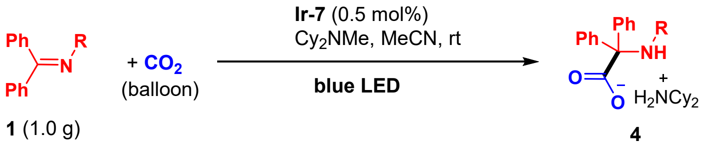

Corresponding ketimine (1.0 g, 3.7 mmol), catalyst **Ir-7** (16.6 mg, 0.0185 mmol, 0.5 mol %), Cy_2NMe (1.58 mL, 7.4 mmol), MeCN (37 mL) and a magnetic stirring bar were charged into an oven-dried 50 mL Schlenk tube under nitrogen. The tube was sealed with a septum. CO_2 gas in a balloon was bubbled into the mixture under stirring for 2 minutes through a needle, which was then lifted up out of the solution and was kept in the tube. The mixture was placed under a 20 W blue LED light source and stirred at ambient temperature (15—20 °C). White precipitates appeared as the reaction proceeded. Upon completion of the reaction as monitored by TLC, the tube was opened and cooled down in an ice bath. The precipitates were collected by filtration, and washed using cold MeCN (3 X 4 mL). The desired compound was obtained after drying under reduced pressure (**4aa**:1.6 g, 87%. **4ak**: 1.7 g, 92%. Supplementary Figure 2).

Gram-Scale Synthesis of 4 Using Sunlight

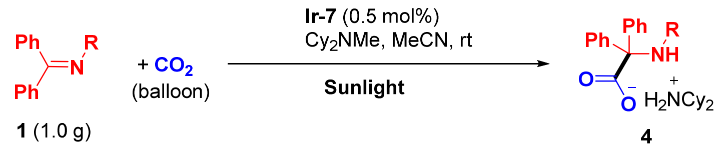

Corresponding ketimine (1.0 g, 3.7 mmol), catalyst **Ir-7** (16.6 mg, 0.0185 mmol, 0.5 mol %), Cy_2NMe (1.58 mL, 7.4 mmol), MeCN (37 mL) and a magnetic stirring bar were charged into an oven-dried 50 mL Schlenk tube under nitrogen. The tube was sealed with a septum. CO_2 gas in a balloon was bubbled into the mixture under stirring for 2 minutes through a needle, which was then lifted up out of the solution and was kept in the tube. The mixture was placed under outdoor sunlight and stirred at ambient temperature (~ 16 °C). White precipitates appeared as the reaction proceeded. Upon completion of the reaction, the tube was opened and cooled down in an ice bath. The precipitates were collected by filtration, and washed using cold MeCN (3 X 4 mL). The desired compound was obtained after drying under reduced pressure (**4aa**:1.7 g, 91%. **4ak**: 1.7 g, 90%. Supplementary Figure 2).

Typical Experimental Procedure to Prepare Iminodiacetic Acid from α-Amino Acid Derivatives

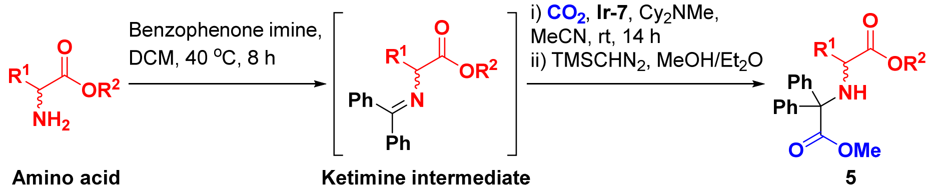

Amino acid ester (0.2 mmol), benzophenone imine (0.2mmol), DCM (0.5ml), and a magnetic stirring bar were charged into an oven-dried 1 mL vial. The mixture was refluxed in an oil bath (40 °C). Upon completion of the reaction as monitored by TLC (approx. 8 hs), the solvent was removed using a rotary evaporator under reduced pressure to give the crude ketimine intermediate, which was used directly in next step. The crude residue was dissolved in anhydrous MeCN (2 mL) in an oven-dried 5 mL vial under nitrogen, catalyst **Ir-7** (0.9 mg, 0.5 mol %), Cy_2NMe (85.6 μL, 0.4 mmol), and a magnetic stirring bar were charged. The vial was sealed with a septum. CO_2 gas in a balloon was bubbled into the mixture under stirring for 30 seconds through a needle, which was then lifted up out of the solution and was kept in the vial. The mixture was placed under a 20 W blue LED light source and stirred at ambient temperature (15—20 °C). Upon completion of the reaction as monitored by TLC, the vial was opened to air and the volatile materials were removed using a rotary evaporator under reduced pressure. The crude residue was dissolved in 2 mL MeOH/Et_2O (1/4), $TMSCHN_2$ (0.3 mL, 0.6 mmol, 2 M in hexanes) was added dropwisely. The mixture was stirred at ambient temperature until the completion of the methylation reaction. All the volatile materials were removed using a rotary evaporator under reduced pressure, and the product was purified by flash chromatography on silica gel using ethyl acetate and petroleum ether as eluents.

Methyl (2-methoxy-2-oxo-1,1-diphenylethyl)-D-valinate (**5a**).

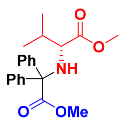

The reaction was performed following the Typical Experimental Procedure with methyl D-valinate hydrochloride (33.5 mg, 0.2 mmol) as starting amino acid ester. Upon completion, the crude product was purified by chromatography on silica gel (eluted with petroleum ether/EtOAc = 100/1) to give the desired product (52.6 mg, 74% yield, 99% ee) as colorless oil.

R_f = 0.31 (hexanes : ethyl acetate = 20:1). ^{1}H NMR (400 MHz, $CDCl_3$): δ 7.44 (d, *J* = 7.1 Hz, 2H), 7.42 – 7.37 (m, 2H), 7.32 – 7.25 (m, 6H), 3.68 (s, 3H), 3.36 (s, 3H), 3.12 (dd, *J* = 10.1, 5.3 Hz, 1H), 3.04 (d, *J* = 10.1 Hz, 1H), 1.92 (dq, *J* = 13.5, 6.8 Hz, 1H), 0.92 (d, *J* = 6.9 Hz, 3H), 0.89 (d, *J* = 6.8 Hz, 3H) ppm; ^{13}C{^{1}H} NMR (100 MHz, $CDCl_3$): δ 175.03, 173.92, 142.18, 141.73, 129.16, 128.76, 127.96, 127.89, 127.63, 127.48, 72.56, 61.59, 52.57, 51.21, 33.45, 19.29, 18.67 ppm. IR (thin film): 3947, 3566, 3054, 2986, 1732, 1265, 737, 704 cm^{-1}; HRMS calcd for $C_{21}H_{26}NO_4^+$ 356.1862, observed 356.1857 [M+H]$^+$. The ee was determined by HPLC with a Daicel Chiralpak OD-H column (0.5% isopropanol in hexanes, 0.3 mL/min, 254 nm, t_r (D/R, major) = 28.1 min, t_r (L/S) = 29.7 min). The racemate compounds for HPLC reference were prepared by mixing of the two enantiomer products.

***tert*-butyl (2-methoxy-2-oxo-1,1-diphenylethyl)-L-leucinate (5b)**.

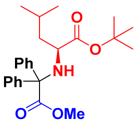

The reaction was performed following the Typical Experimental Procedure with *tert*-butyl L-leucinate hydrochloride (44.7 mg, 0.2 mmol) as starting amino acid ester. Upon completion, the crude product was purified by chromatography on silica gel (eluted with petroleum ether/EtOAc =100/1) to give the desired product (58.4 mg, 71% yield, 99% ee) as colorless oil.

R_f = 0.44 (hexanes : ethyl acetate = 20:1). ^{1}H NMR (400 MHz, $CDCl_3$): δ 7.48 – 7.38 (m, 4H), 7.31 – 7.23 (m, 6H), 3.69 (s, 3H), 3.21 – 3.01 (m, 2H), 1.72 (dq, *J* = 13.2, 6.7 Hz, 1H), 1.47 – 1.38 (m, 2H), 1.31 (s, 9H), 0.80 (d, *J* = 6.6 Hz, 3H), 0.78 (d, *J* = 6.6 Hz, 3H) ppm; ^{13}C{^{1}H} NMR (100 MHz, $CDCl_3$): δ 175.27, 174.02, 142.76, 141.82, 129.01, 128.41, 127.97, 127.88, 127.49, 127.34, 80.54, 72.52, 55.51, 52.58, 45.56, 28.09, 24.73, 23.18, 22.43 ppm. IR (thin film): 3566, 3054, 2984, 1731, 1265, 1153, 738, 704 cm^{-1}; HRMS calcd for $C_{25}H_{34}NO_4^+$ 412.2488, observed 412.2480 [M+H]$^+$. The ee was determined by HPLC with a Daicel Chiralpak OD-H column (0.5% isopropanol in hexanes, 0.3 mL/min, 254 nm, t_r (L/S, major) = 17.2 min, t_r (D/R) = 17.9 min). The racemate compounds for HPLC reference were prepared by mixing of the two enantiomer products.

Benzyl (2-methoxy-2-oxo-1,1-diphenylethyl)-D-phenylalaninate (5c).

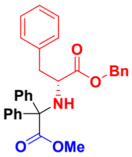

The reaction was performed following the Typical Experimental Procedure with benzyl D-phenylalaninate hydrochloride (58.4 mg, 0.2 mmol) as starting amino acid ester. Upon completion, the crude product was purified by chromatography on silica gel (eluted with petroleum ether/EtOAc = 50/1) to give the desired product (64.3 mg, 67% yield, 99% ee) as colorless oil.

R_f = 0.70 (hexanes : ethyl acetate = 10:1). ^{1}H NMR (400 MHz, $CDCl_3$): δ 7.36 – 7.31 (m, 2H), 7.30 – 7.19 (m, 14H), 7.06 (ddd, *J* = 18.2, 6.7, 2.5 Hz, 4H), 4.68 (d, *J* = 12.2 Hz, 1H), 4.55 (d, *J* = 12.2 Hz, 1H), 3.63 (s, 3H), 3.55 (dt, *J* = 9.7, 6.9 Hz,

1H), 3.18 (d, J = 9.7 Hz, 1H), 2.89 (qd, J = 13.3, 6.9 Hz, 2H) ppm; $^{13}C\{^1H\}$ NMR (100 MHz, $CDCl_3$): δ 174.63, 173.80, 141.50, 141.42, 137.38, 135.53, 129.83, 128.88, 128.83, 128.48, 128.43, 128.38, 128.23, 128.10, 127.91, 127.63, 127.54, 126.70, 72.68, 66.45, 58.45, 52.69, 41.94 ppm. IR (thin film): 3648, 3566, 3054, 1732, 1455, 1265, 738, 704 cm^{-1}; HRMS calcd for $C_{31}H_{30}NO_4^+$ 480.2175, observed 480.2174 $[M+H]^+$. The ee was determined by HPLC with a Daicel Chiralpak OD-H column (5% isopropanol in hexanes, 0.5 mL/min, 254 nm, t_r (D/R, major) = 19.6 min, t_r (L/S) = 29.8 min). The racemate compounds for HPLC reference were prepared by mixing of the two enantiomer products.

Ethyl (2-methoxy-2-oxo-1,1-diphenylethyl)-L-tyrosinate (5d).

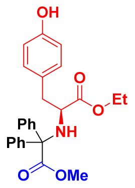

The reaction was performed following the Typical Experimental Procedure with ethyl L-tyrosinate hydrochloride (49.1 mg, 0.2 mmol) as starting amino acid ester. Upon completion, the crude product was purified by chromatography on silica gel (eluted with petroleum ether/EtOAc = 100/1) to give the desired product (69.4 mg, 80% yield, 97% ee) as colorless oil.

R_f = 0.33 (hexanes : ethyl acetate = 20:1). ^1H NMR (400 MHz, $CDCl_3$): δ 7.31 (d, J = 7.1 Hz, 2H), 7.28 – 7.14 (m, 8H), 7.08 (d, J = 8.6 Hz, 2H), 6.82 (d, J = 8.6 Hz, 2H), 4.78 (s, 1H), 4.12 (q, J = 7.1 Hz, 2H), 3.80 (s, 3H), 3.39 (t, J = 6.8 Hz, 1H), 3.02 – 2.79 (m, 2H), 2.13 (s, 1H), 1.19 (t, J = 7.1 Hz, 3H) ppm; $^{13}C\{^1H\}$ NMR (100 MHz, $CDCl_3$): δ 175.01 (X2), 158.47, 144.43, 142.80, 130.60, 129.72, 128.60, 128.51, 127.59, 127.42, 127.24, 127.15, 113.74, 65.54, 60.84, 60.72, 55.40, 39.36, 14.41 ppm. IR (thin film): 3054, 2985, 1730, 1513, 1265, 1248, 737, 703 cm^{-1}; HRMS calcd for $C_{26}H_{28}NO_5^+$ 434.1967, observed 434.1971 $[M+H]^+$. The ee was determined by HPLC with a Daicel Chiralpak OD-H column (5% isopropanol in hexanes, 0.5 mL/min, 254 nm, t_r (L/S, major) = 13.2 min, t_r (D/R) = 13.9 min). The racemate compounds for HPLC reference were prepared by mixing of the two enantiomer products.

Debenzylation Procedure to Synthesize Free Amino Acid

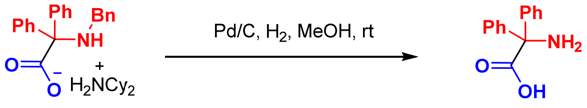

Compound **4aa** (2.0 g, 4.0 mmol), catalyst Pd/C (848 mg, 0.2 eq, 10% Pd, wet), MeOH (20 mL) and a magnetic stirring bar were charged into an 50 mL Schlenk flask under nitrogen. The flask was sealed with a septum. The nitrogen was replaced with hydrogen gas using vacuum and a hydrogen gas balloon. The hydrogen gas balloon was kept in the flask and the mixture was stirred at ambient temperature (25—30 °C). Upon completion of the reaction as monitored by TLC (~ 24 h), the flask was opened and the solvent was removed using rotary evaporator under vacuum. The crude product was dissolved in aqueous NaOH solution (10 mL, 1 M). Pd/C and Cy_2NH were removed by filtration through a Celite column, and the Celite cake was washed with aqueous NaOH solution (3 X 3 mL, 1 M). The filtrate was collected and the pH was titrated to 6 using HCl aqueous solution (2 M). Most of the solvent (~ 25 mL) was removed using rotary evaporator under vacuum and the product was precipitated. The solid was collected by filtration and dried under vacuum to give the final product **6aa** (0.81 g, 89%).

This is a known compound, and its characterization data is consistent with the reported data.[1] ^{1}H NMR (400 MHz, D_2O, 1% DCl as additive for better solubility): δ 6.79 – 6.68 (m, 6H), 6.66 – 6.55 (m, 4H) ppm; $^{13}C\{^{1}H\}$ NMR (100 MHz, D_2O, 1% DCl as additive for better solubility): δ 170.90, 135.02, 129.37, 128.65, 127.09, 68.63 ppm.

UV-visible Absorption Analysis

The UV-visible absorption of the substrates *N*-benzyl-1,1-diphenylmethanimine (**1a**), Cy_2NMe, and of the reaction mixture without catalyst was determined at a diluted solution (0.01 M) in MeCN. The UV/vis spectrum of the reaction mixture with catalyst (**Ir-7**) was measured after 30 min of irradiation with blue LED. The results are summarized in Supplementary Figure 48.

The photoredox catalyst (**Ir-7**) is the only species that absorbs light at 400 nm according to the UV/vis spectra.

Stern-Volmer Luminescence Quenching Analysis

A 2 X 10^{-5} M solution of photoredox catalyst (**Ir-7**) and variable concentrations of N-benzyl-1,1-diphenylmethanimine (**1a**) or Cy_2NMe in dry MeCN were used for the Stern-Volmer luminescence quenching studies at room temperature under an argon atmosphere. The samples were prepared in 3 mL quartz cuvettes, equipped with PTFE stoppers and sealed with parafilm inside an glovebox. The solutions were irradiated at 400 nm and the luminescence was measured at 574 nm. The ratio of I_0/I was plotted as a function of the quencher concentration (I_0 = emission intensity of the photocatalyst; I = observed emission intensity of the photocatalyst with quencher). The results are summarized in Supplementary Figure 49.

The results prove that the photoredox catalyst is only quenched by Cy_2NMe. This indicates that Cy_2NMe acts as electron donor and reduces the excited catalyst $[Ir]^{3+}$* in the catalytic cycle.

Chemical Synthesis of Unnatural GLP1 Fragment

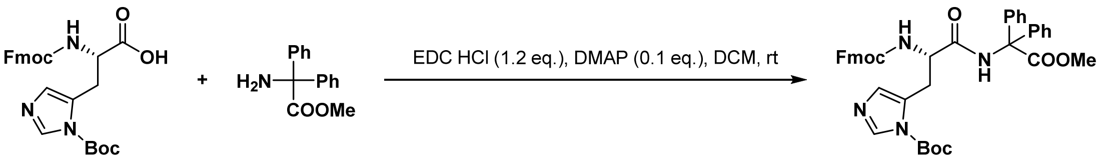

In addition to incorporation of unnatural amino acids into proteins using genetic code expansion techniques, chemical synthesis of peptides using unnatural amino acids is an alternative pathway. Thus, the chemical synthesis of unnatural GLP1 fragment was tested. Fmoc-His(Boc)-OH (47.8 mg, 0.1 mmol, 1 eq.), N-(3-dimethylaminopropyl)-N'-ethylcarbodiimide hydrochloride (23 mg, 0.12 mmol, 1.2 eq.), 4-dimethylaminopyridine (1.2 mg, 0.01 mmol, 0.1 eq.), dichloromethane (0.4 mL) and a magnetic stirring bar were charged into an 5 mL flask under nitrogen. The flask was sealed with a septum. After stirring for 5 minutes at room temperature, dichloromethane (0.2 mL) solution of diphenylglycine methyl ester (28.9 mg, 0.12 mmol, 1.2 eq.) was added. The mixture was stirred at ambient temperature (25—30 °C). Upon completion of the reaction as monitored by TLC (~ 10 h), the flask was opened and the solvent was removed using rotary evaporator under vacuum. The residue was purified by flash chromatography on silica gel using ethyl acetate and petroleum ether (50/1) as eluents to give the desired product (57.5 mg, 82% yield).

m.p. 150.7-151.5 °C. R_f = 0.55 (hexanes : ethyl acetate = 1:1). ^{1}H NMR (400 MHz, $CDCl_3$): δ 8.55 (s, 1H), 8.02 (s, 1H), 7.76 (d, *J* = 7.5 Hz, 2H), 7.63 (d, *J* = 7.3 Hz, 2H), 7.40 (t, *J* = 7.5 Hz, 2H), 7.35 – 7.18 (m, 12H), 7.08 (s, 1H), 6.80 (d, *J* = 6.6 Hz, 1H), 4.63 (q, *J* = 6.0 Hz, 1H), 4.50 – 4.33 (m, 2H), 4.27 (t, *J* = 6.9 Hz, 1H), 3.71 (s, 3H), 3.13 (dd, *J* = 14.9, 4.0 Hz, 1H), 2.89 (dd, *J* = 14.9, 6.1 Hz, 1H), 1.63 (s, 9H) ppm; ^{13}C NMR (100 MHz, $CDCl_3$): δ 172.09, 169.50, 156.48, 147.02, 144.04, 143.96, 141.40, 139.23, 138.97, 138.83, 136.79, 128.53, 128.31, 127.93, 127.89, 127.83, 127.24, 125.43, 125.39, 120.10, 115.08, 85.90, 69.78, 67.54, 54.92, 53.49, 47.25, 29.91, 28.03 ppm. IR (thin film): 3054, 2986, 2305, 1422, 1265, 896, 737, 705 cm^{-1}; HRMS calcd for $C_{41}H_{41}N_4O_7^+$ 701.2975, observed 701.2971 $[M+H]^+$.

Supplementary Figure 1. Reactions after completion with different amines. Only Cy_2NMe gives precipitates of products.

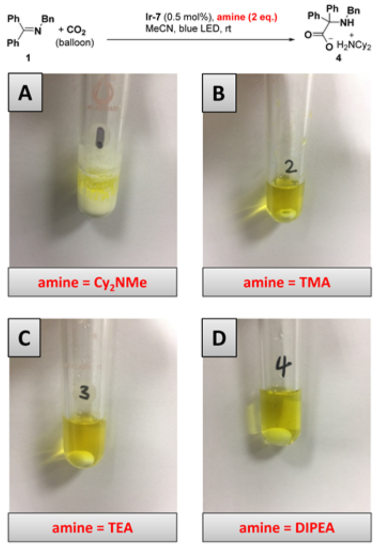

Supplementary Figure 2. Products obtained by filtration from gram scale reactions mediated by either outdoor sunlight or blue LED.

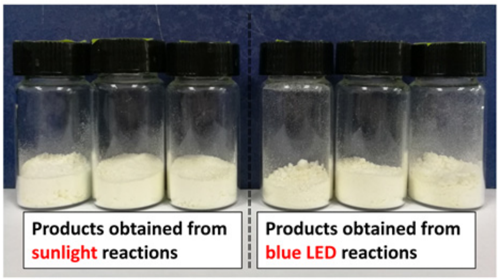

Supplementary Figure 3. Reaction setup of sunlight-promoted CO_2 fixation. A: Before reaction, the mixture is clear solution. **B:** Sunlight-promoted reaction at 30 minutes (CO_2 in balloon). **B':** Zoon in, precipitates start to appear. **C:** Sunlight-promoted reaction at 6 hours. **C':** Zoon in, large amount of precipitates appear. **D:** Pure products obtained by filtration after reaction completion. **Weather information:** Slightly cloudy. ~ 16 °C. March 22, 2018 in Nanjing, China.

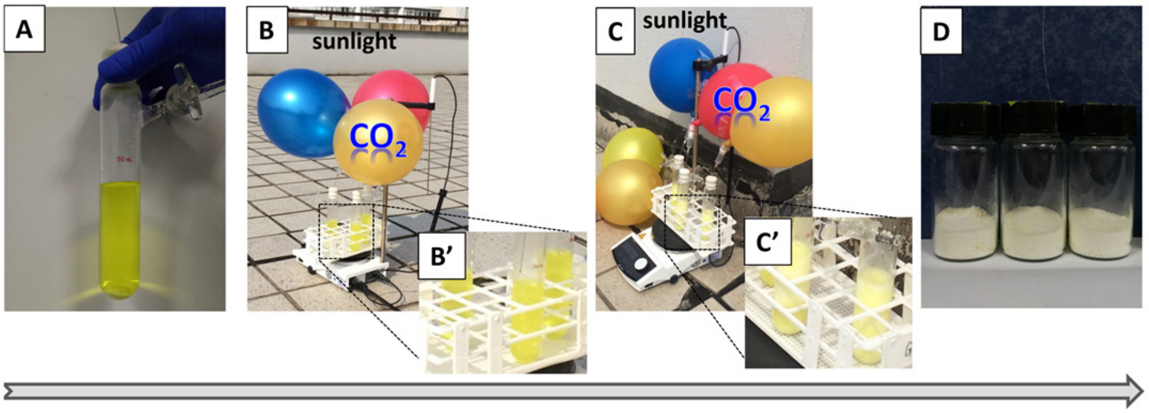

Supplementary Figure 4. 10 gram scale reaction setup of sunlight-promoted CO_2 fixation. A: Before reaction, the mixture is clear solution in a 250 mL Schlenk flask. **B:** Sunlight-promoted reaction at 7 h (CO_2 in balloon). **B':** Zoon in,

product precipitates. **C:** Pure products obtained by filtration after reaction completion. **Weather information:** Sunny. 26 – 29 °C. April 9 and 10, 2018 in Nanjing, China.

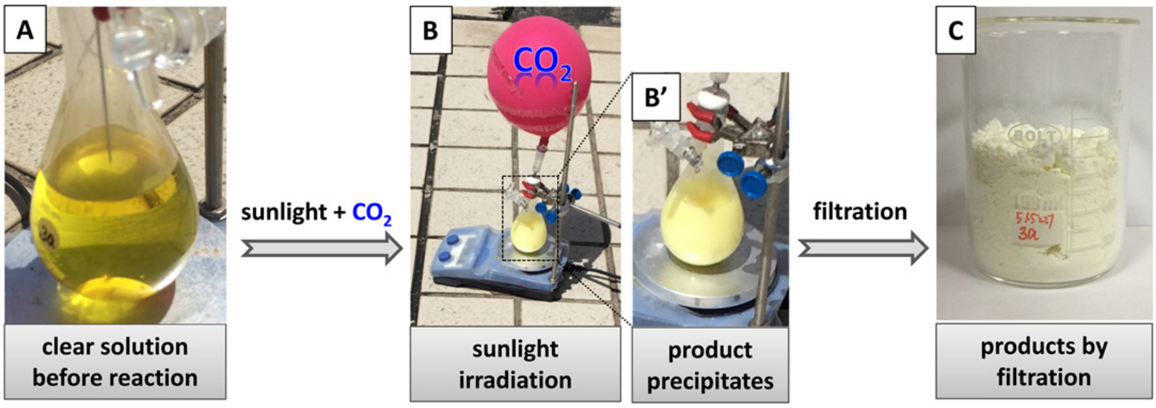

Supplementary Figure 5. ^{1}H and $^{13}C\{^{1}H\}$ NMR spectra of compound **3a** in $CDCl_3$

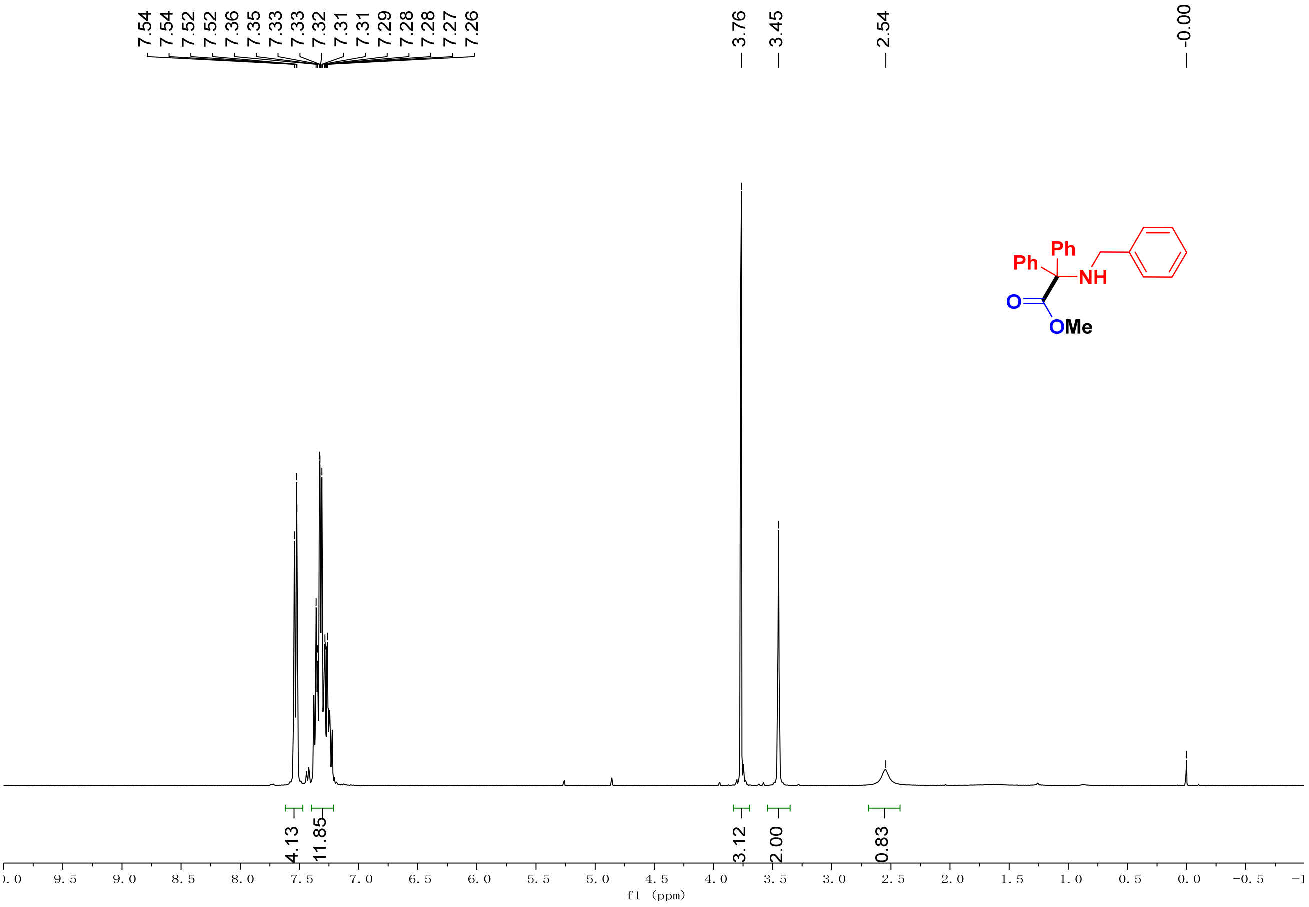

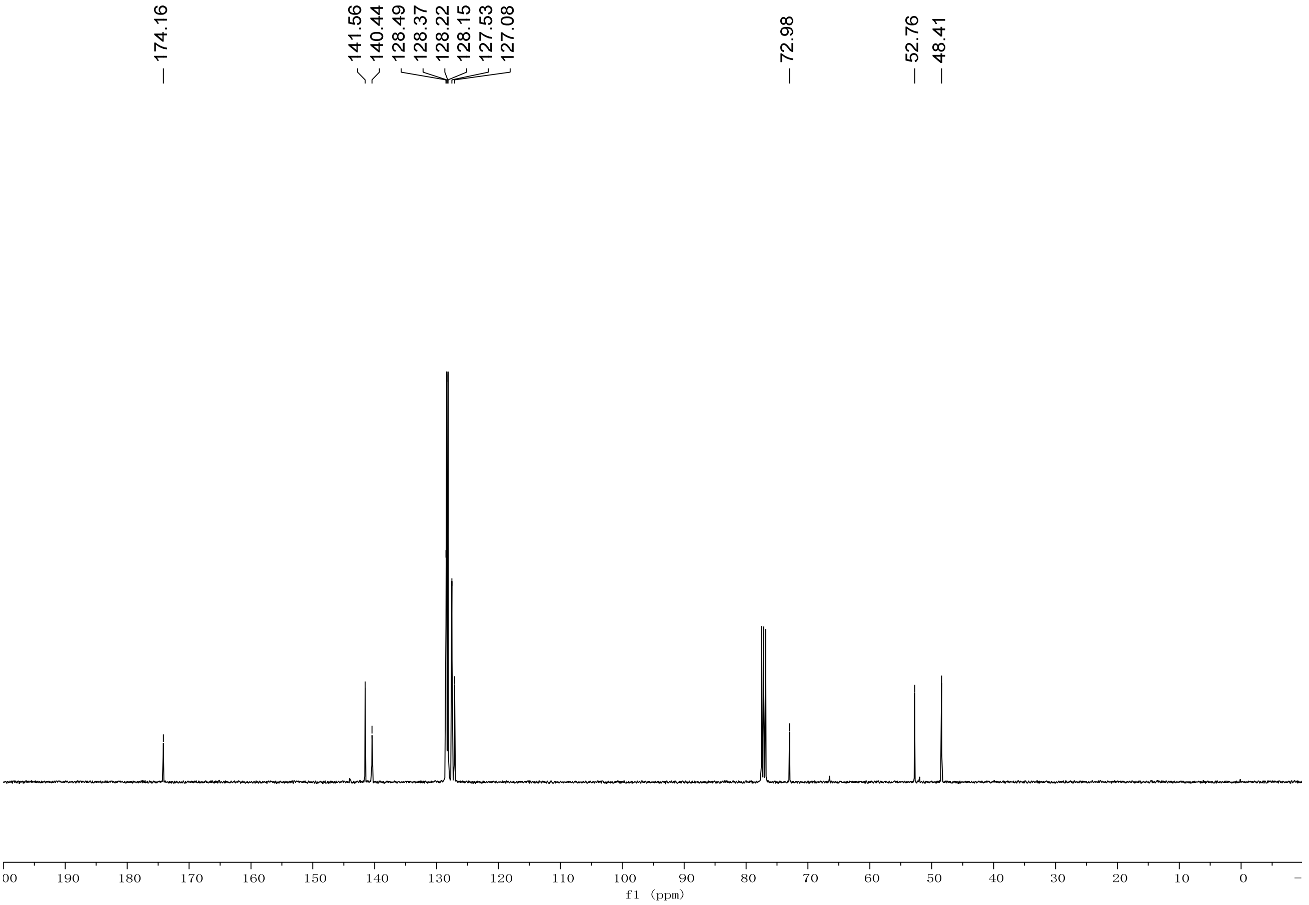

Supplementary Figure 6. ^{1}H, $^{13}C\{^{1}H\}$ and ^{19}F NMR spectra of compound **3b** in $CDCl_3$

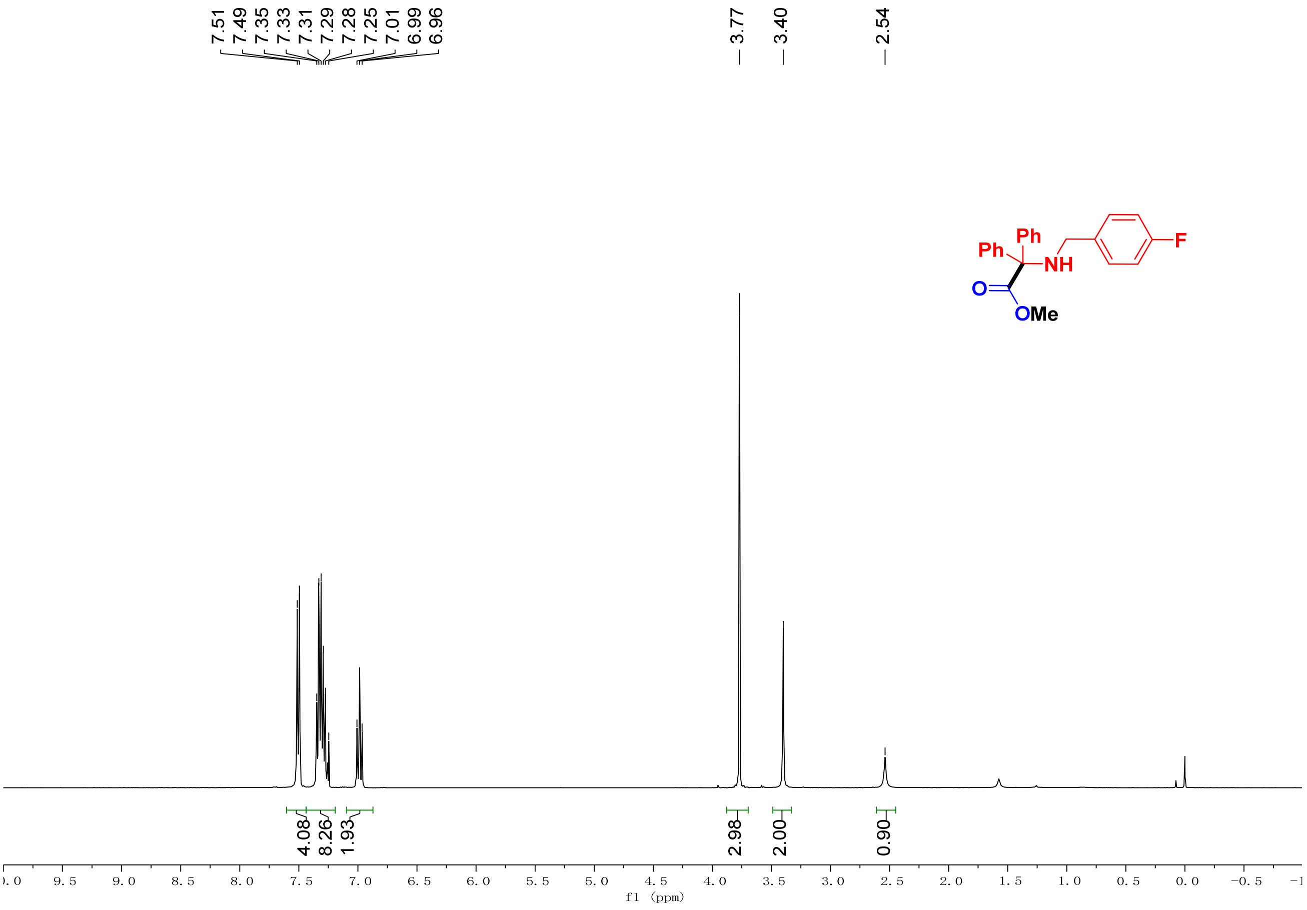

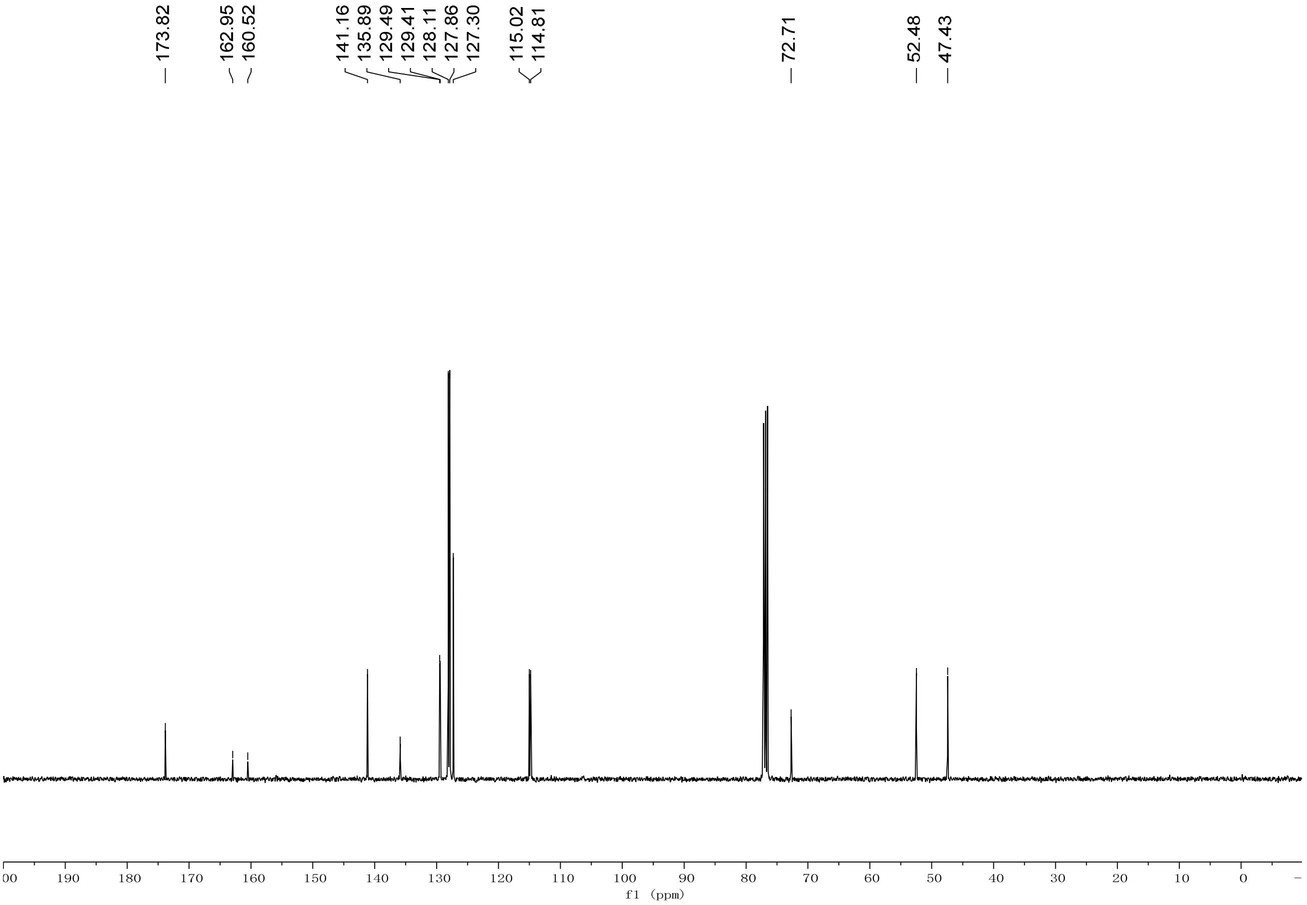

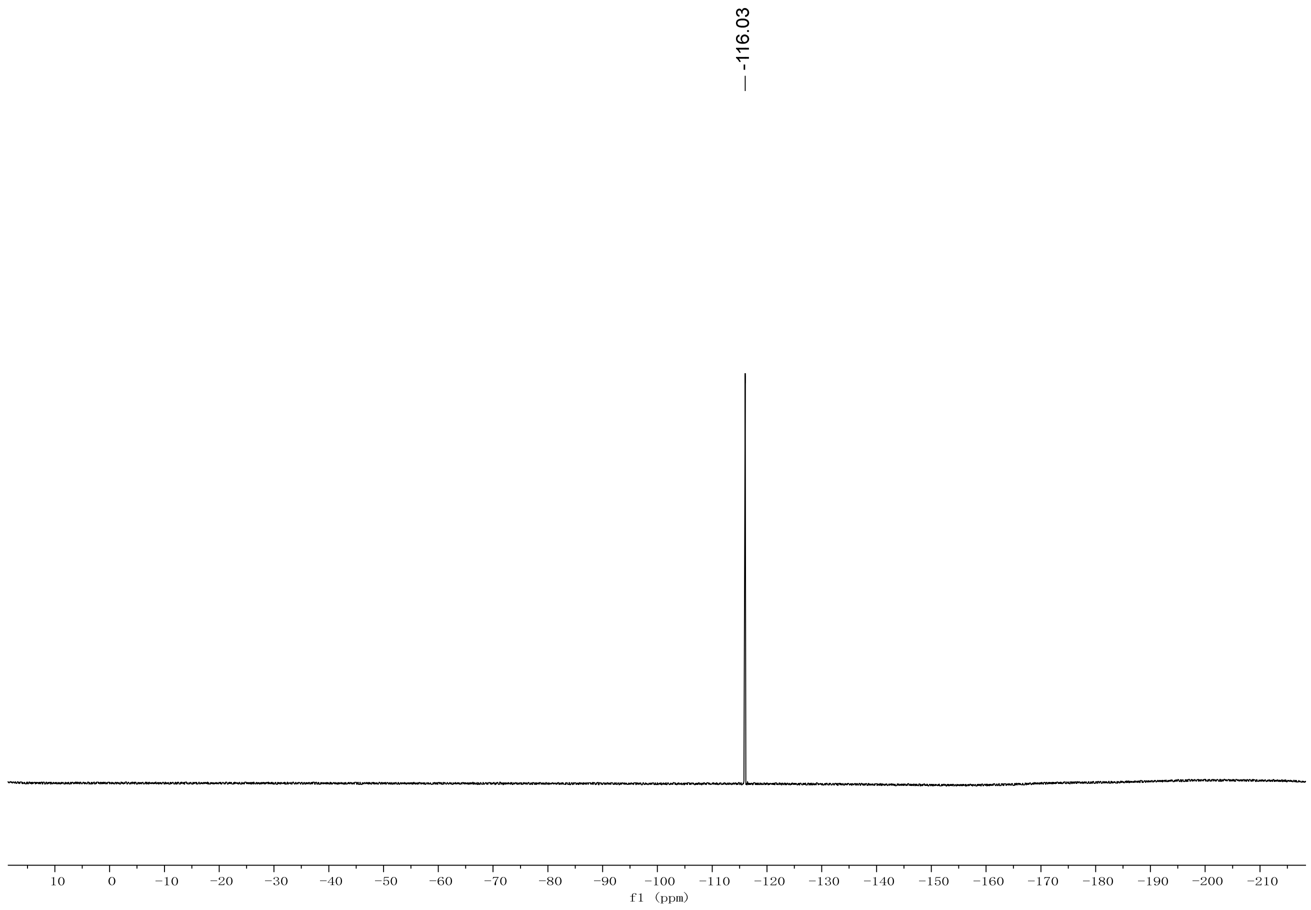
-116.03
10 0 −10 −20 −30 −40 −50 −60 −70 −80 −90 −100 −110 −120 −130 −140 −150 −160 −170 −180 −190 −200 −210
f1 (ppm)

Supplementary Figure 7. ^{1}H and ^{13}C{^{1}H} NMR spectra of compound **3c** in $CDCl_3$

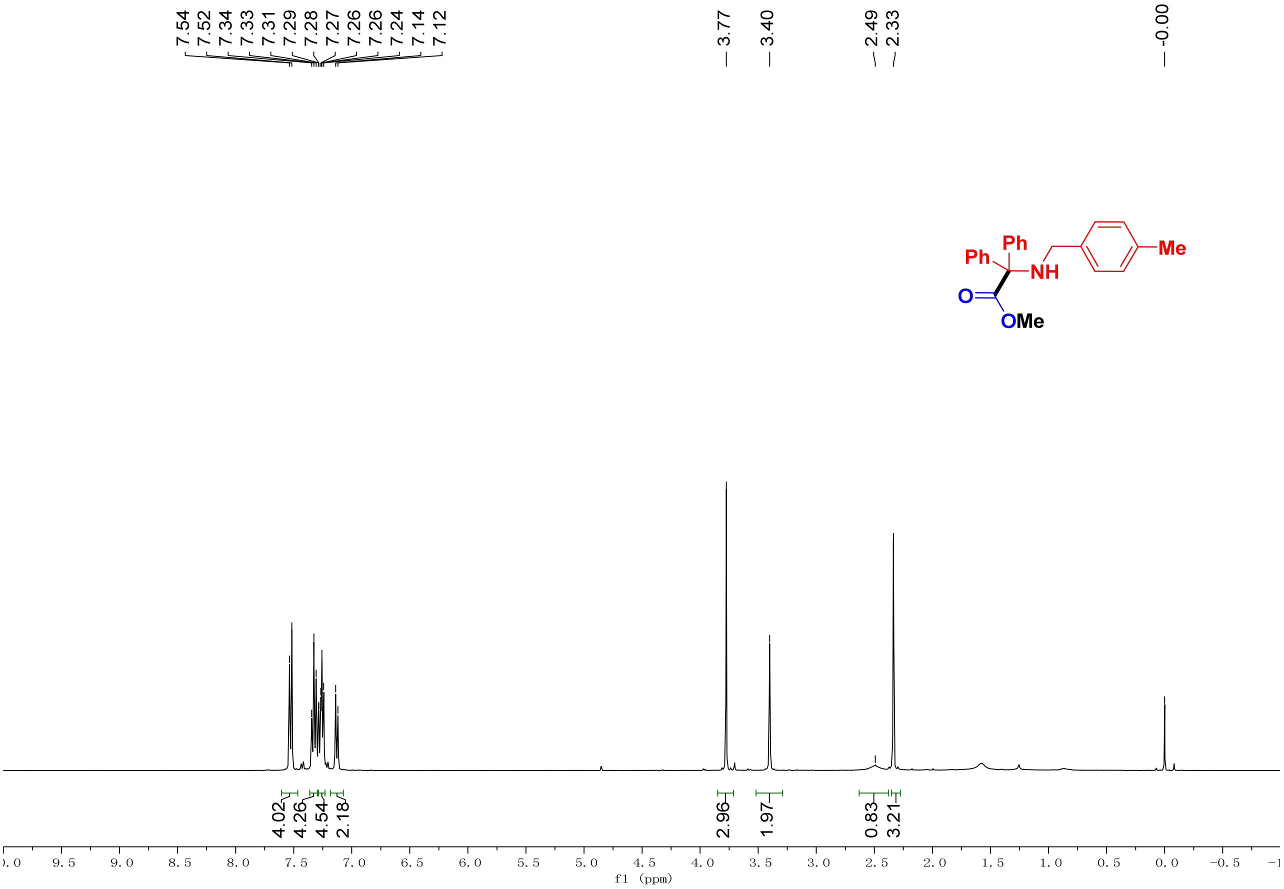

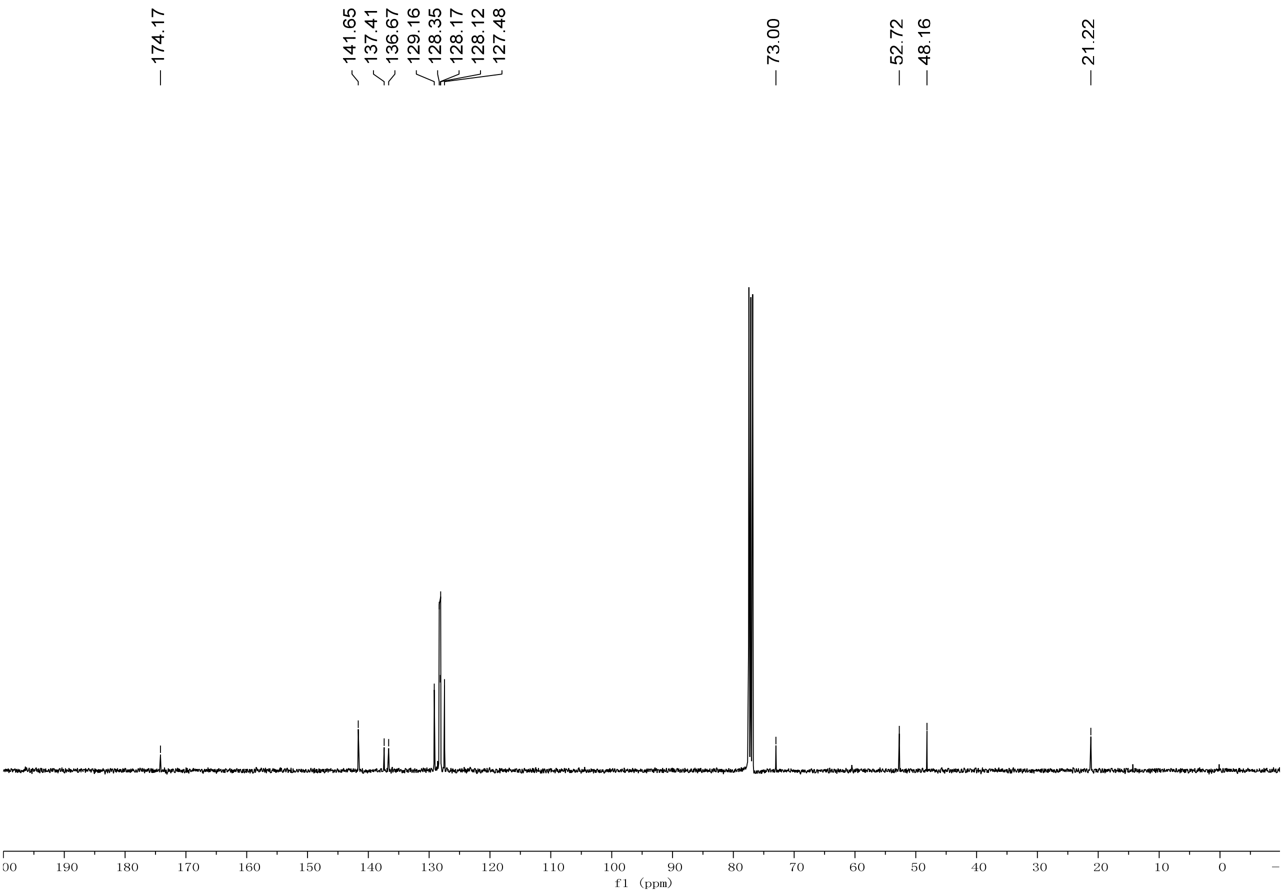

Supplementary Figure 8. ^{1}H and $^{13}C\{^{1}H\}$ NMR spectra of compound **3d** in $CDCl_3$

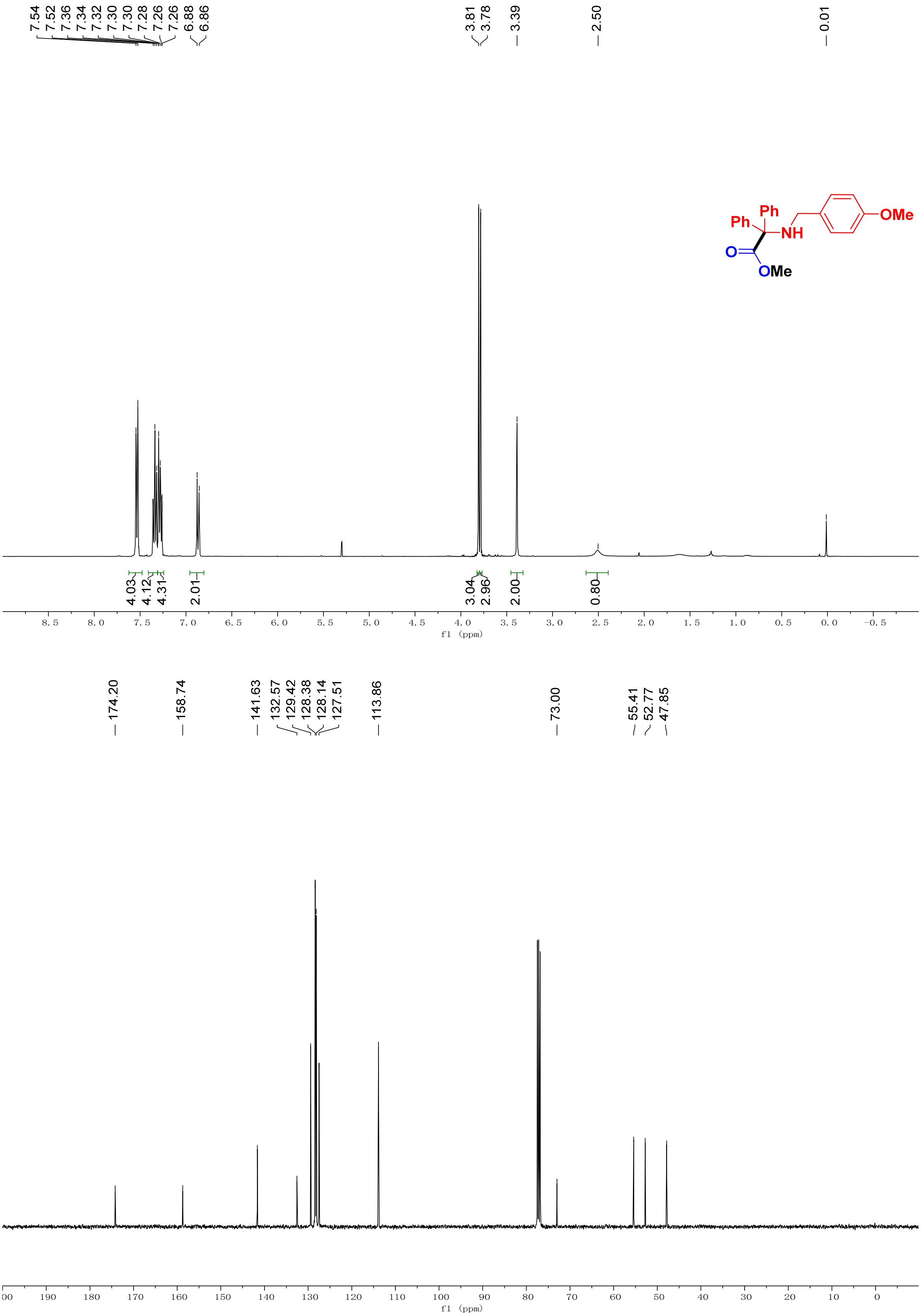

Supplementary Figure 9. ¹H and ¹³C{¹H} NMR spectra of compound **3e** in $CDCl_3$

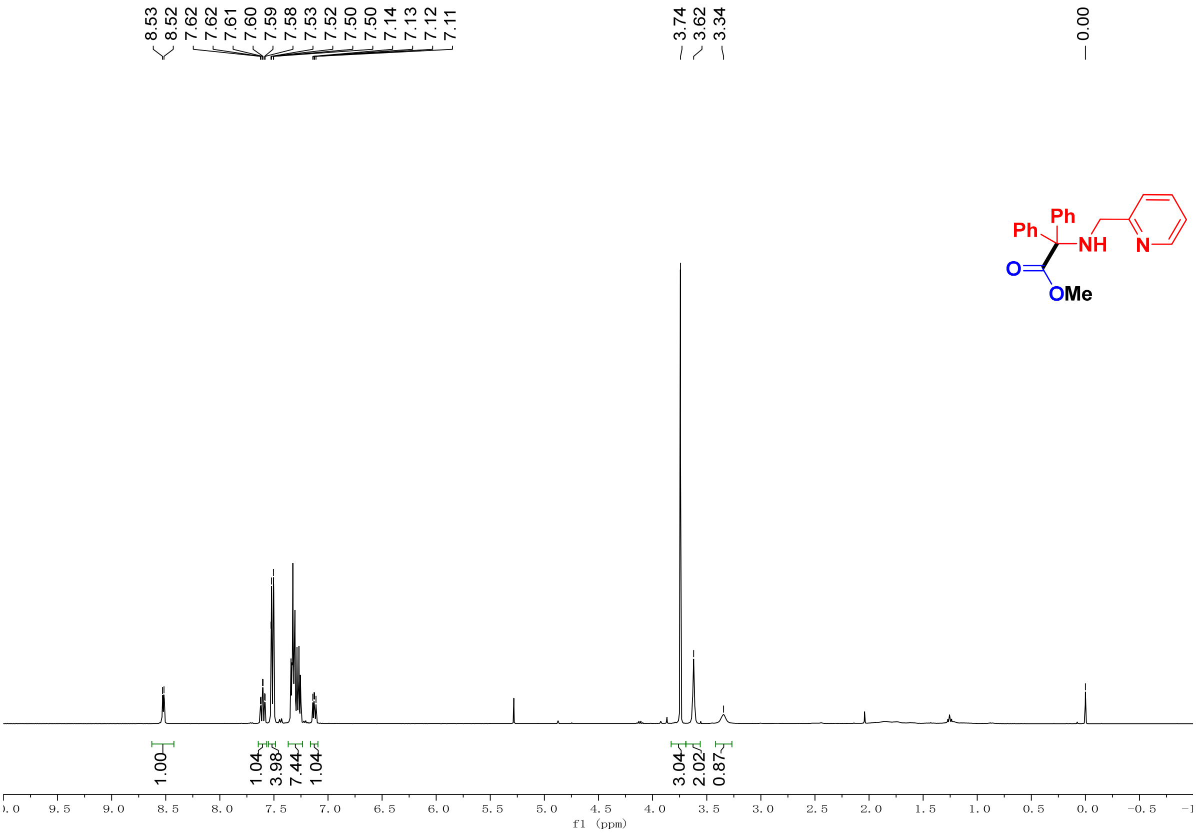

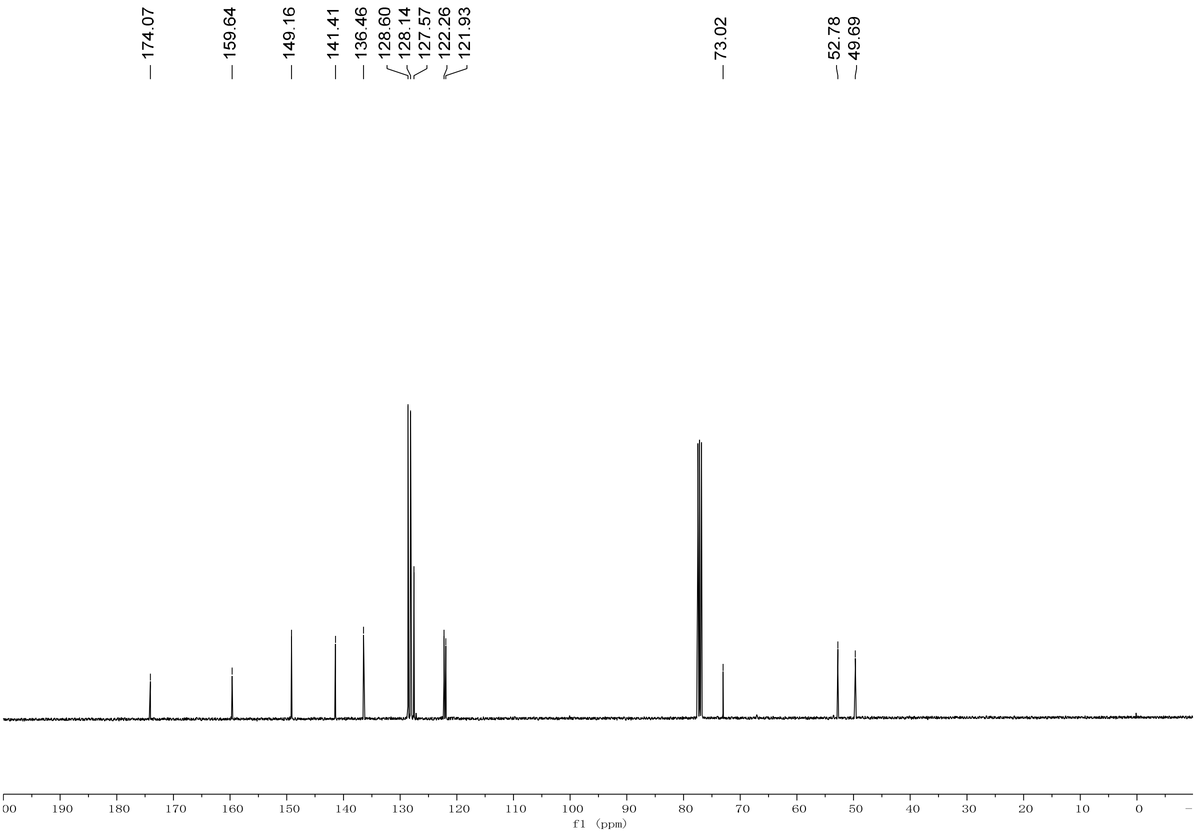

Supplementary Figure 10. ^{1}H and $^{13}C\{^{1}H\}$ NMR spectra of compound **3f** in $CDCl_3$

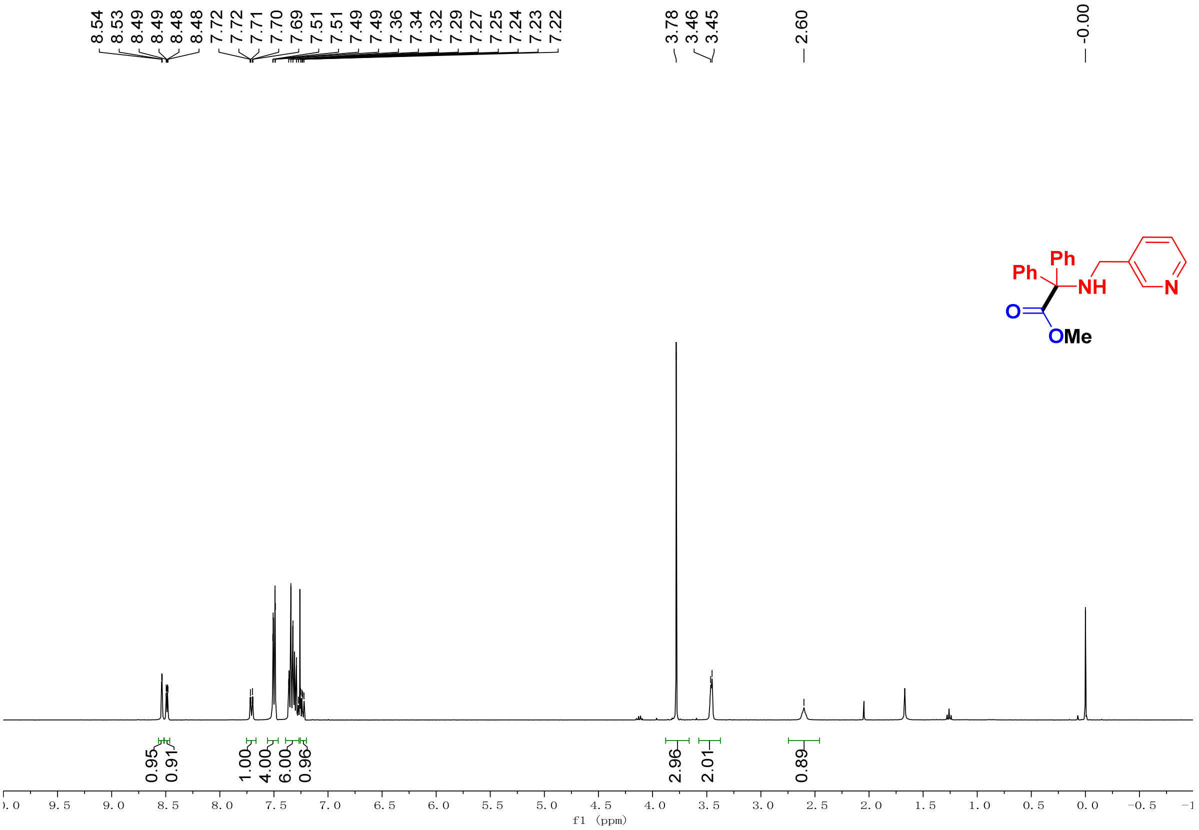

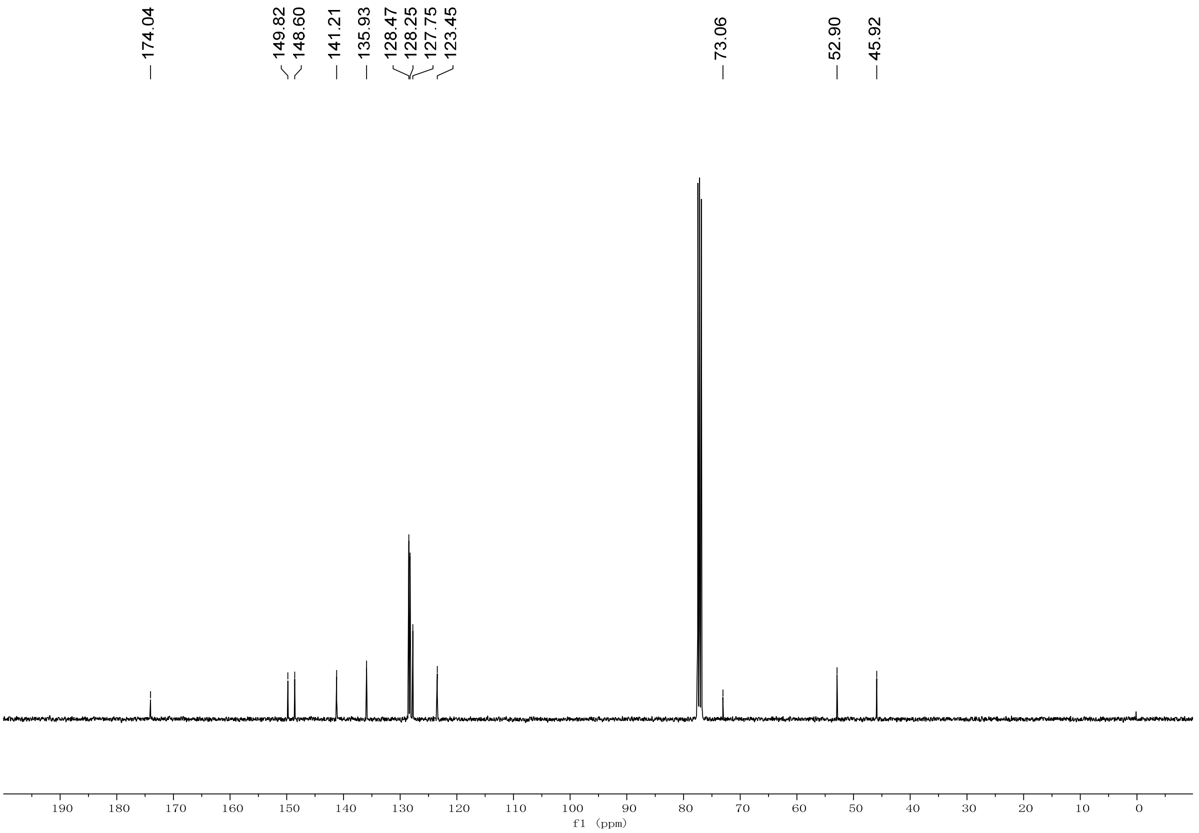

Supplementary Figure 11. ^{1}H and $^{13}C\{^{1}H\}$ NMR spectra of compound **3g** in $CDCl_3$

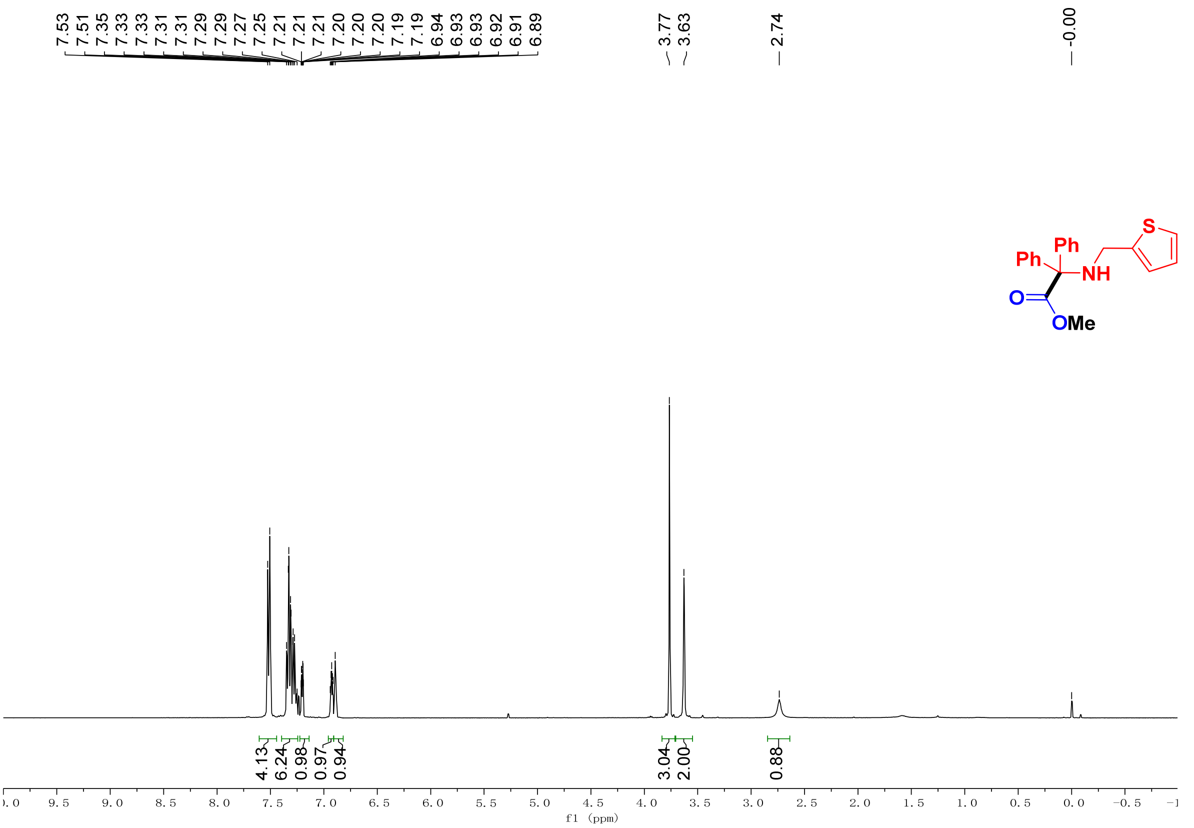

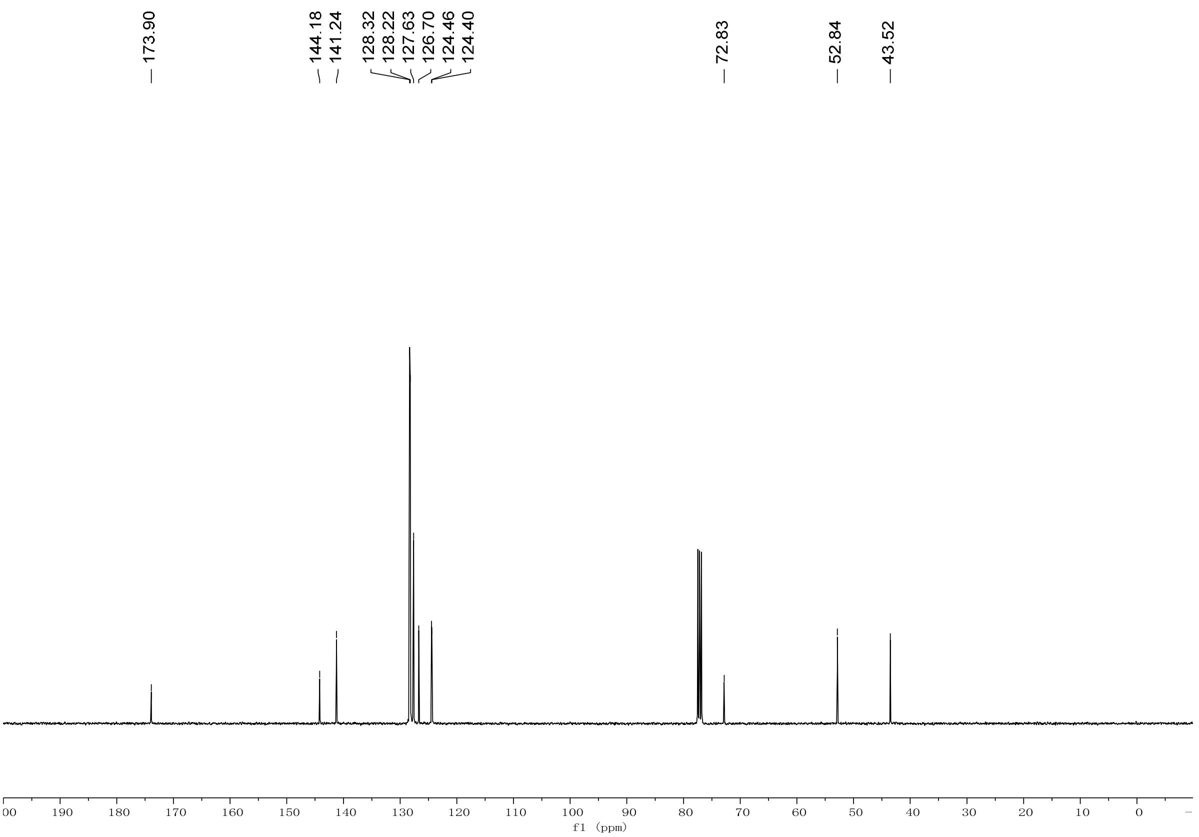

Supplementary Figure 12. ^{1}H and ^{13}C{^{1}H} NMR spectra of compound **3h** in $CDCl_3$

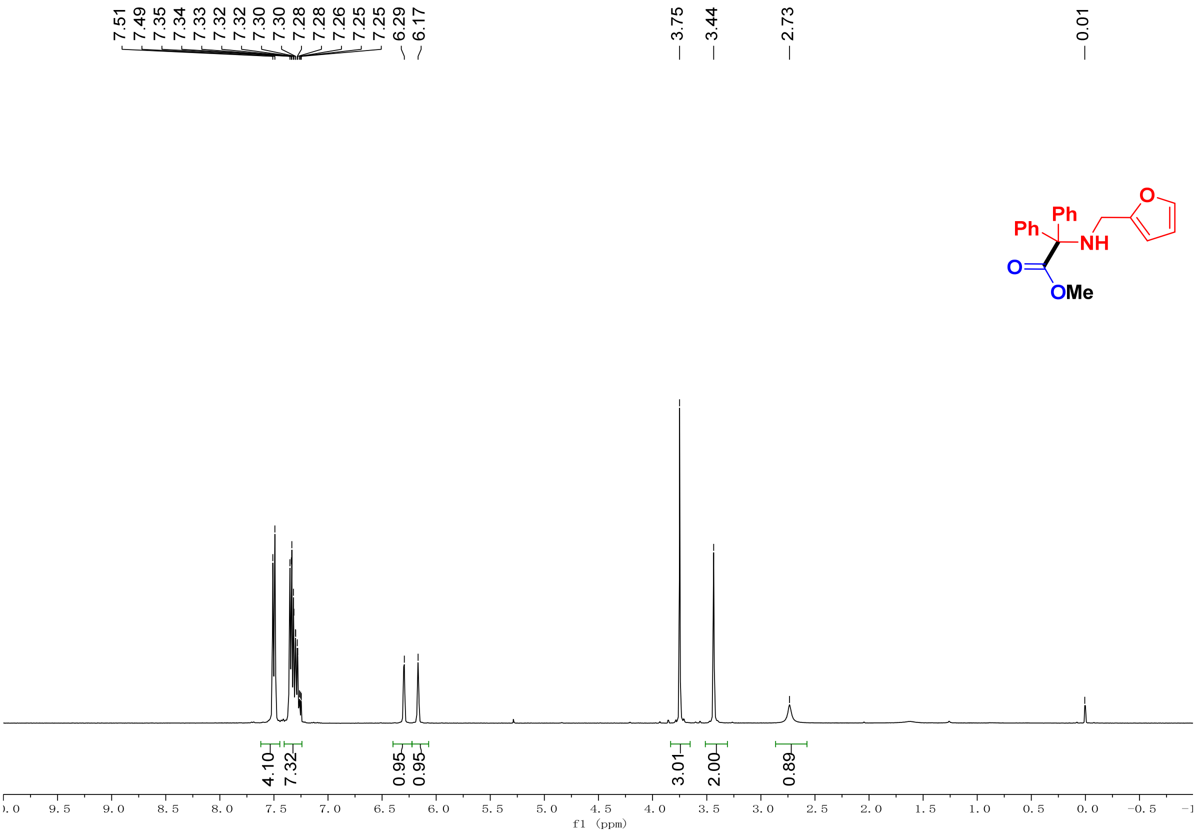

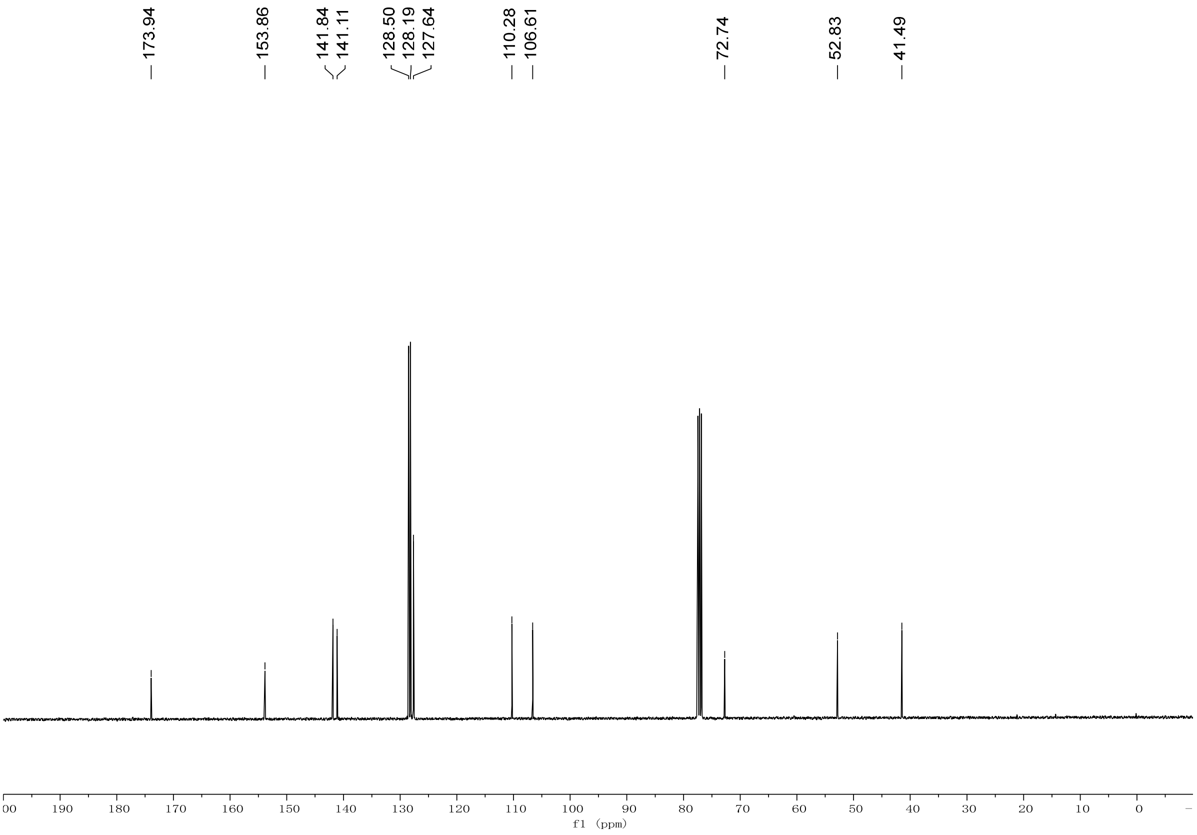

Supplementary Figure 13. ^{1}H and $^{13}C\{^{1}H\}$ NMR spectra of compound **3i** in $CDCl_3$

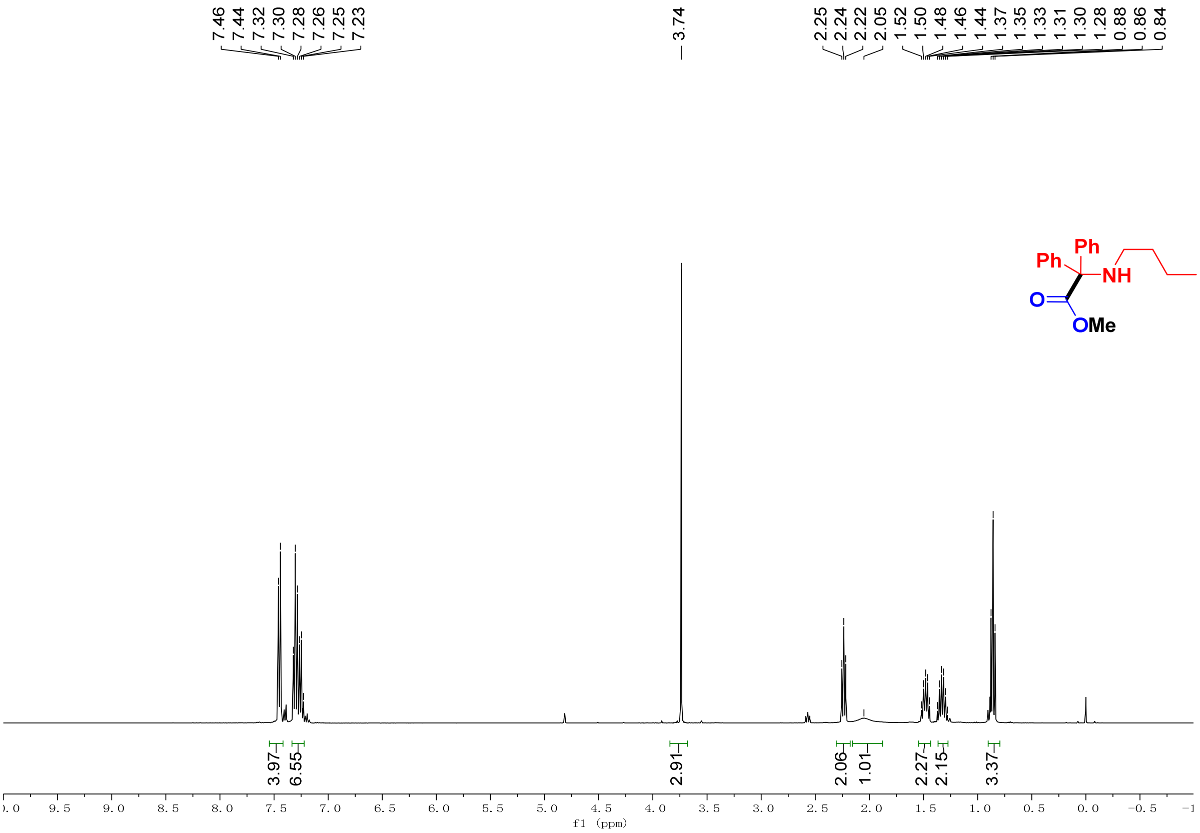

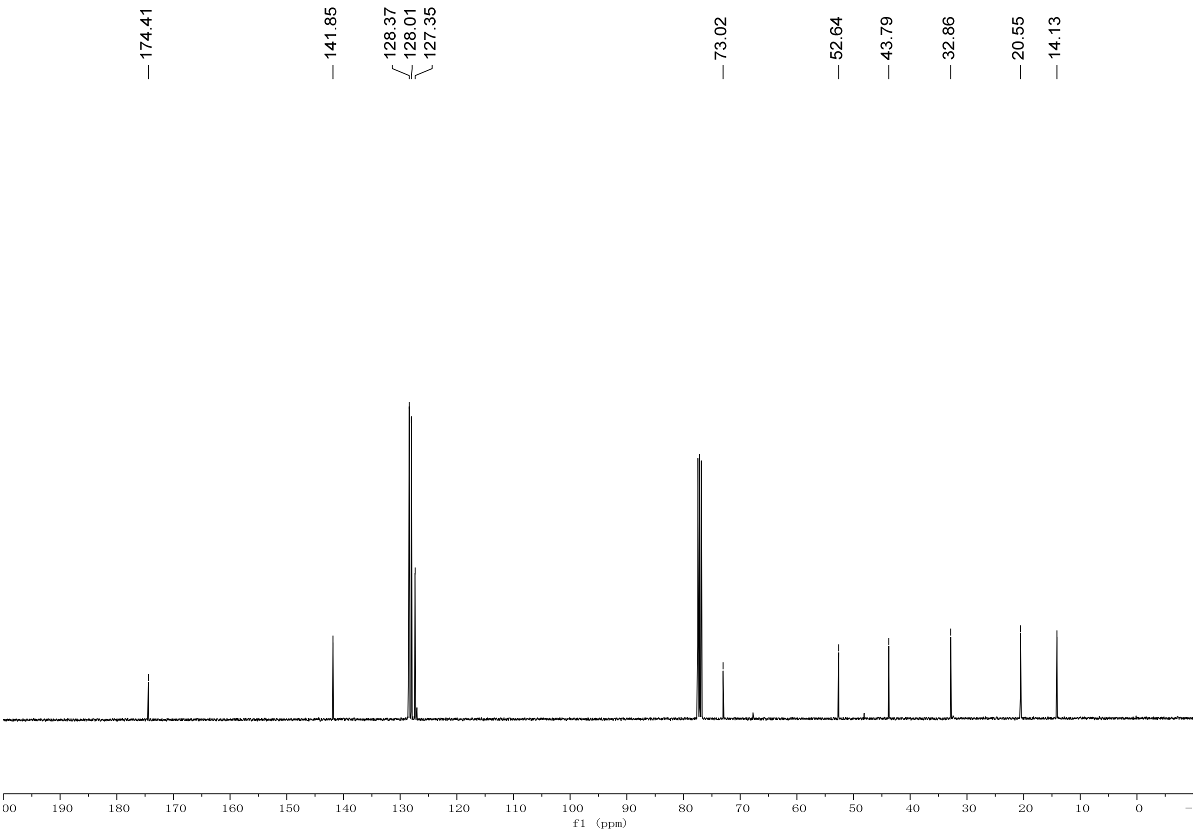

Supplementary Figure 14. ^{1}H and ^{13}C{^{1}H} NMR spectra of compound **3j** in $CDCl_3$

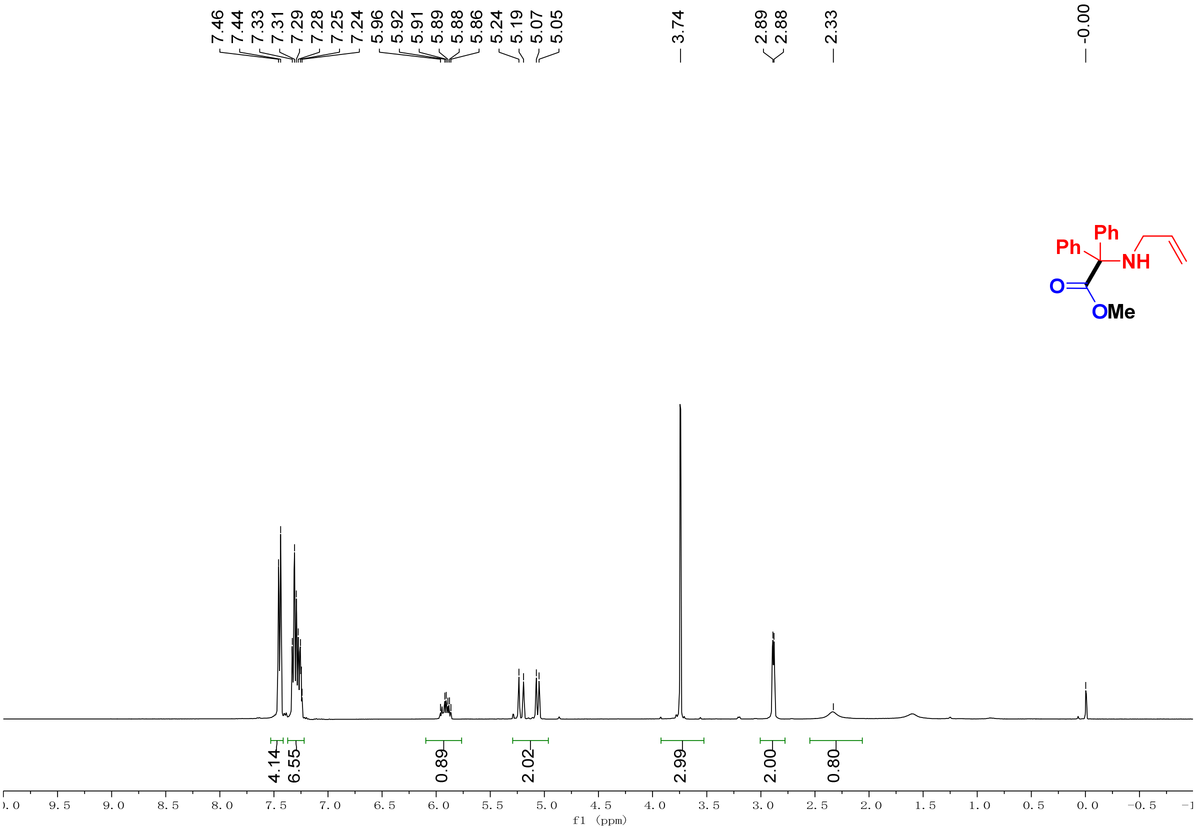

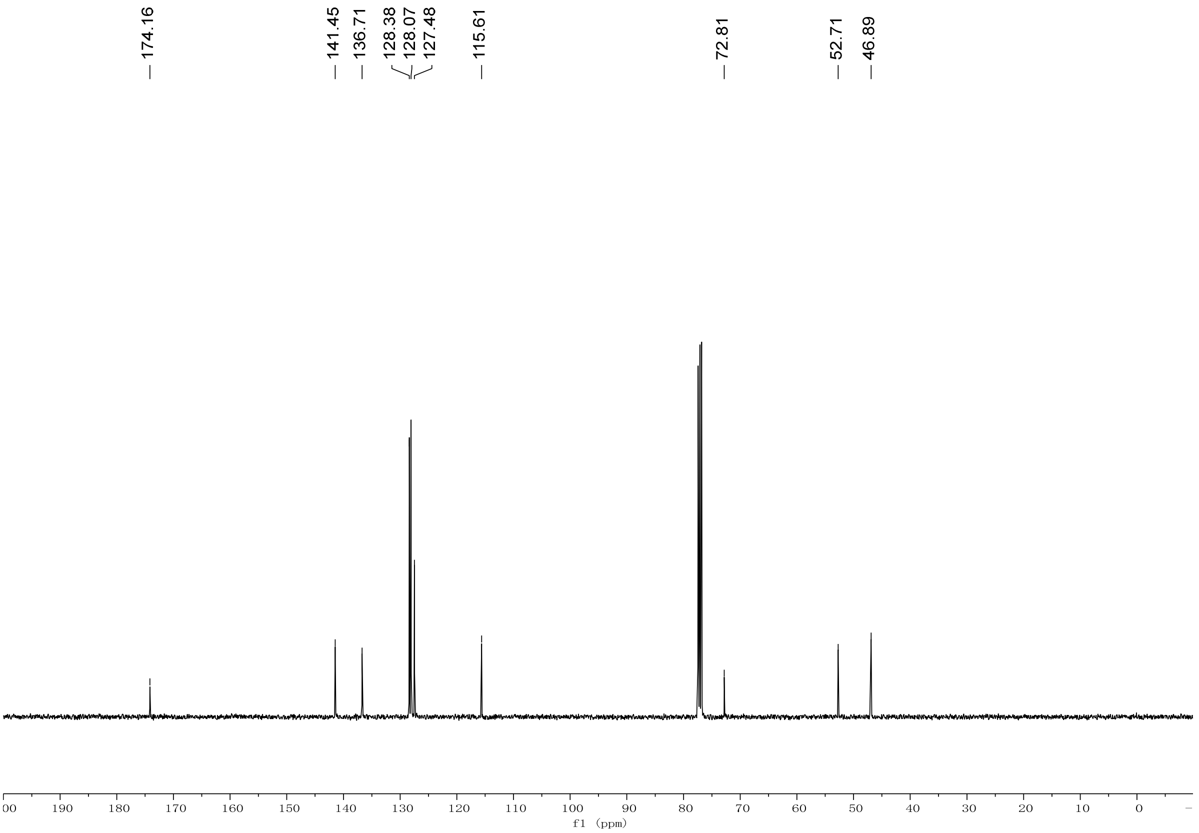

Supplementary Figure 15. ^{1}H and $^{13}C\{^{1}H\}$ NMR spectra of compound **3k** in $CDCl_3$

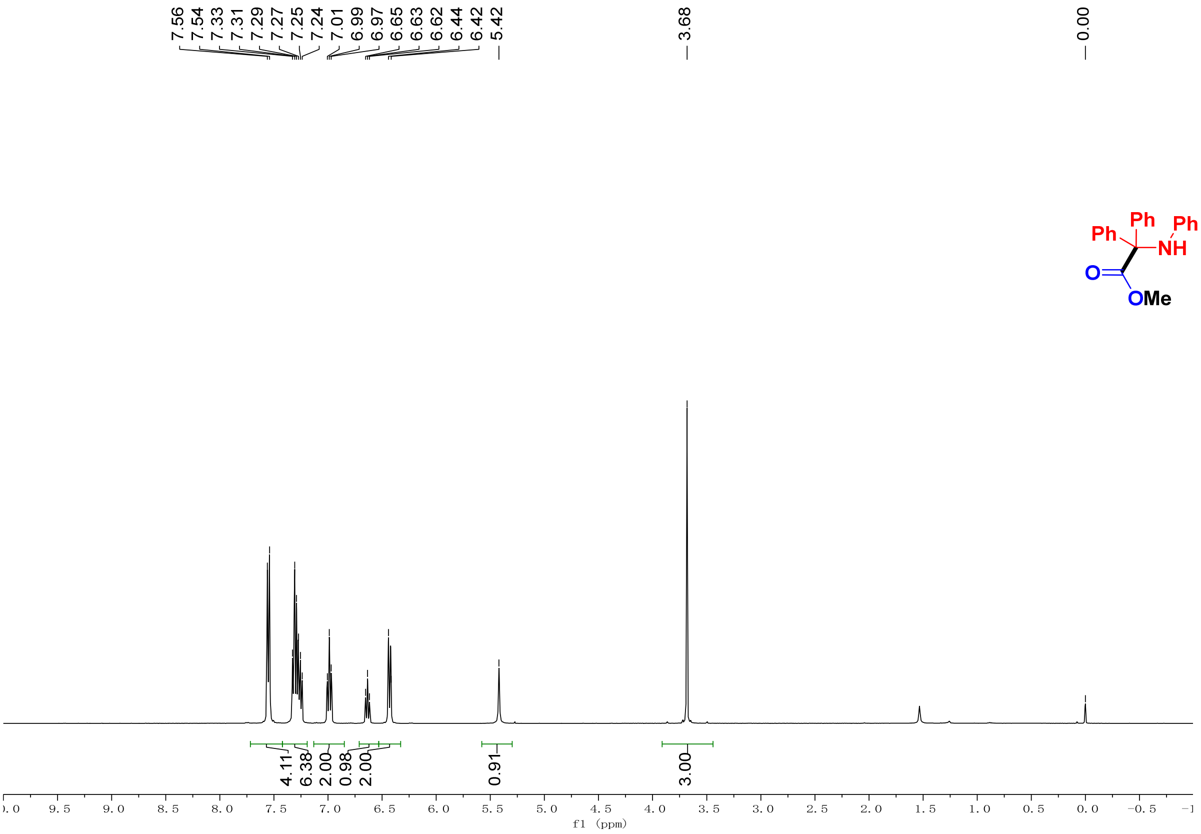

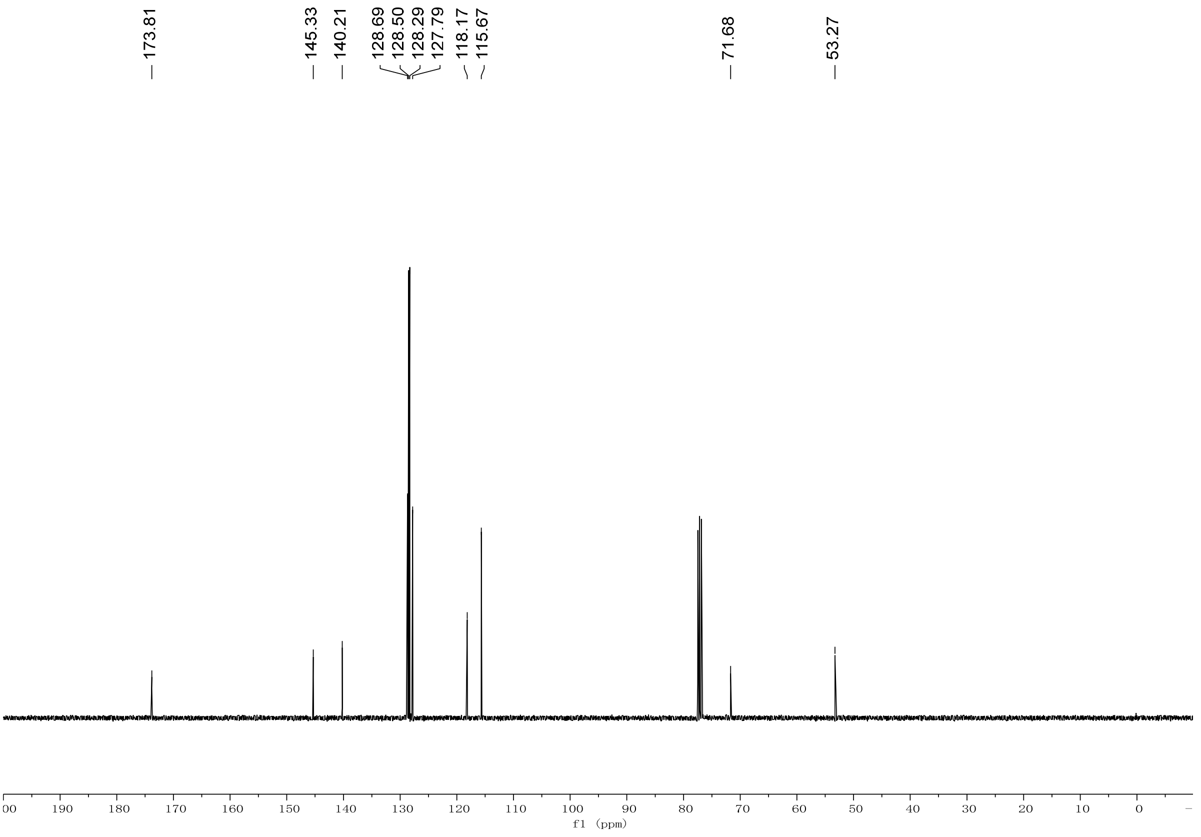

Supplementary Figure 16. ^{1}H, $^{13}C\{^{1}H\}$ and ^{19}F NMR spectra of compound **3l** in $CDCl_3$

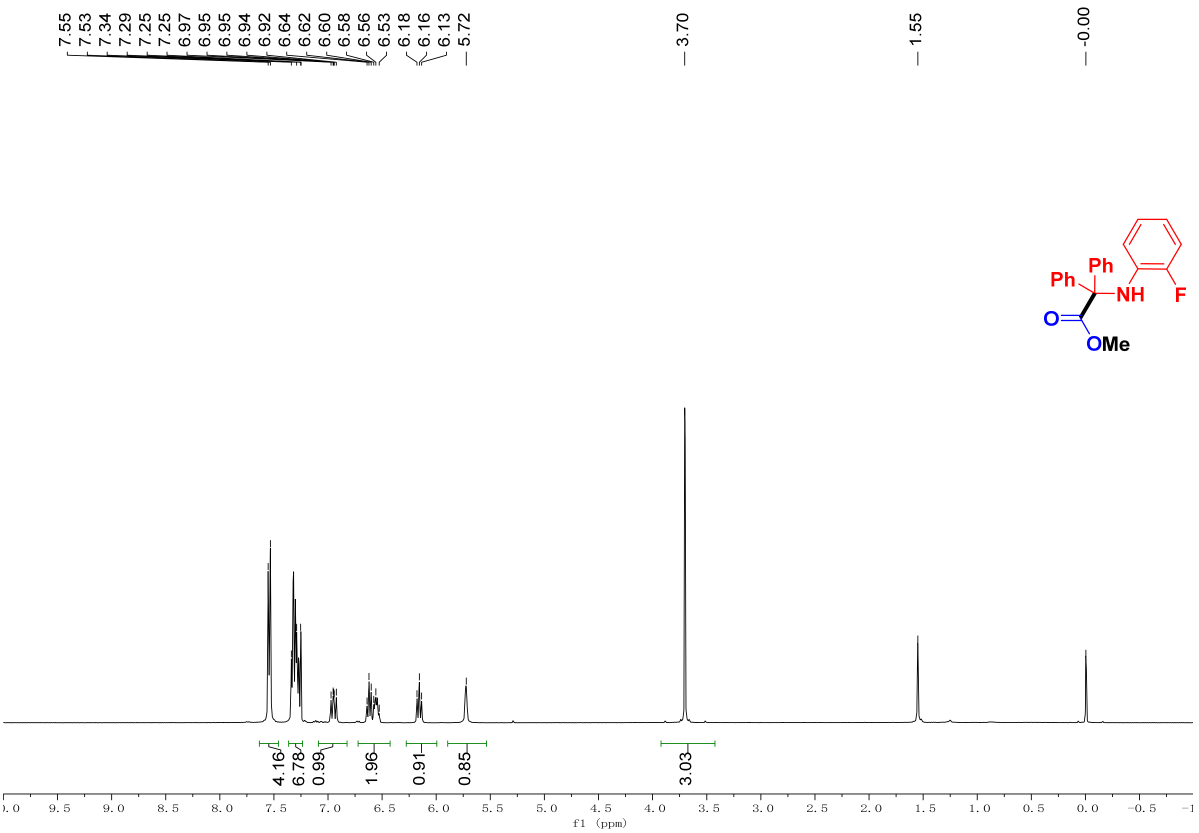

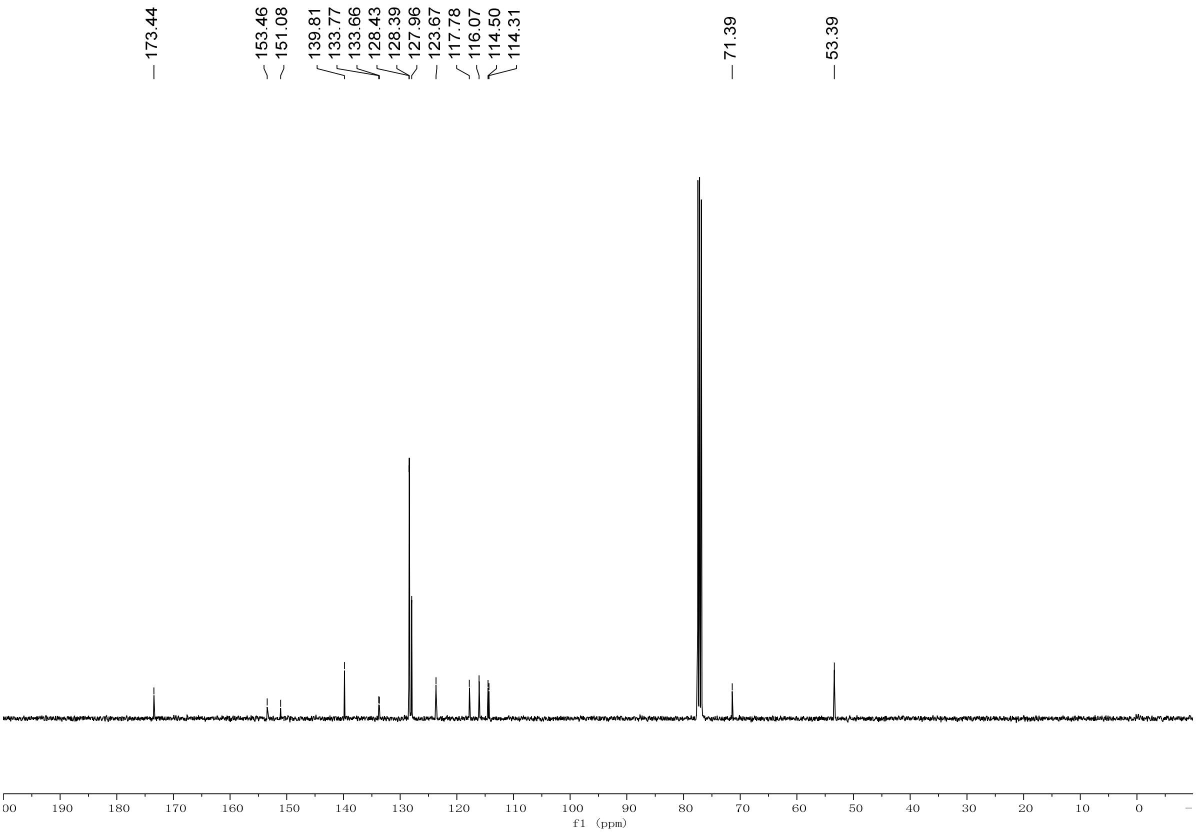

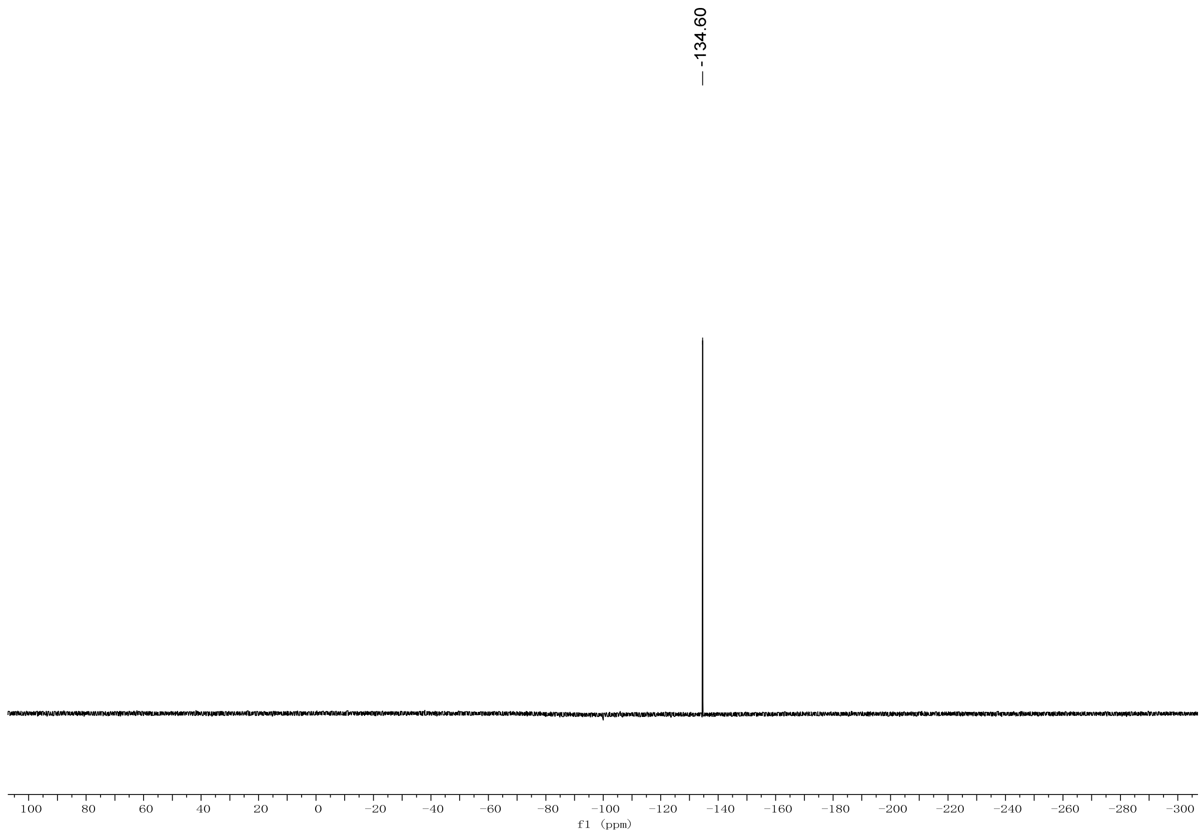
-134.60
100 80 60 40 20 0 -20 -40 -60 -80 -100 -120 -140 -160 -180 -200 -220 -240 -260 -280 -300
f1 (ppm)

Supplementary Figure 17. ^{1}H and $^{13}C\{^{1}H\}$ NMR spectra of compound **3m** in $CDCl_3$

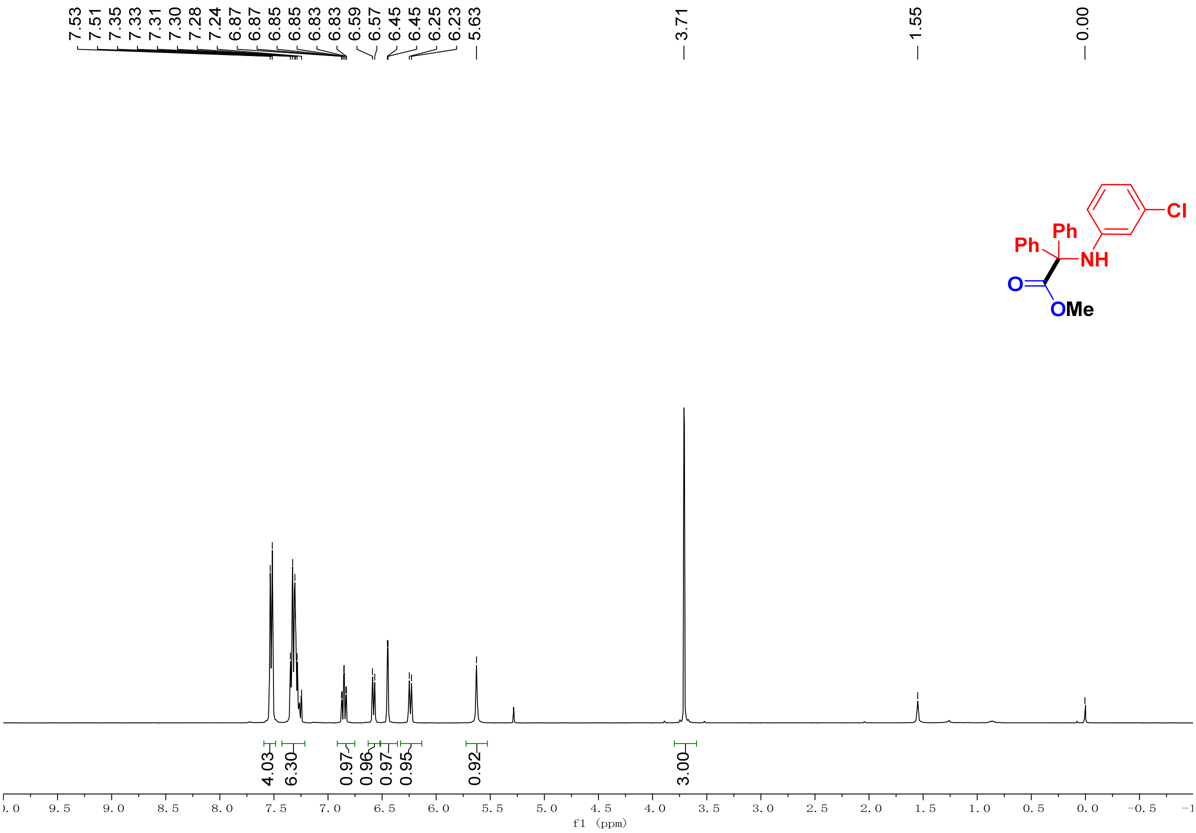

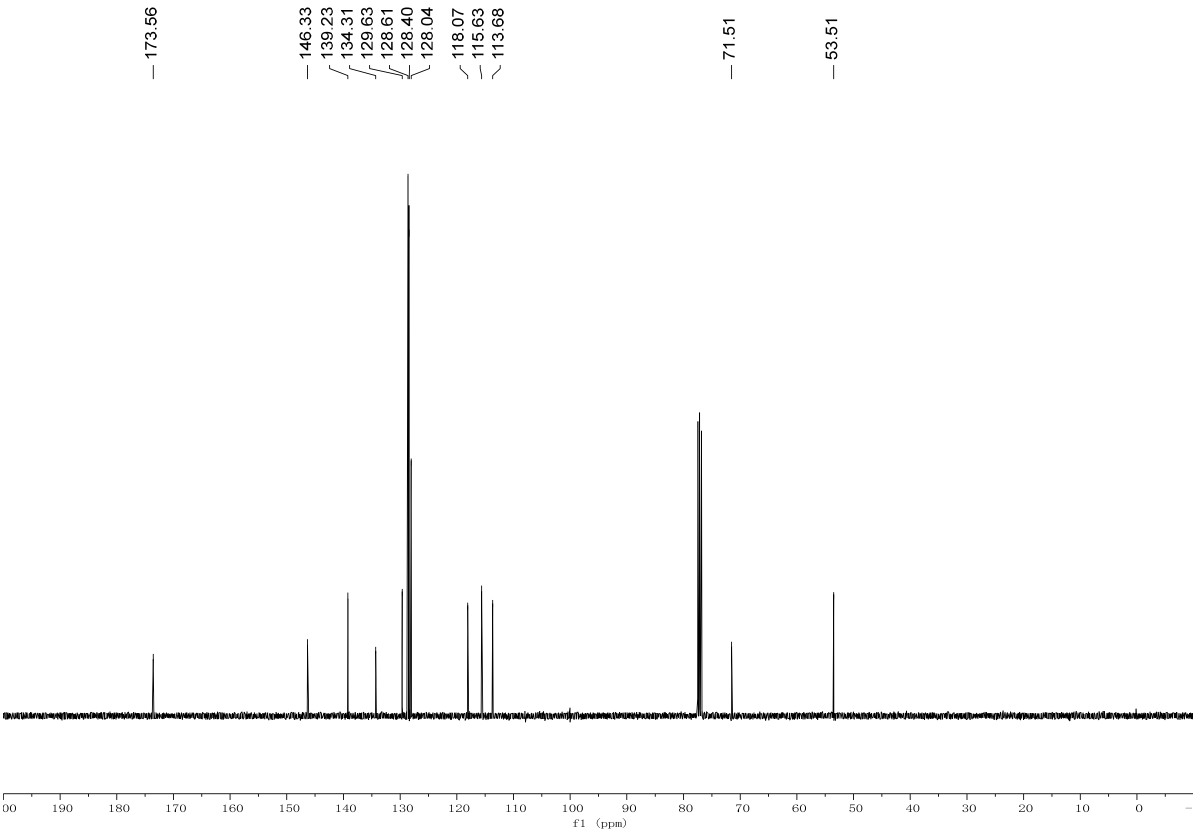

Supplementary Figure 18. ^{1}H and ^{13}C{^{1}H} NMR spectra of compound **3n** in $CDCl_3$

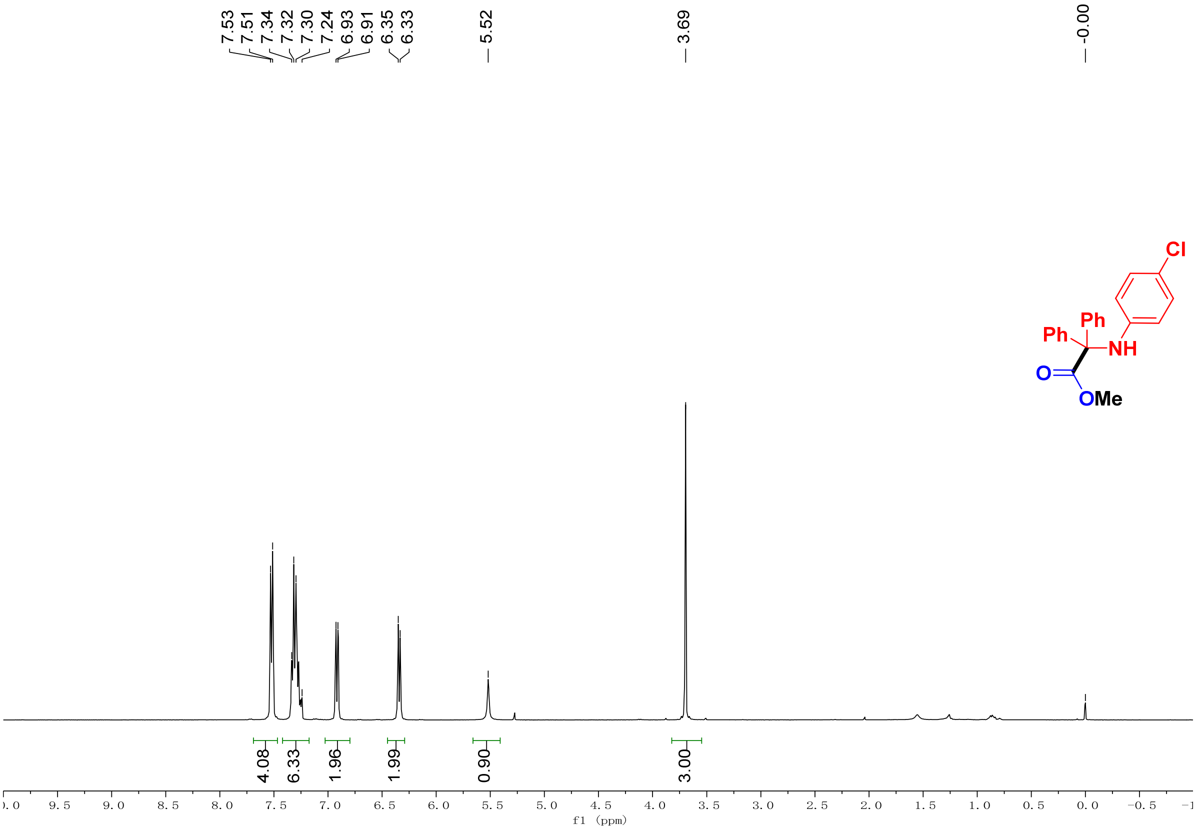

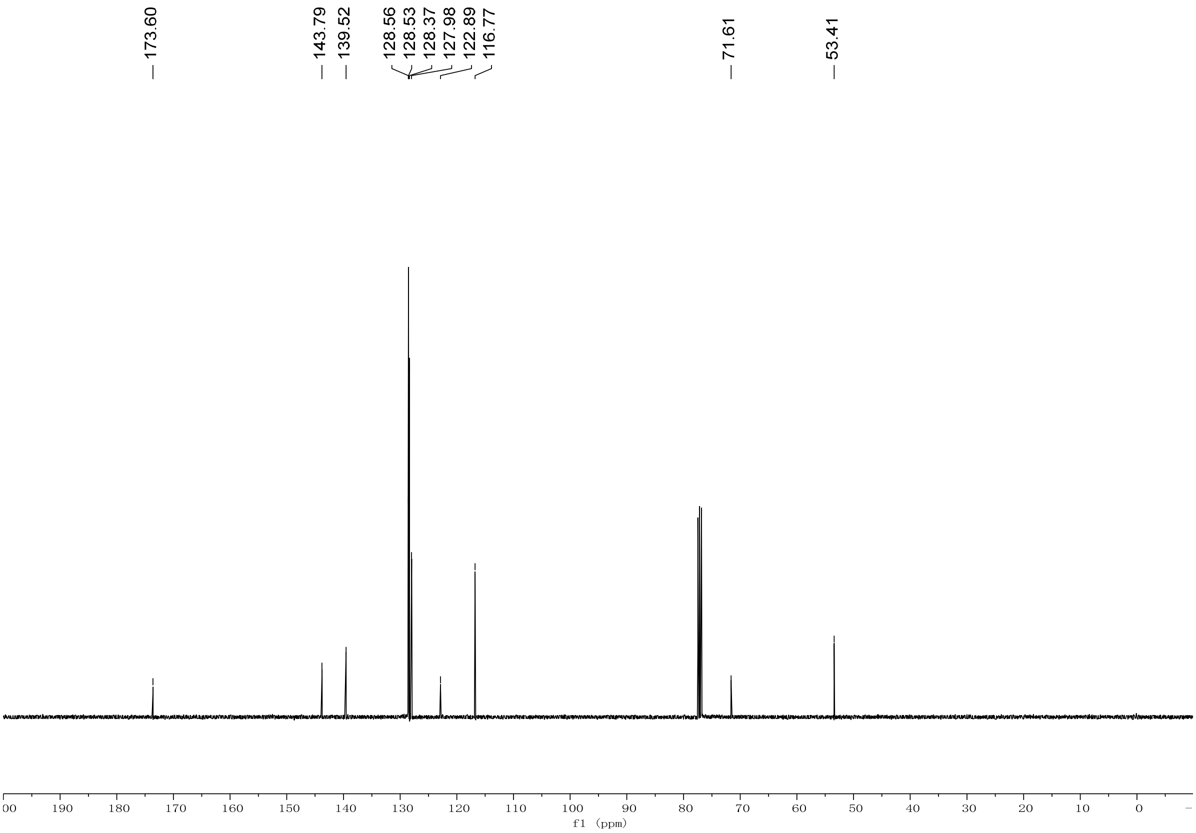

Supplementary Figure 19. ^{1}H, ^{13}C{^{1}H} and ^{19}F NMR spectra of compound **3o** in $CDCl_3$

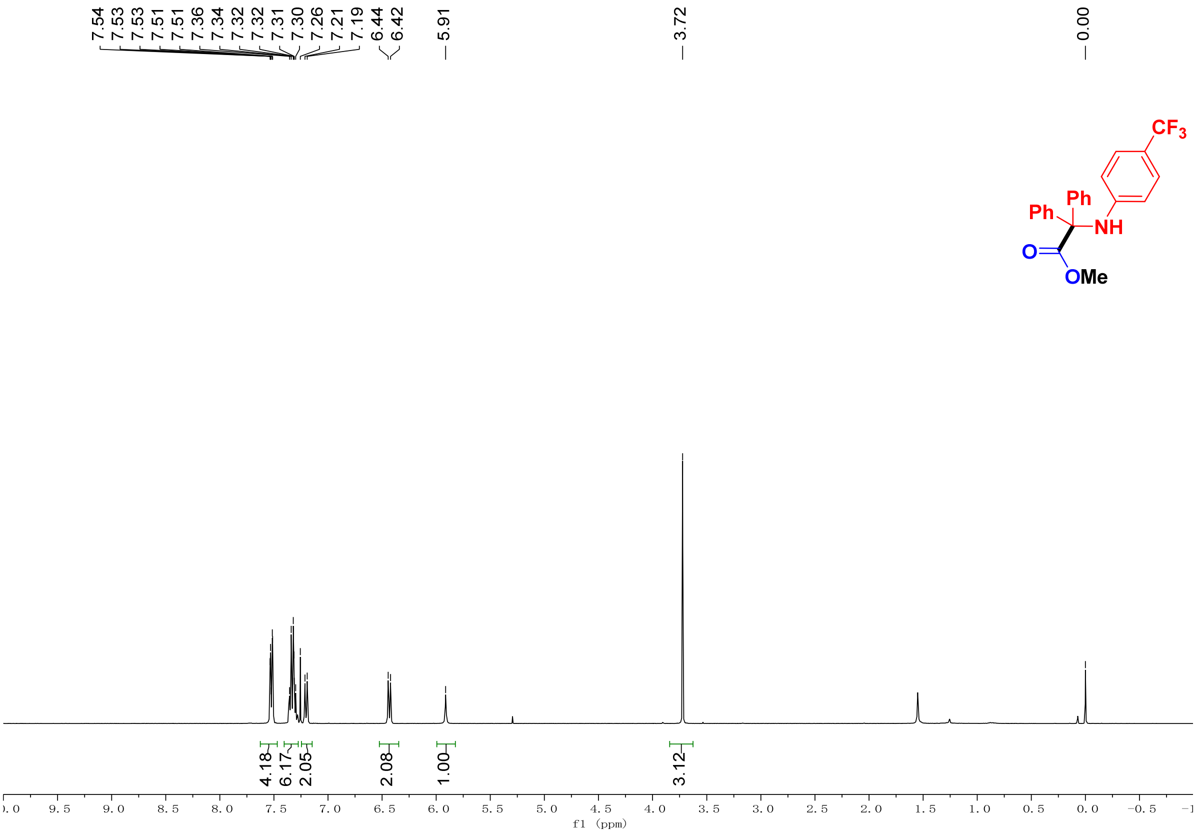

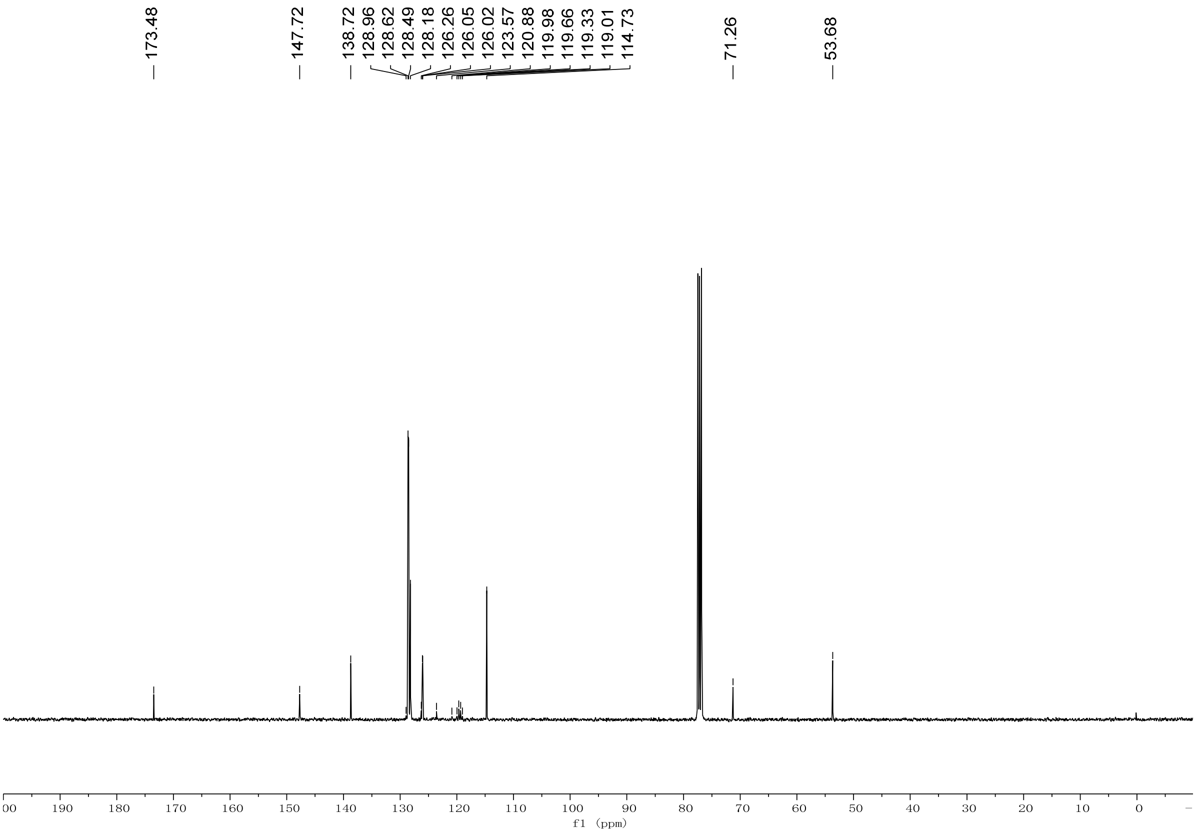

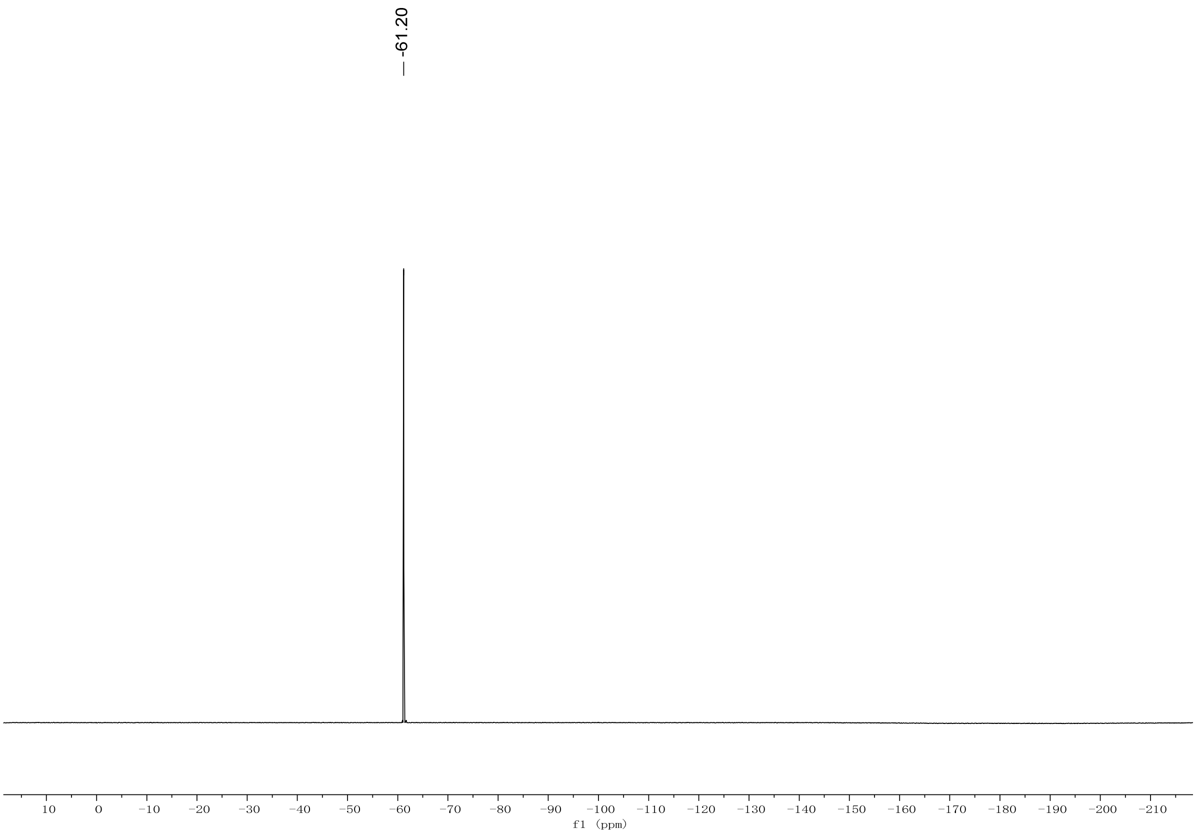
-61.20
10 0 −10 −20 −30 −40 −50 −60 −70 −80 −90 −100 −110 −120 −130 −140 −150 −160 −170 −180 −190 −200 −210
f1 (ppm)

Supplementary Figure 20. ^{1}H, $^{13}C\{^{1}H\}$ NMR spectra of compound **3p** in $CDCl_3$

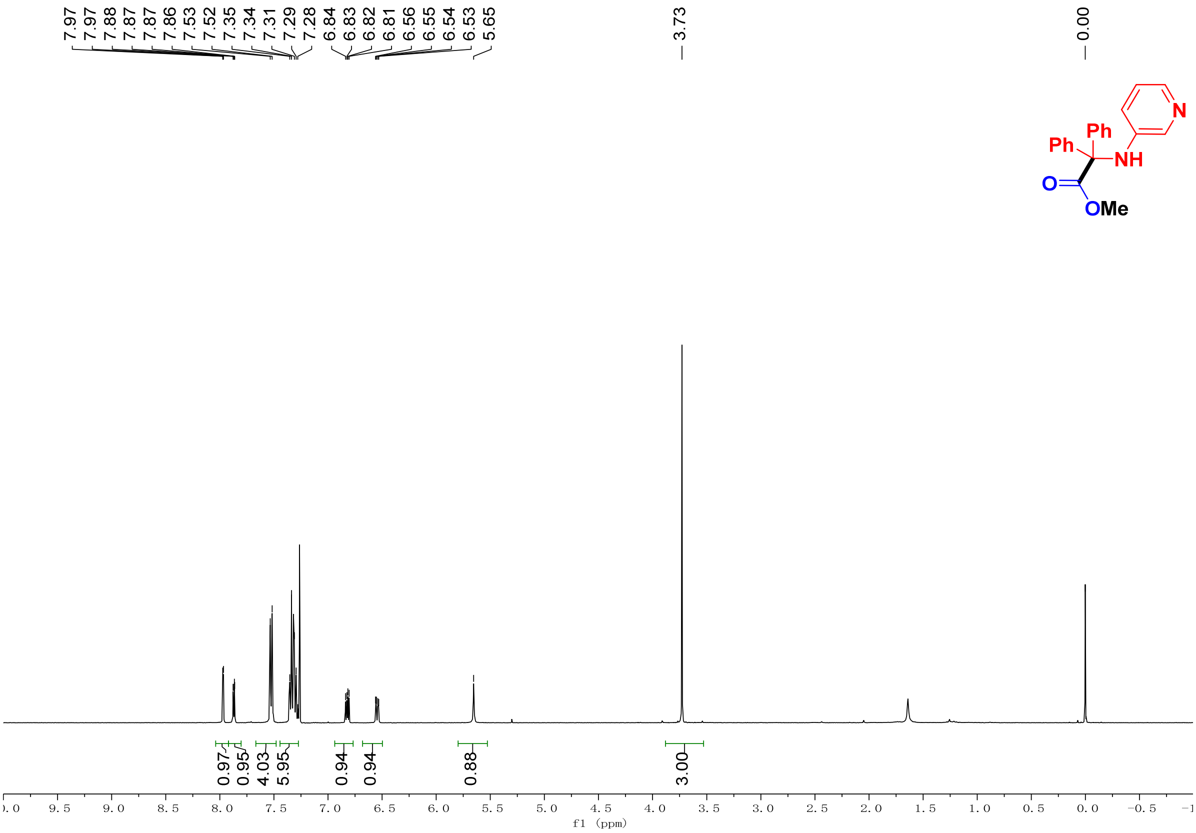

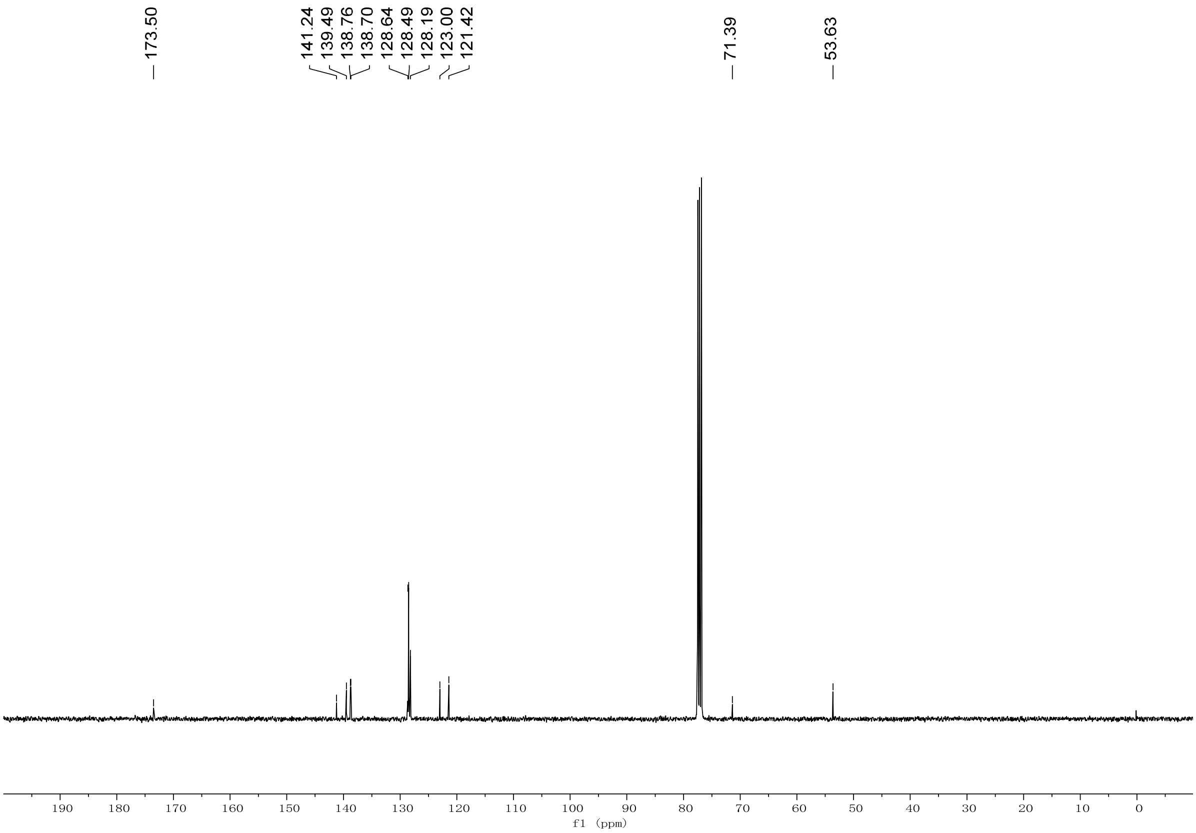

Supplementary Figure 21. ^{1}H, $^{13}C\{^{1}H\}$ and ^{19}F NMR spectra of compound **3q** in $CDCl_3$

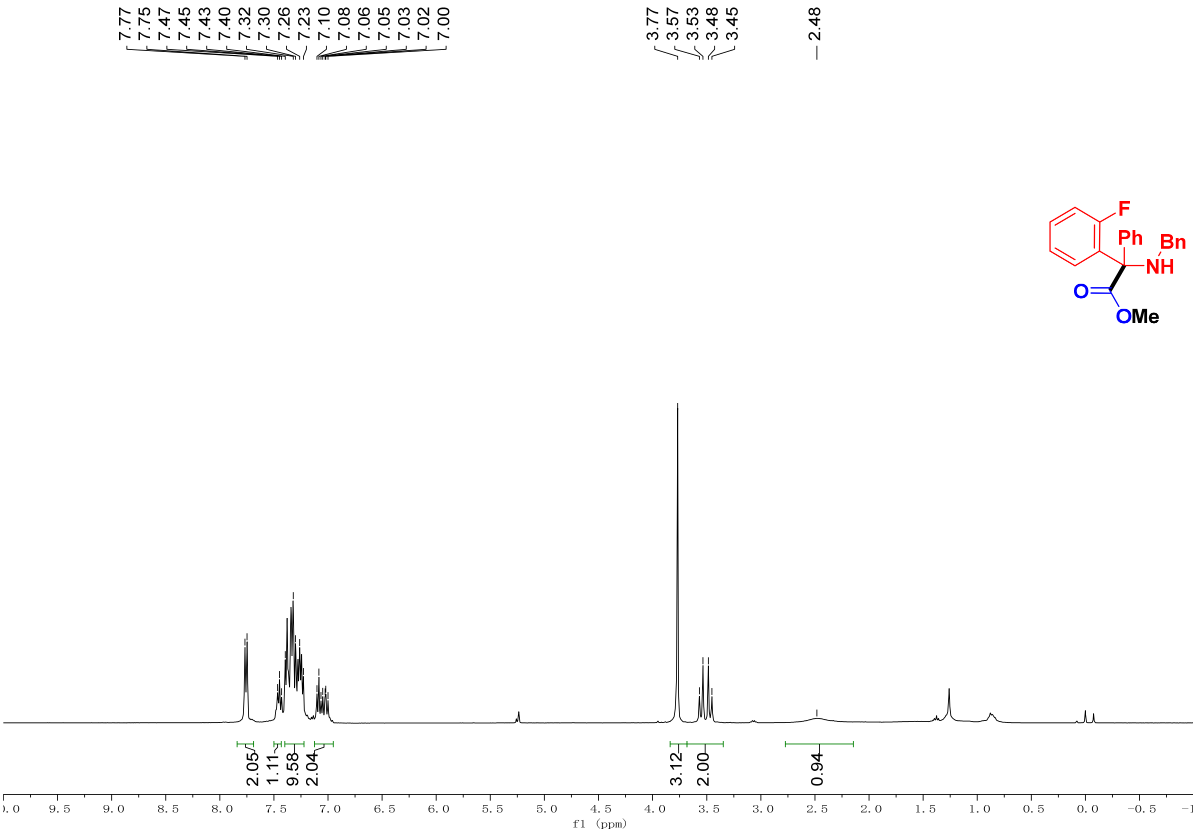

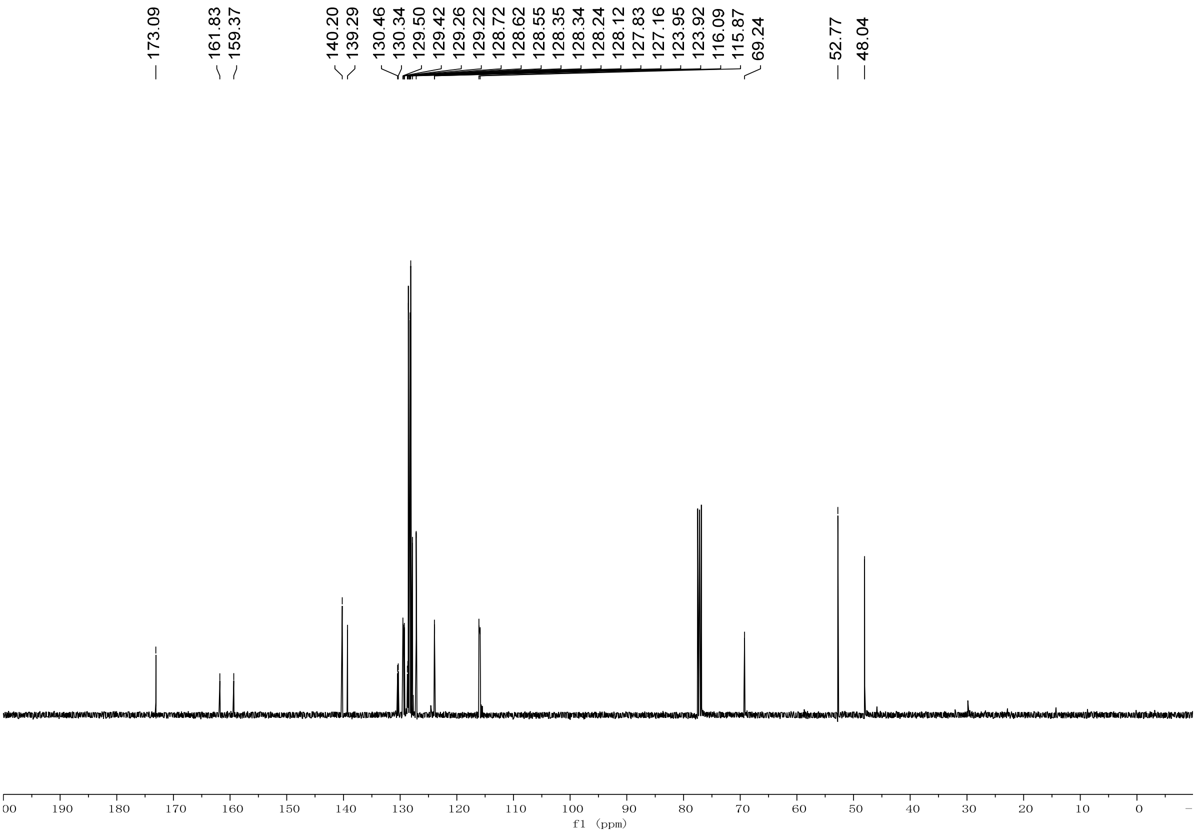

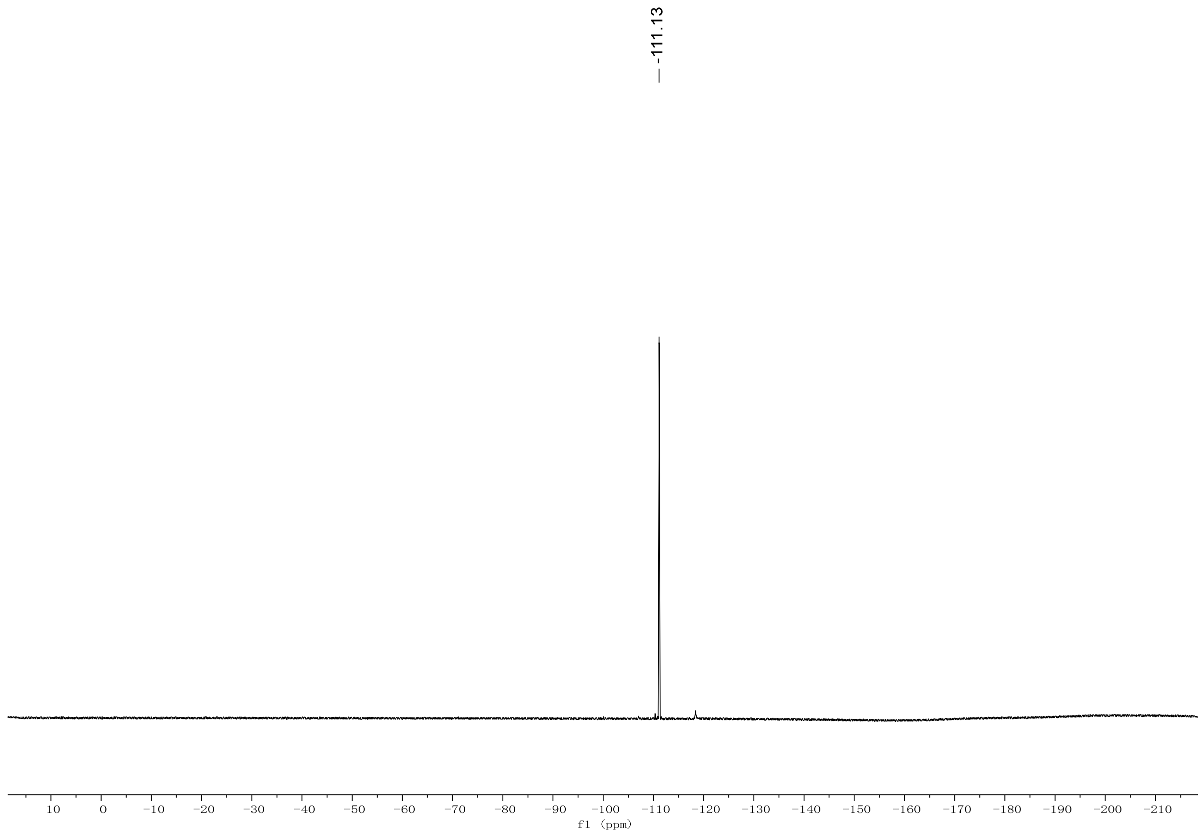
-111.13
10
0
-10
-20
-30
-40
-50
-60
-70
-80
-90
-100
-110
-120
-130
-140
-150
-160
-170
-180
-190
-200
-210
f1 (ppm)

Supplementary Figure 22. ^{1}H and ^{13}C{^{1}H} NMR spectra of compound **3r** in $CDCl_3$

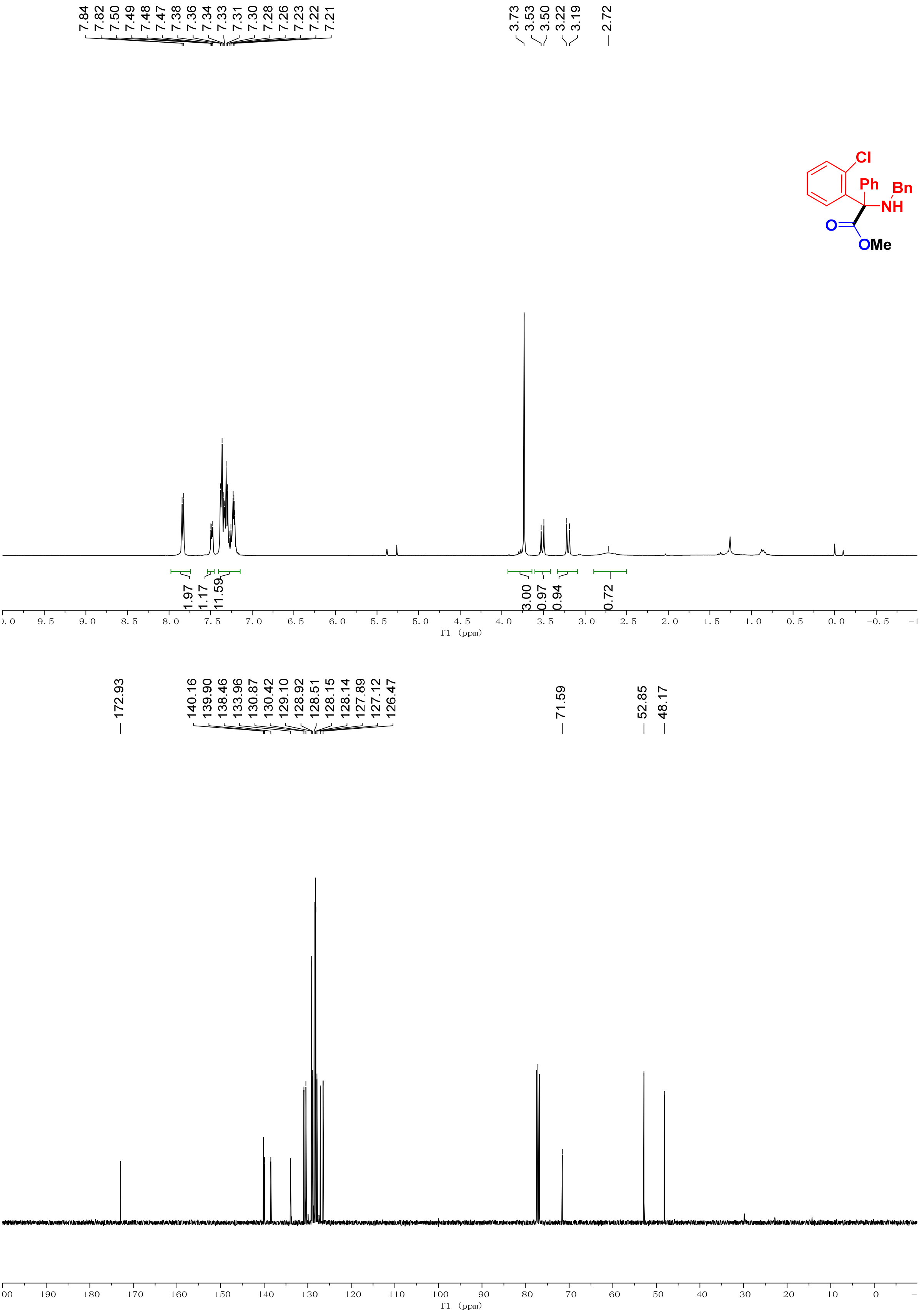

Supplementary Figure 23. ^{1}H and ^{13}C{^{1}H} NMR spectra of compound **3s** in $CDCl_3$

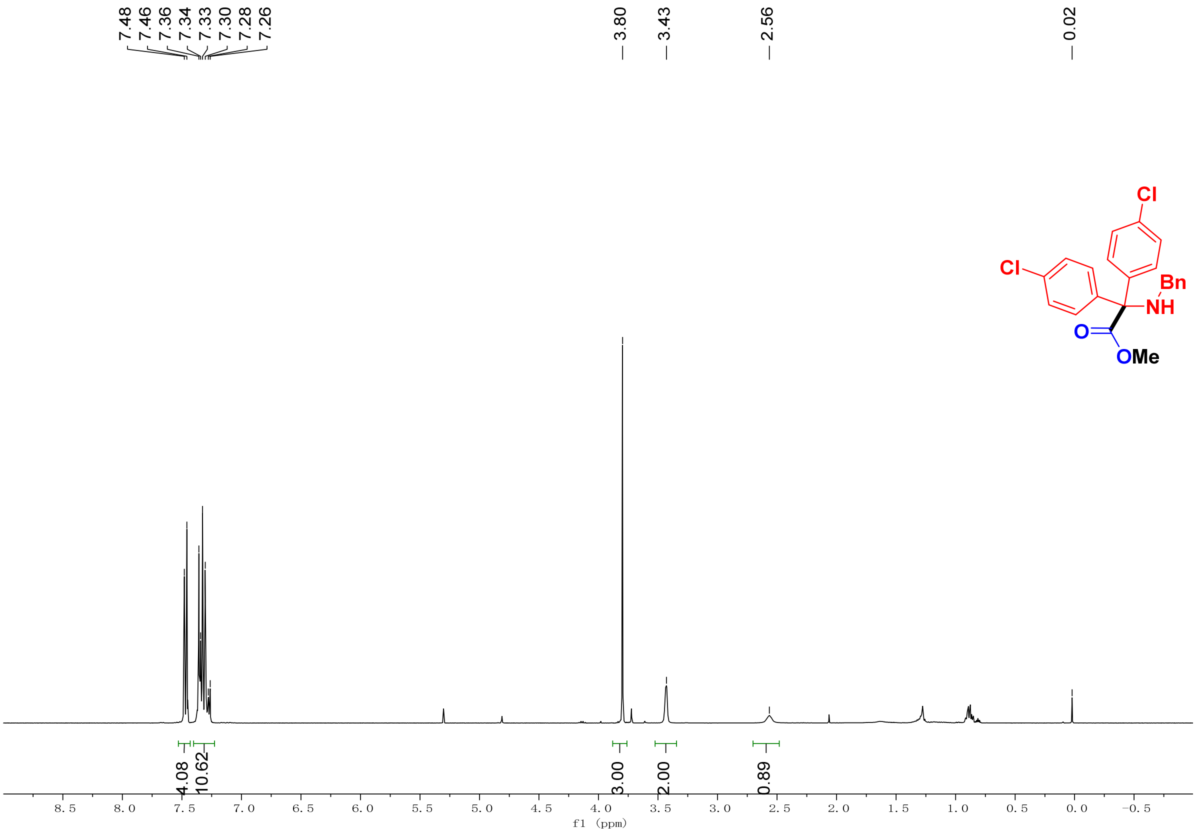

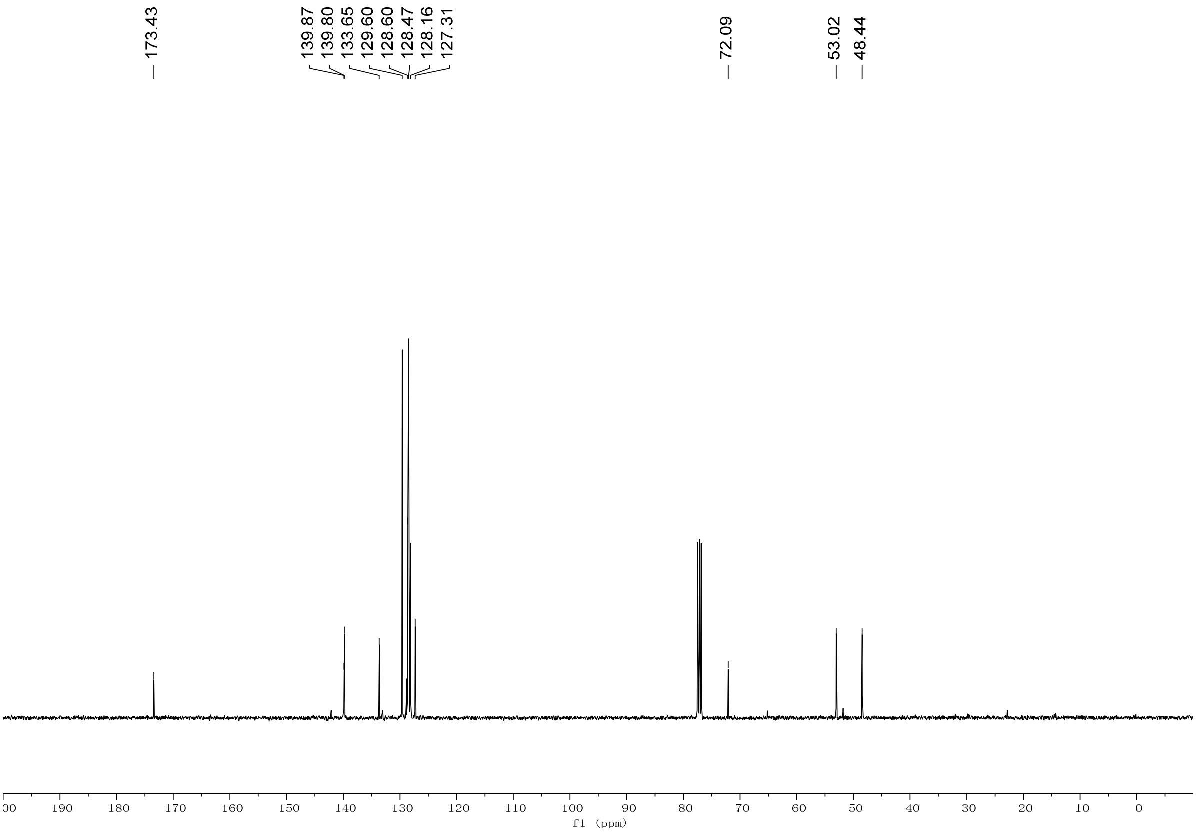

Supplementary Figure 24. ^{1}H and $^{13}C\{^{1}H\}$ NMR spectra of compound **3t** in $CDCl_3$

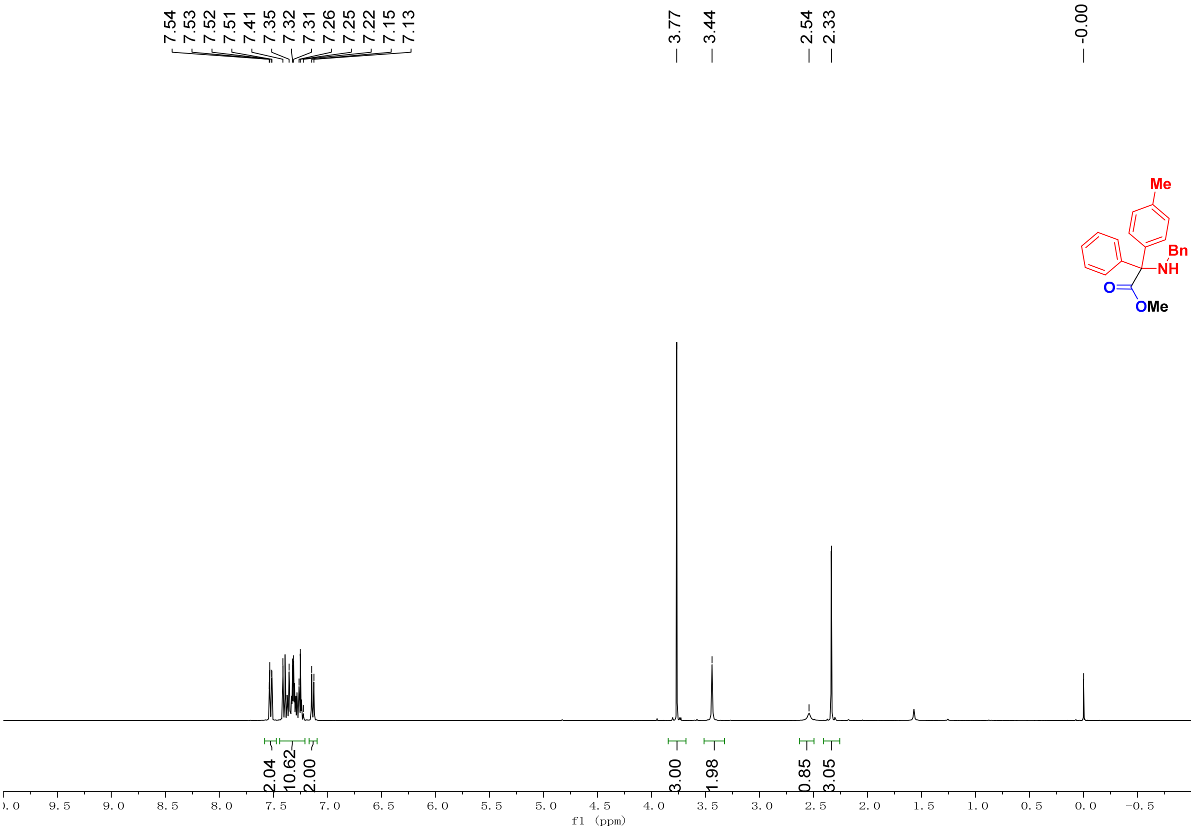

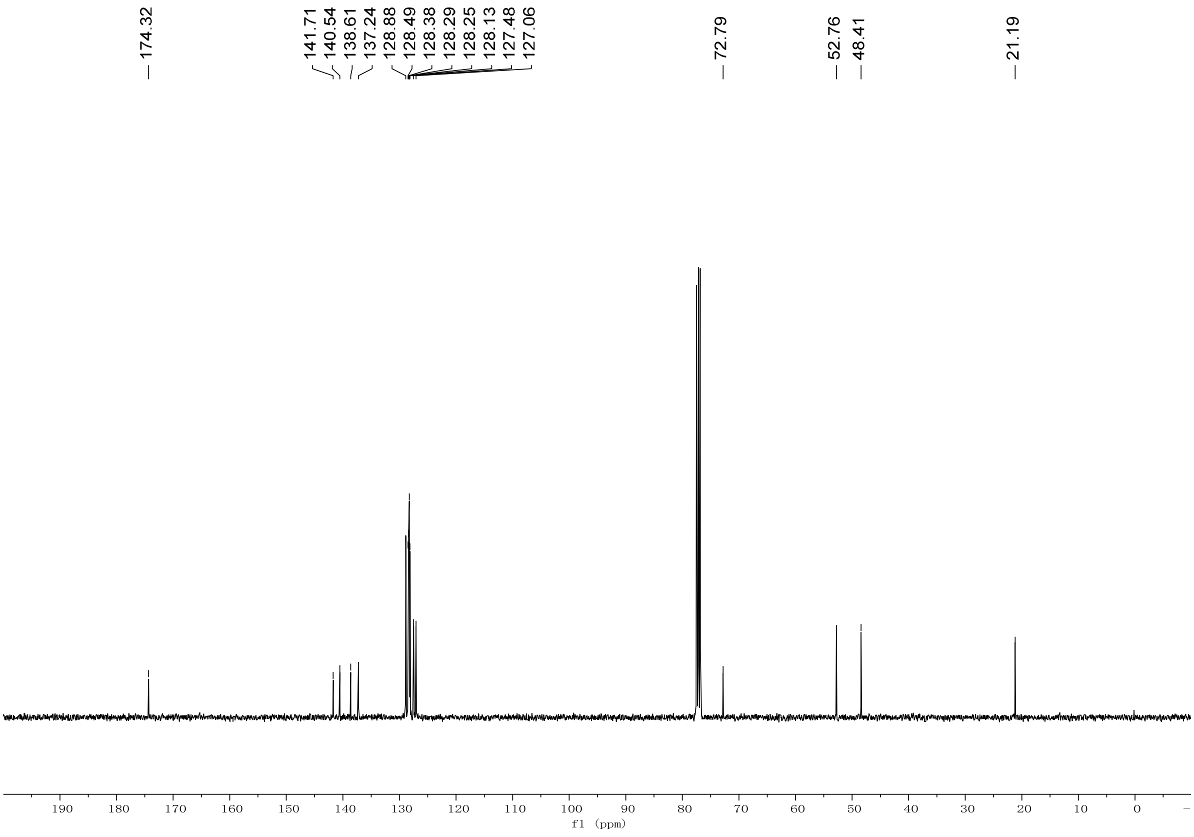

Supplementary Figure 25. ^{1}H and ^{13}C{^{1}H} NMR spectra of compound **3u** in $CDCl_3$

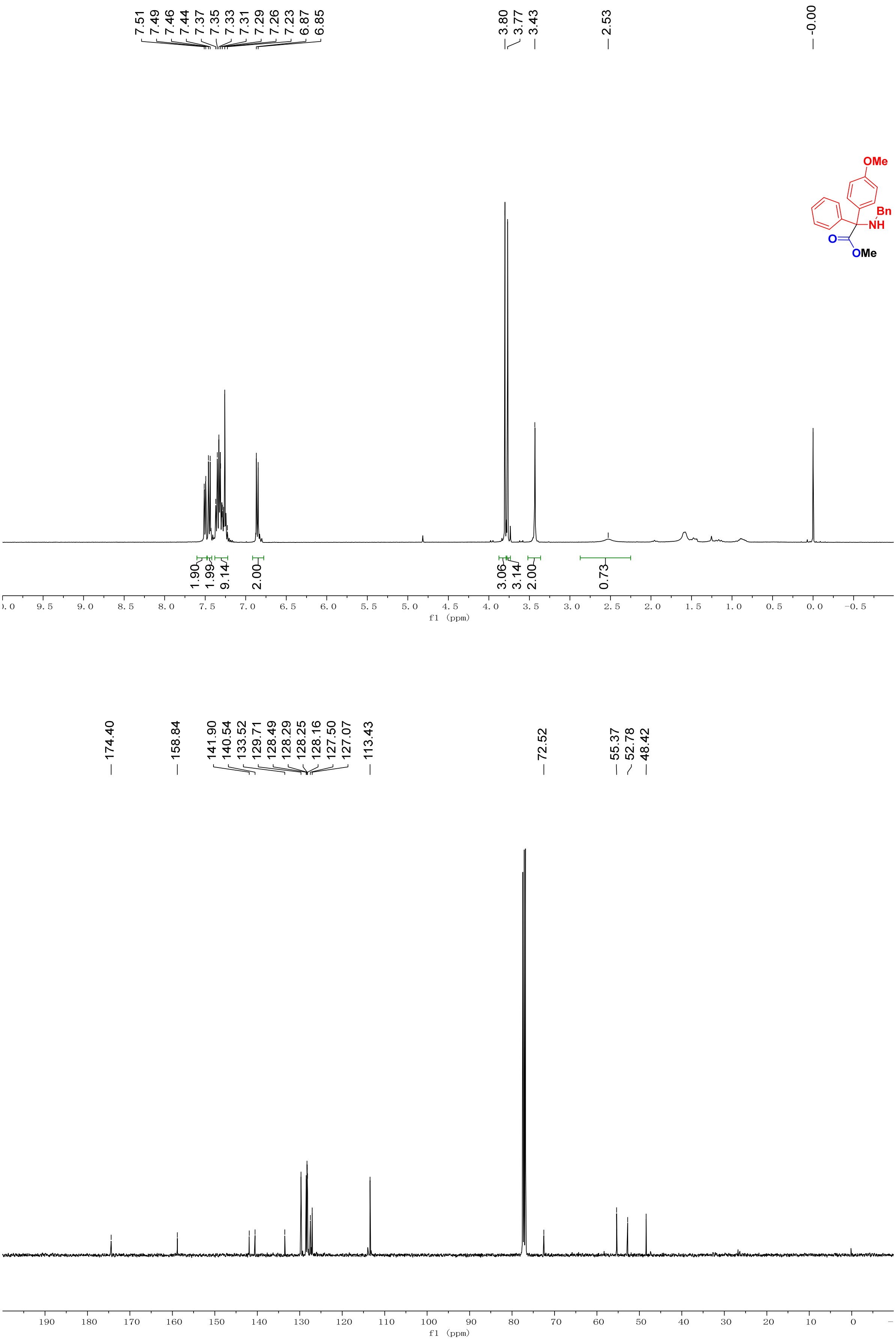

Supplementary Figure 26. ^{1}H and $^{13}C\{^{1}H\}$ NMR spectra of compound **3v** in $CDCl_3$

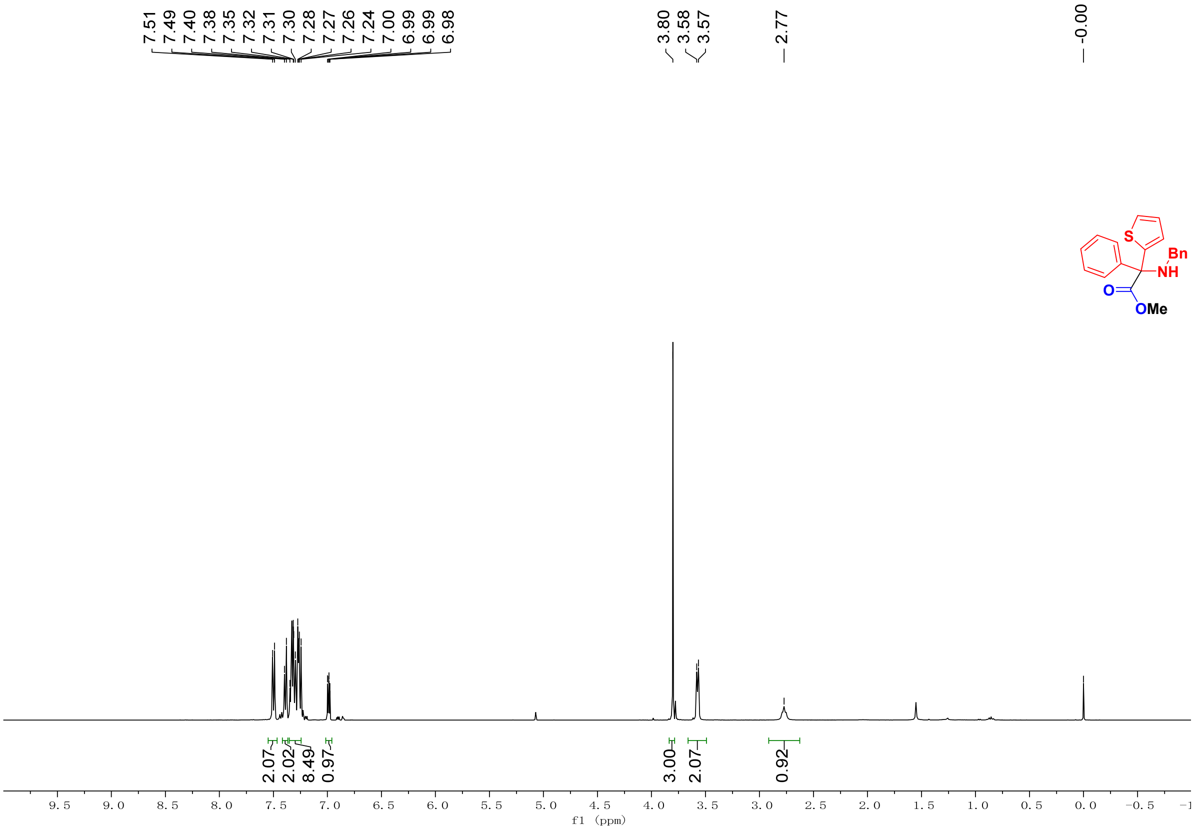

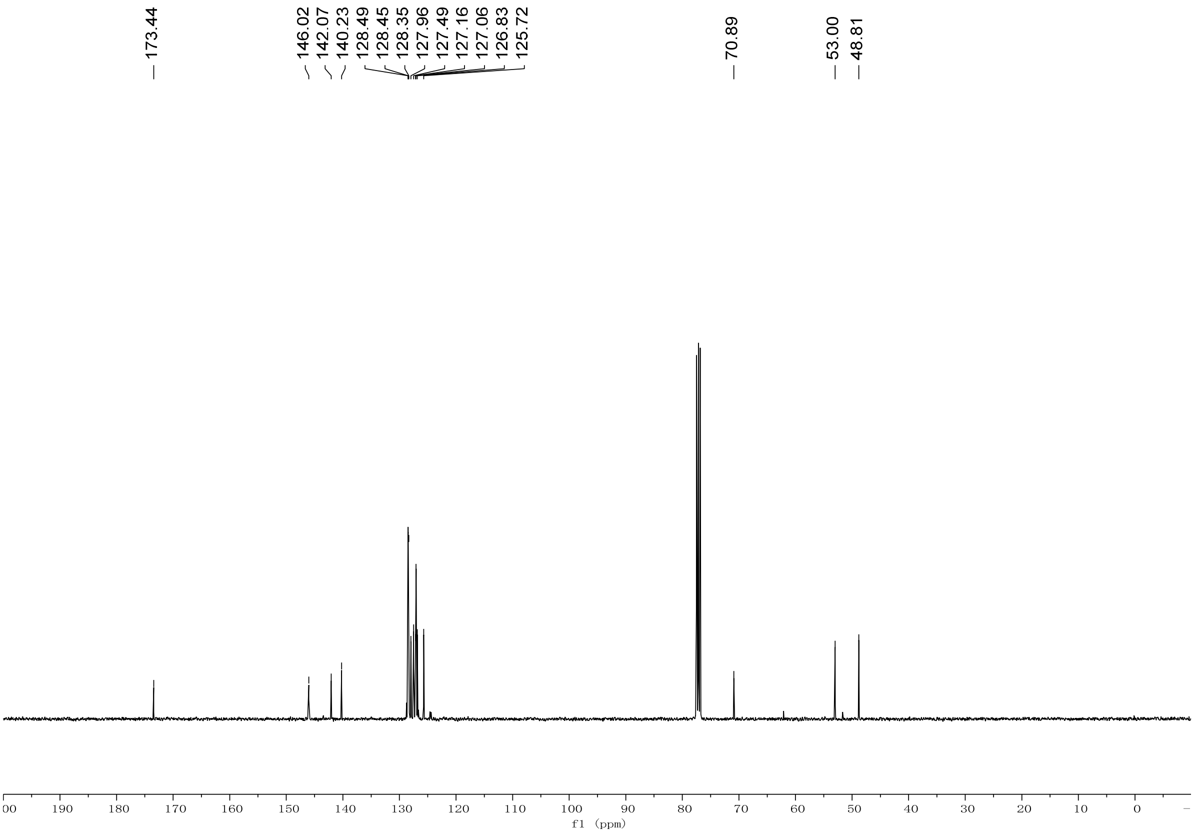

Supplementary Figure 27. ^{1}H and ^{13}C{^{1}H} NMR spectra of compound **3w** in $CDCl_3$

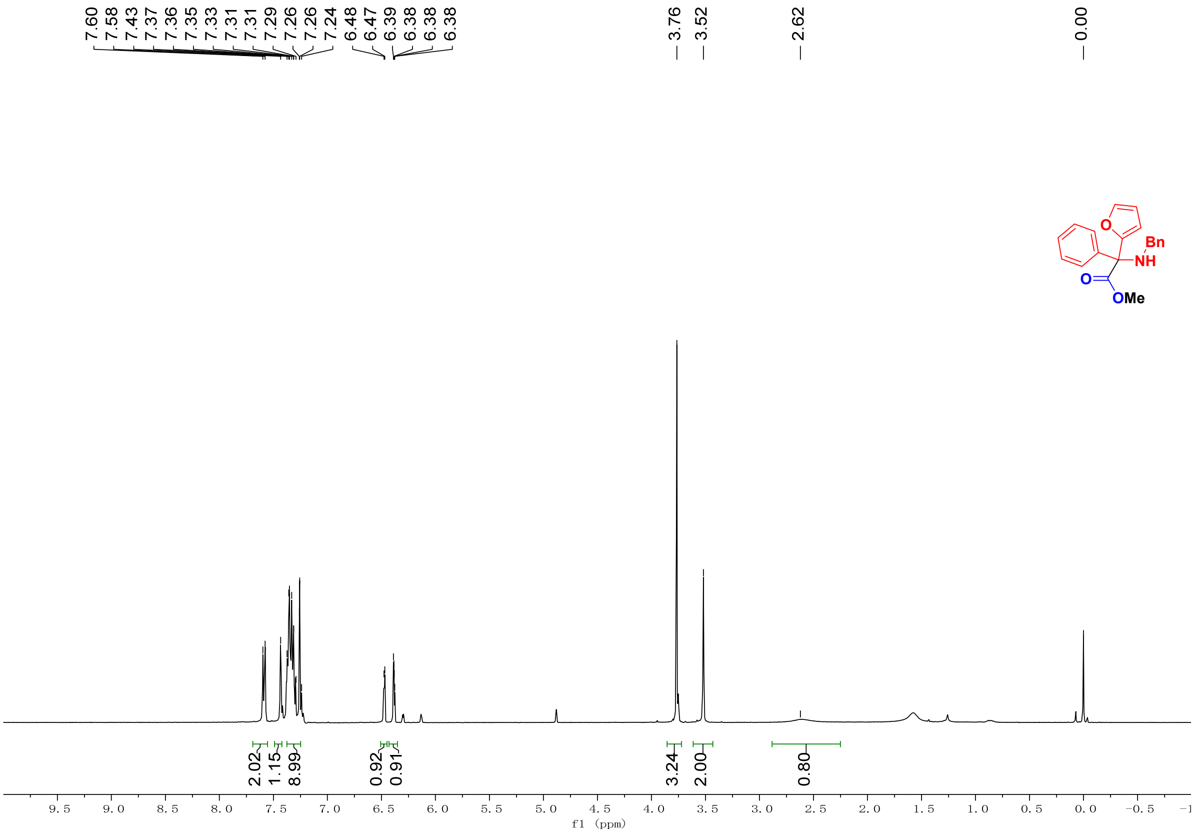

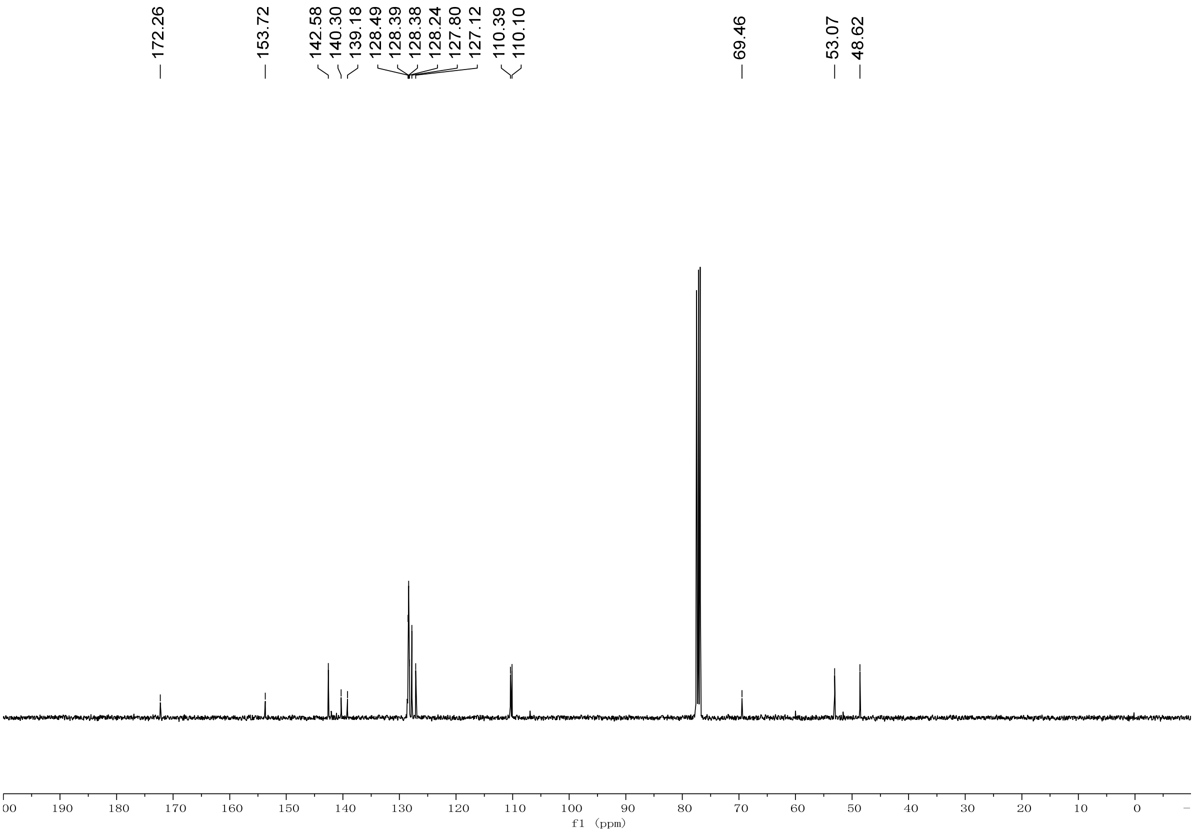

Supplementary Figure 28. ^{1}H and ^{13}C{^{1}H} NMR spectra of compound **3x** in $CDCl_3$

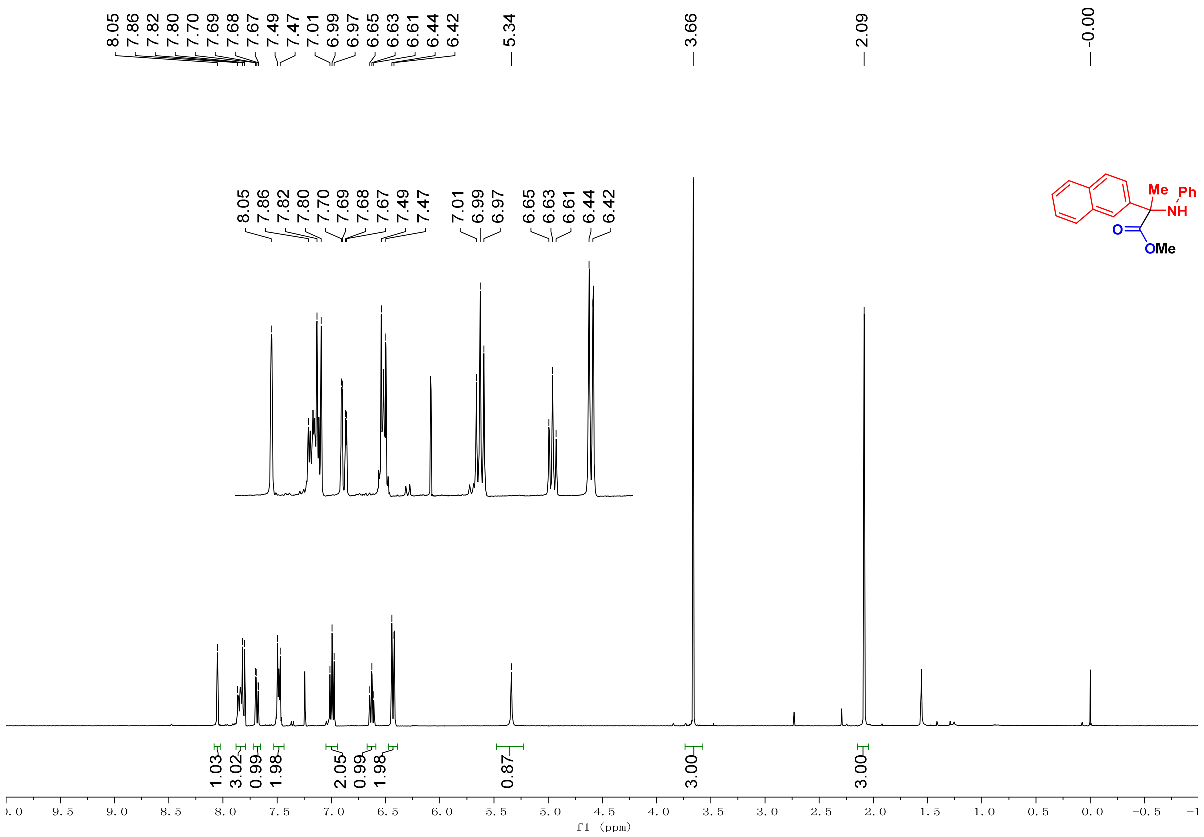

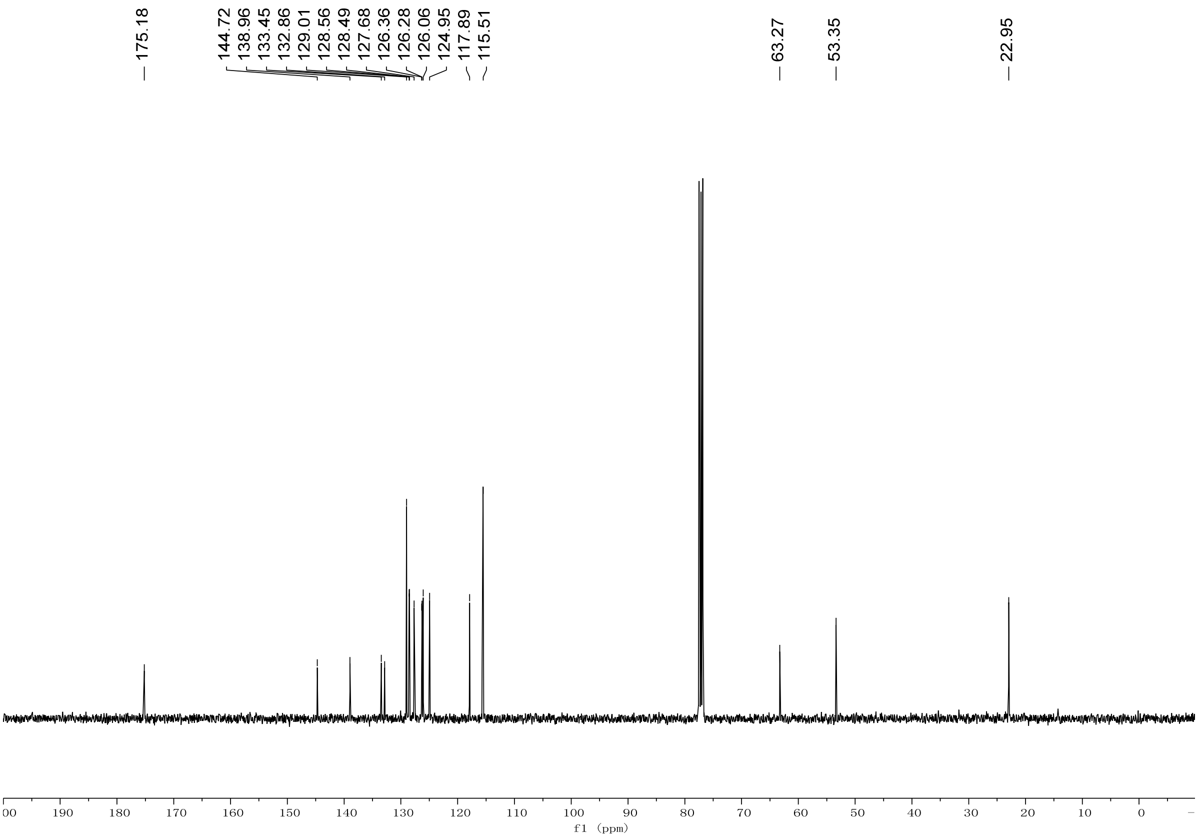

Supplementary Figure 29. ^{1}H and ^{13}C{^{1}H} NMR spectra of desired α-amino acid dicyclohexylamine salt **4aa** in $CDCl_3$

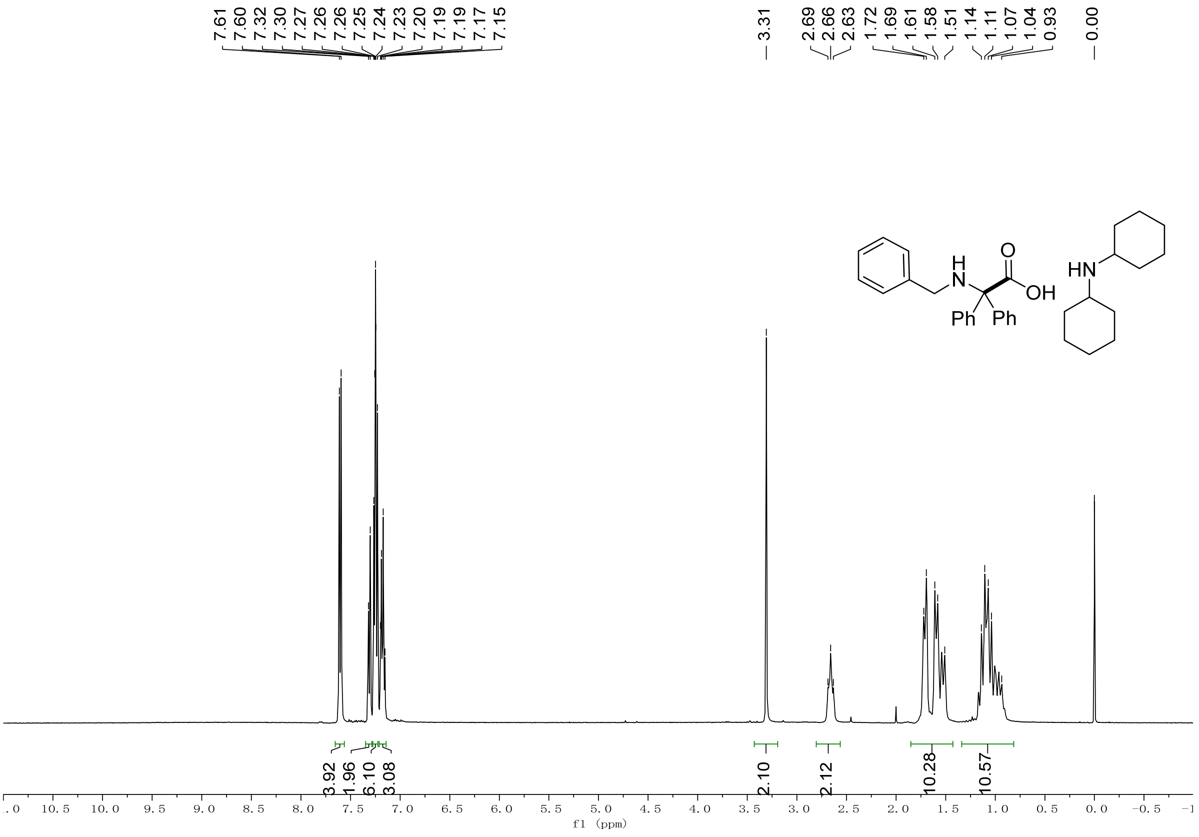

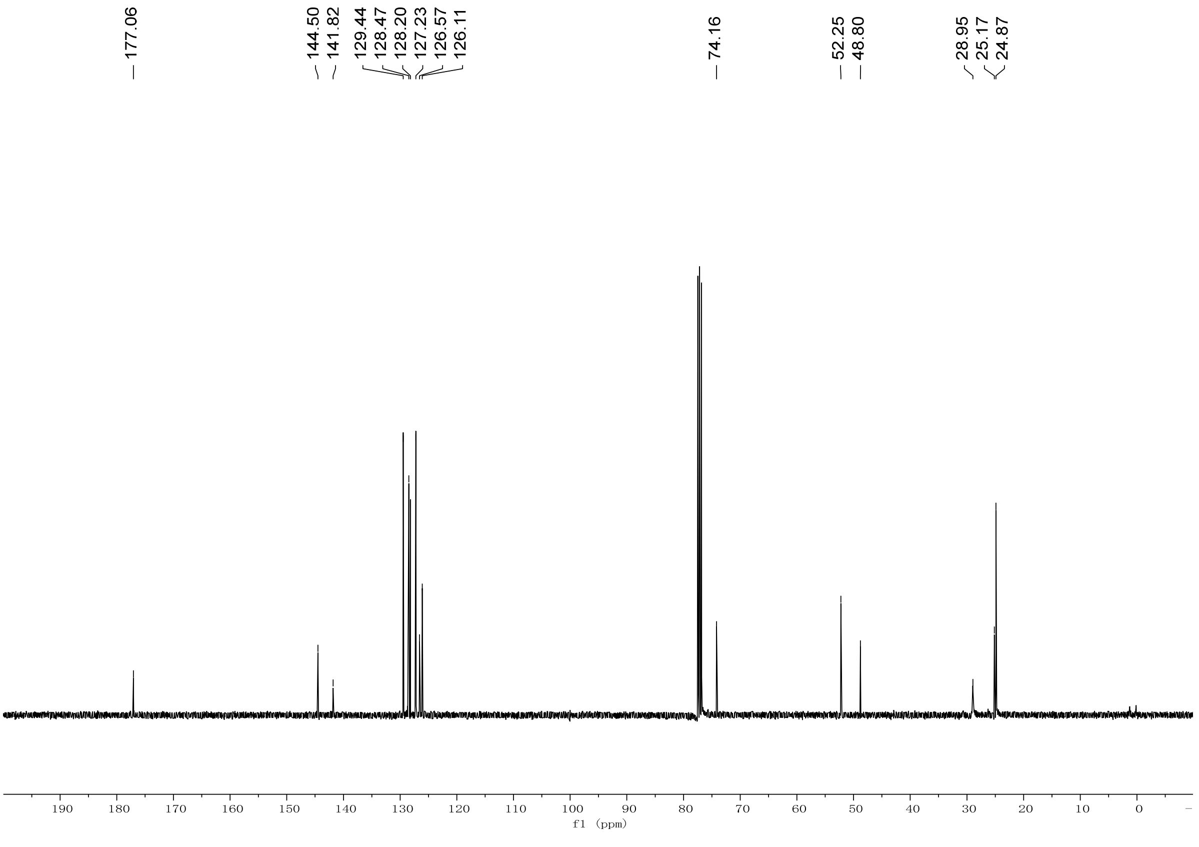

Supplementary Figure 30. ^{1}H and ^{13}C{^{1}H} NMR spectra of desired α-amino acid dicyclohexylamine salt **4ab** in $CDCl_3$

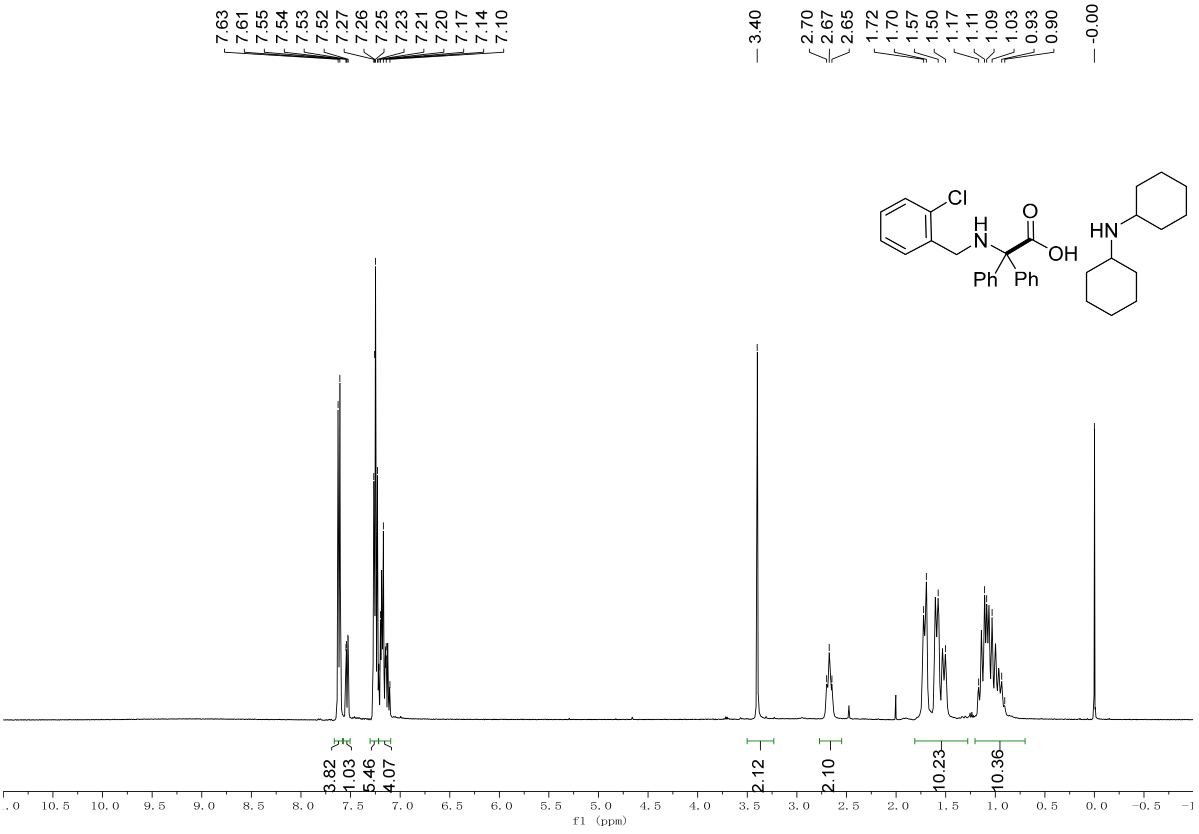

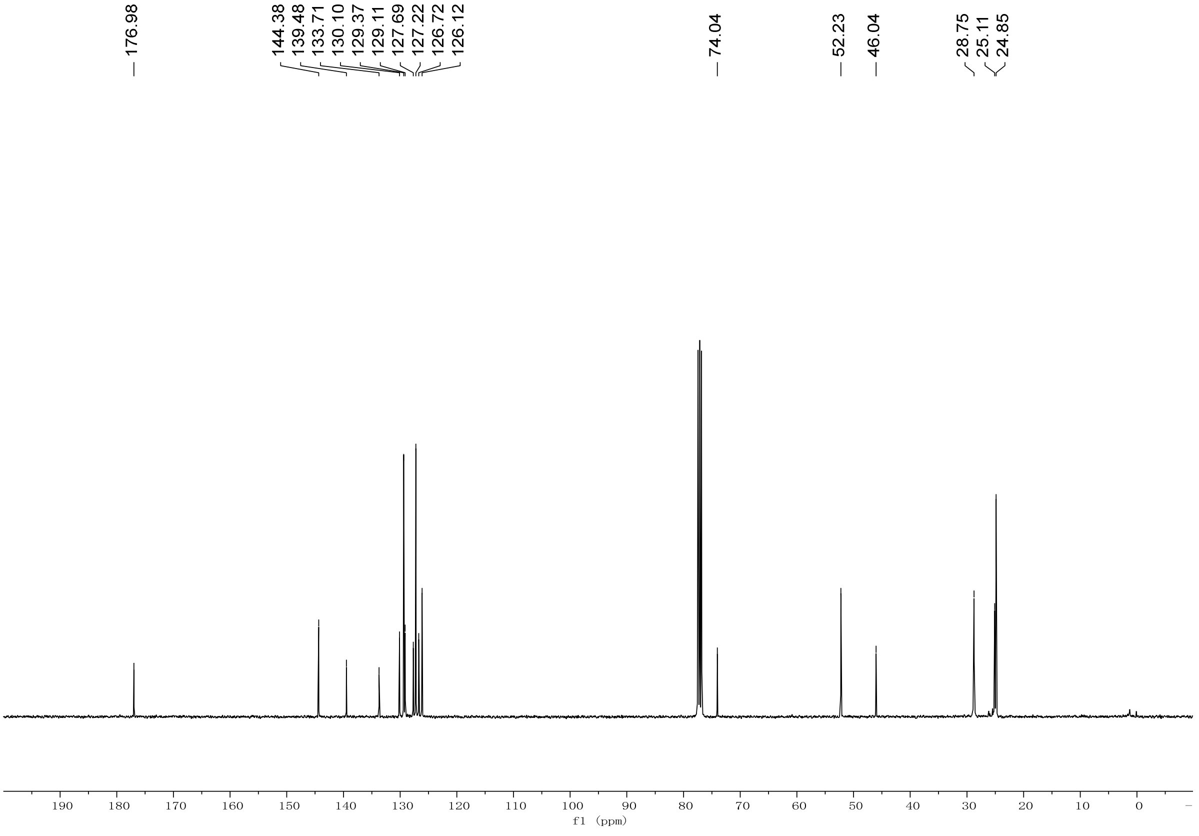

Supplementary Figure 31. ^{1}H and $^{13}C\{^{1}H\}$ NMR spectra of desired α-amino acid dicyclohexylamine salt **4ac** in $CDCl_3$

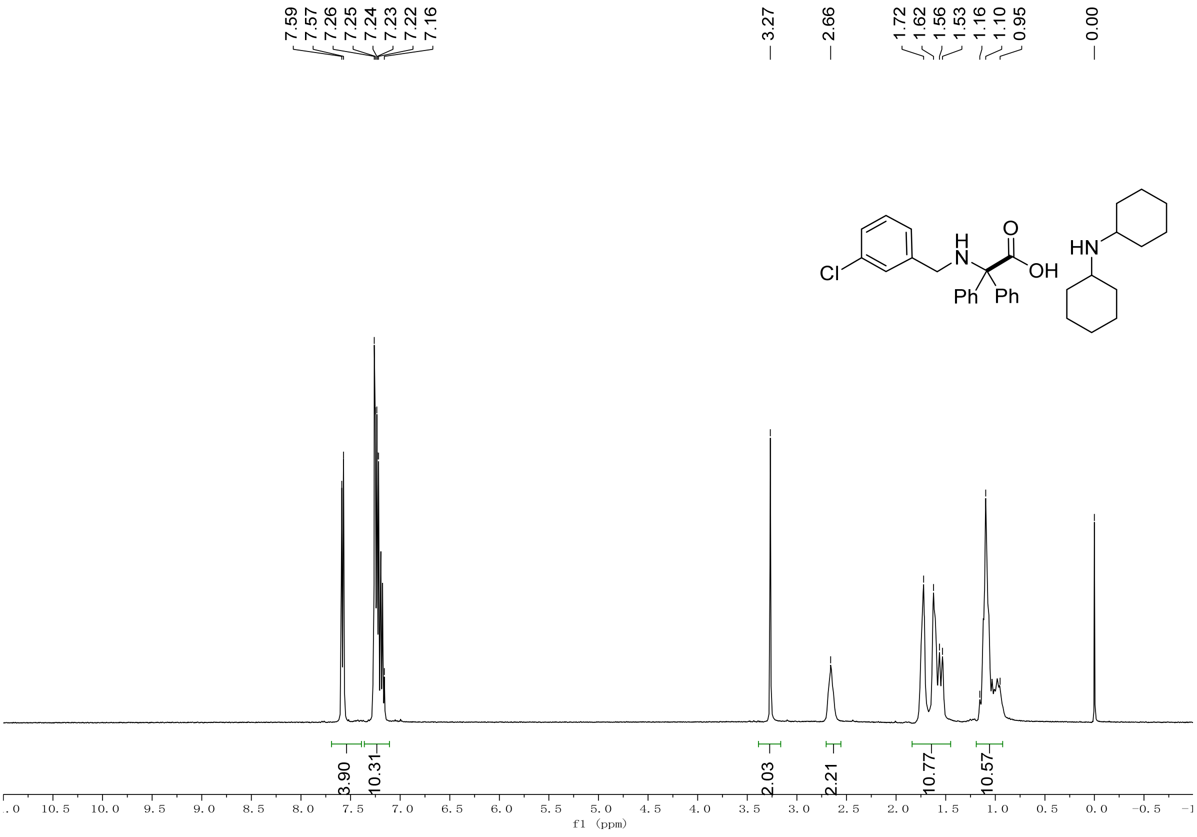

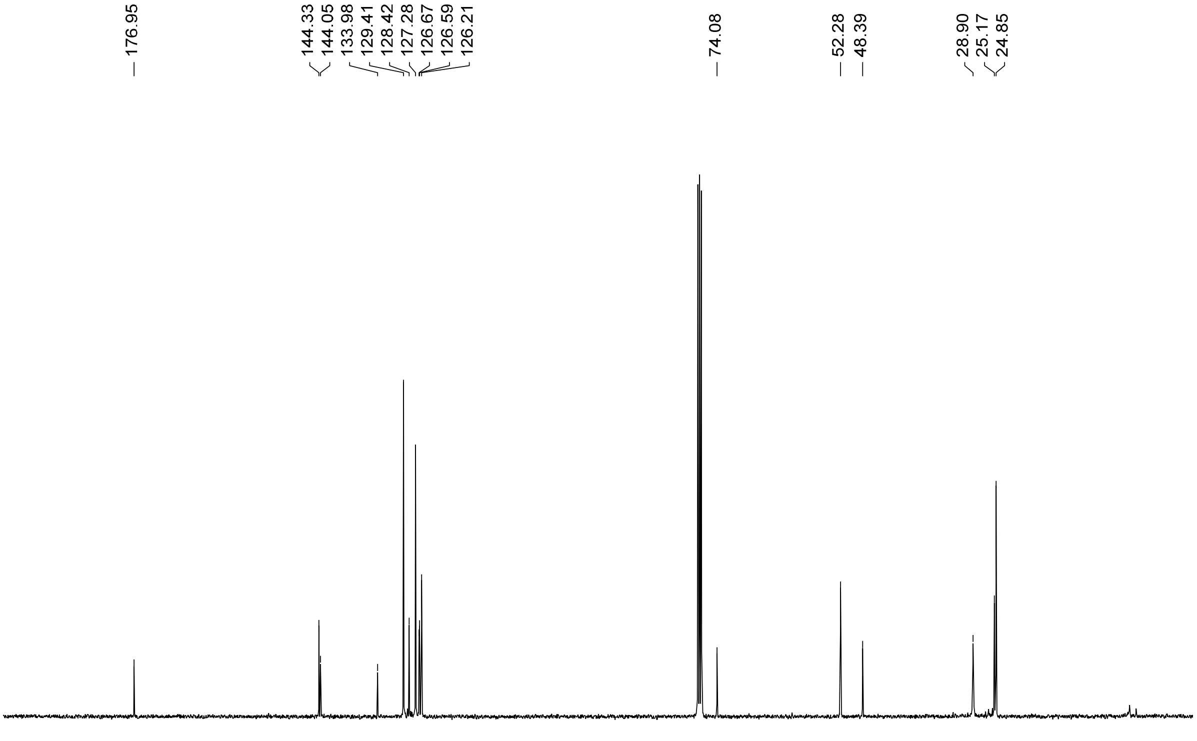

Supplementary Figure 32. ^{1}H and ^{13}C{^{1}H} NMR spectra of desired α-amino acid dicyclohexylamine salt **4ad** in $CDCl_3$

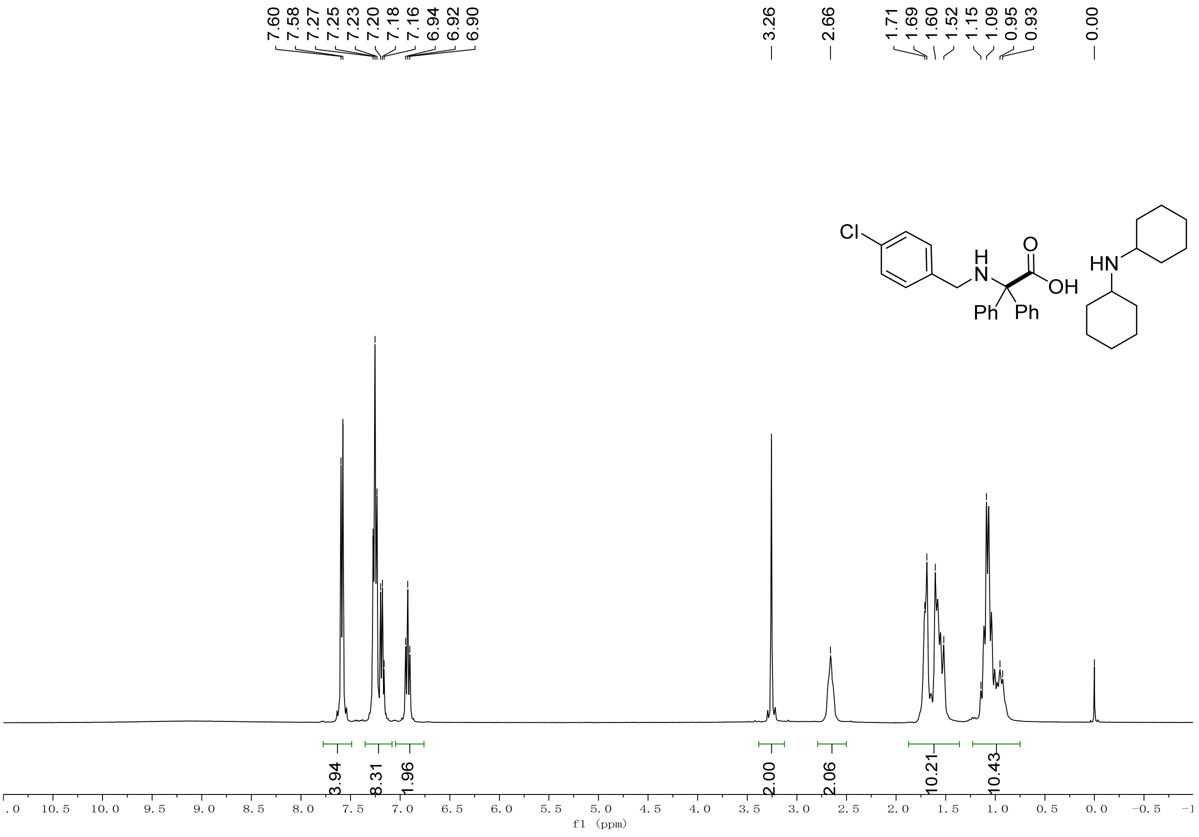

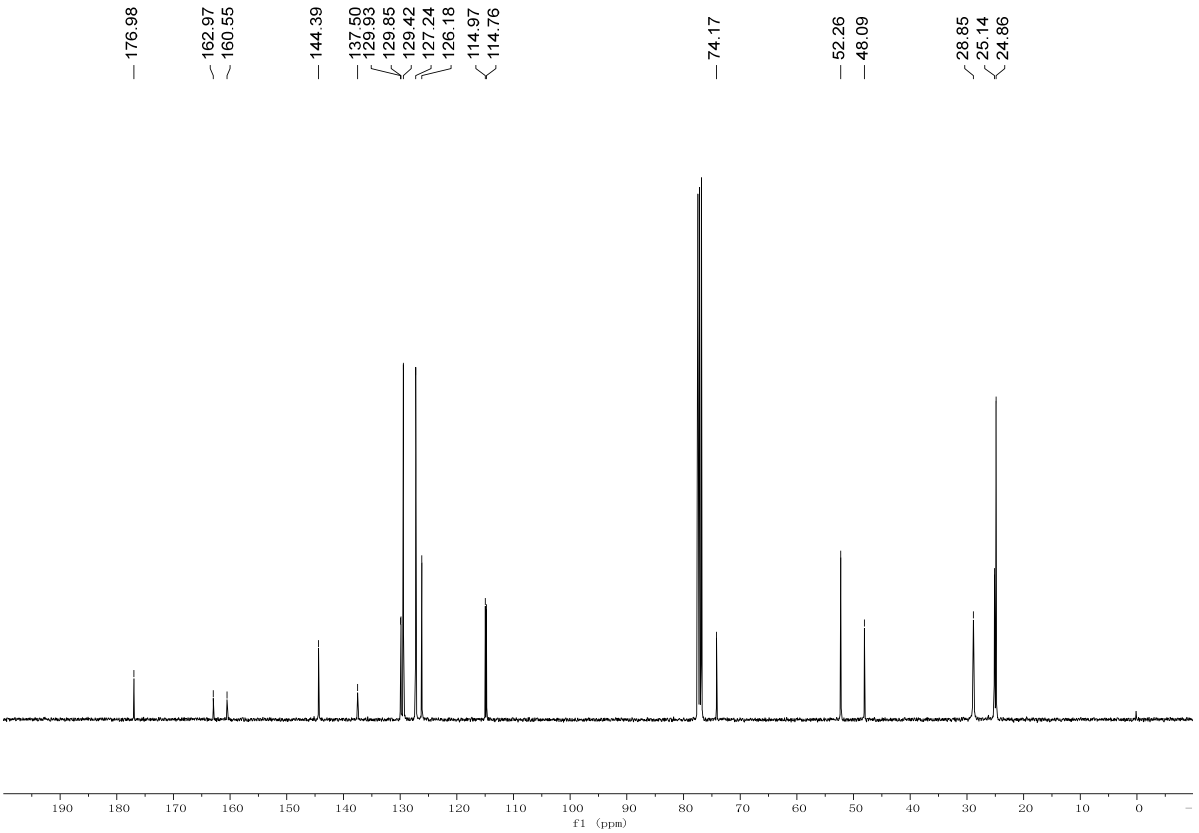

Supplementary Figure 33. ^{1}H and ^{13}C{^{1}H} NMR spectra of desired α-amino acid dicyclohexylamine salt **4ae** in $CDCl_3$

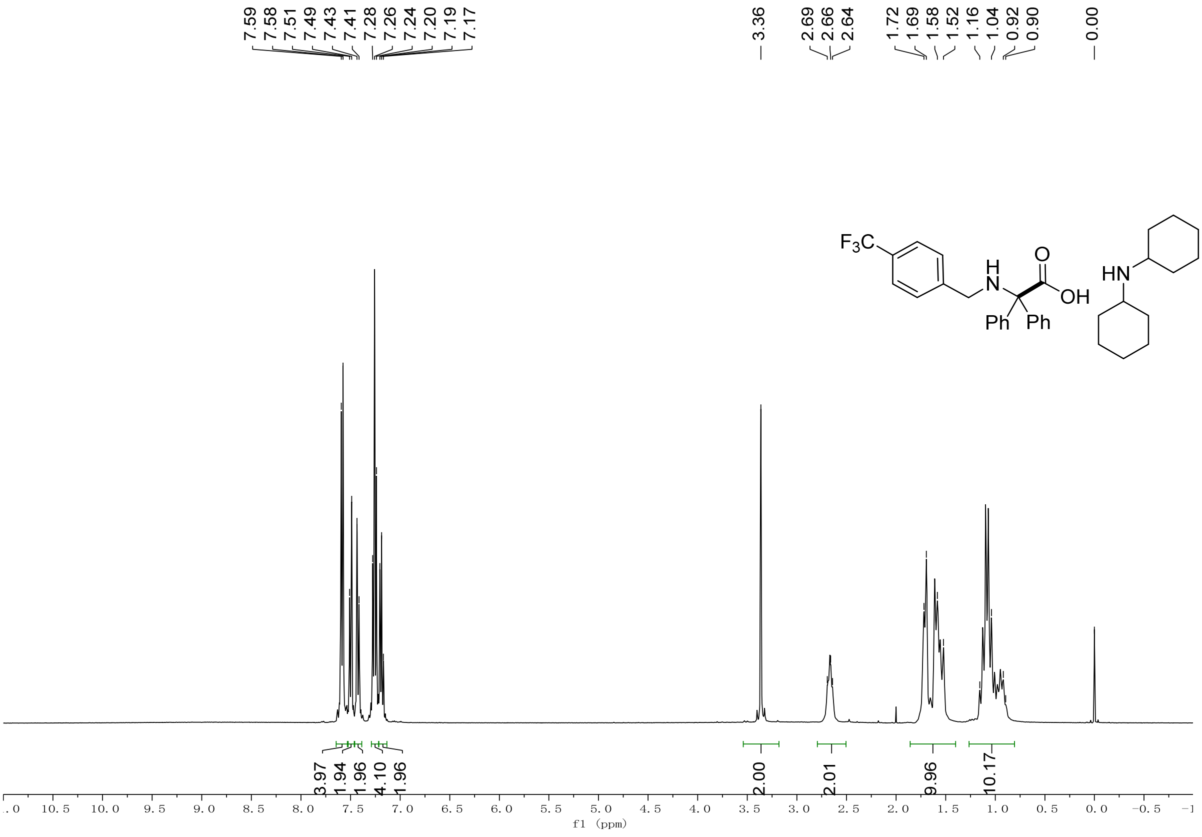

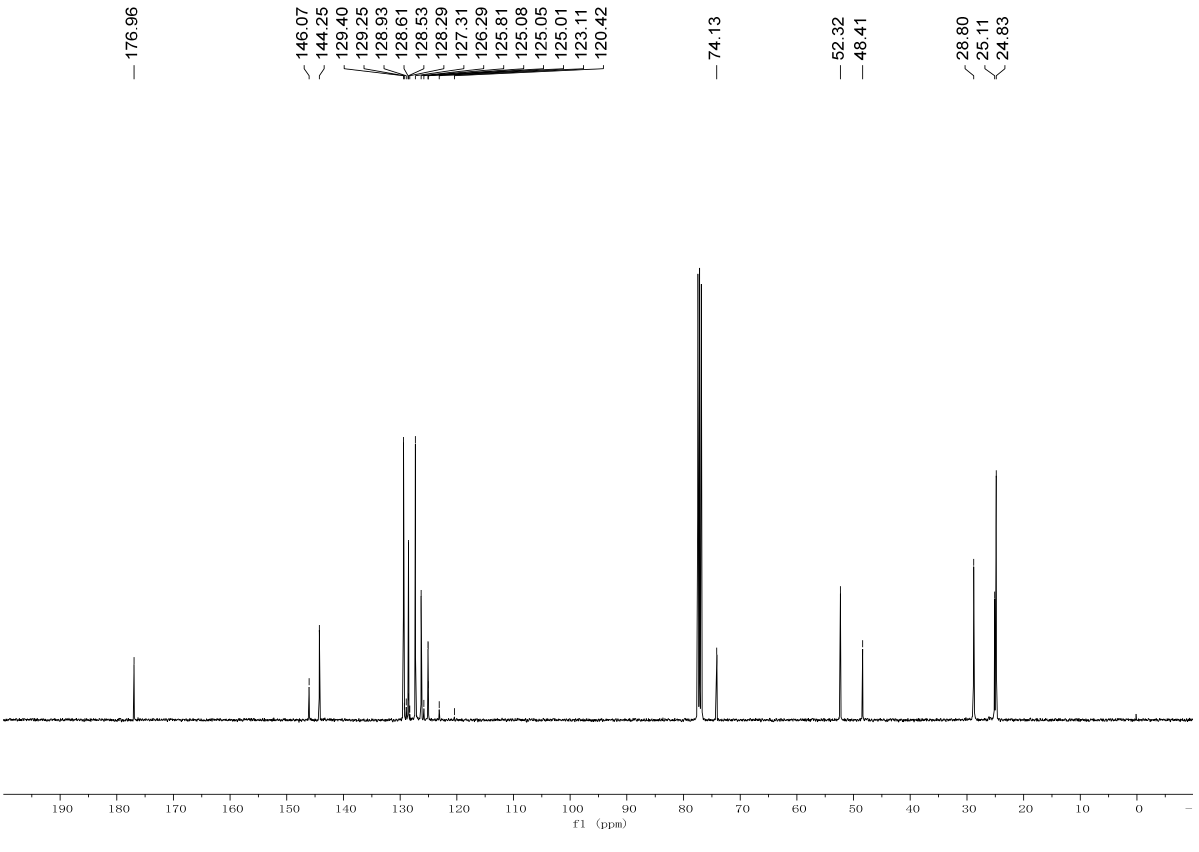

Supplementary Figure 34. ^{1}H and ^{13}C{^{1}H} NMR spectra of desired α-amino acid dicyclohexylamine salt **4af** in $CDCl_3$

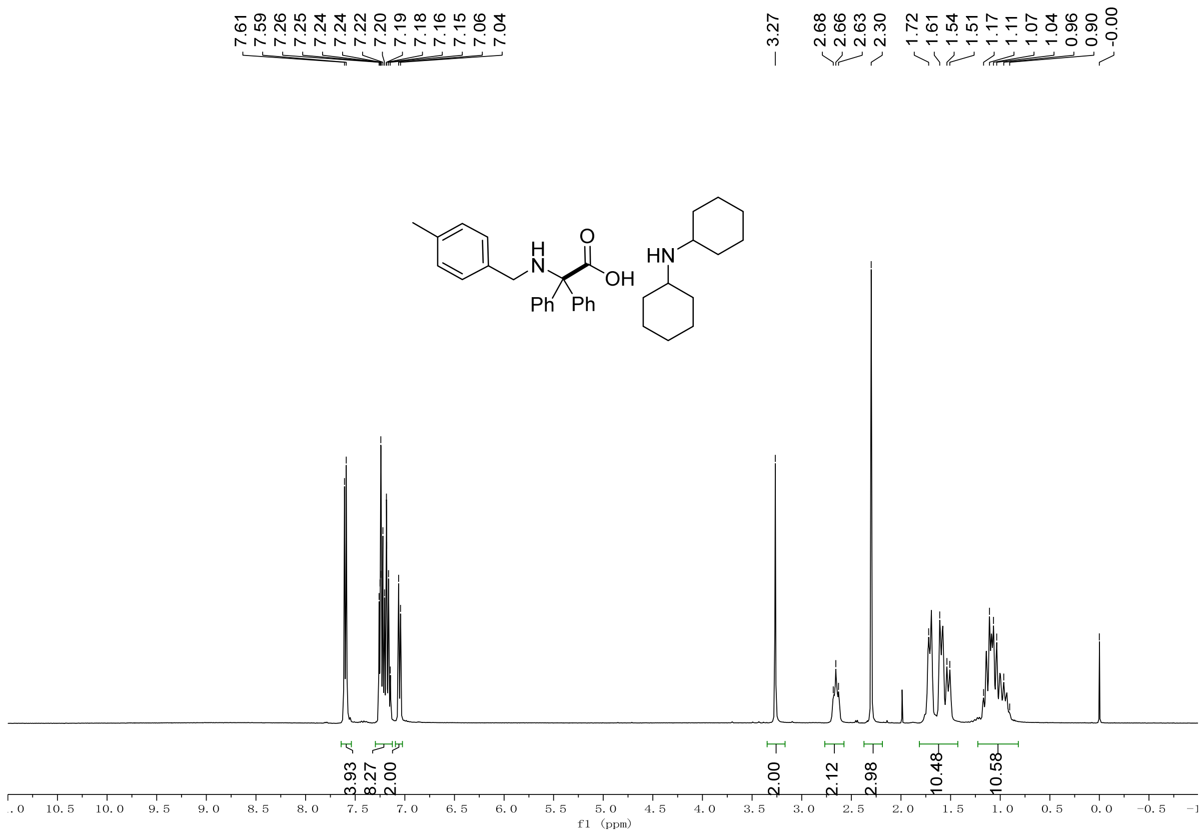

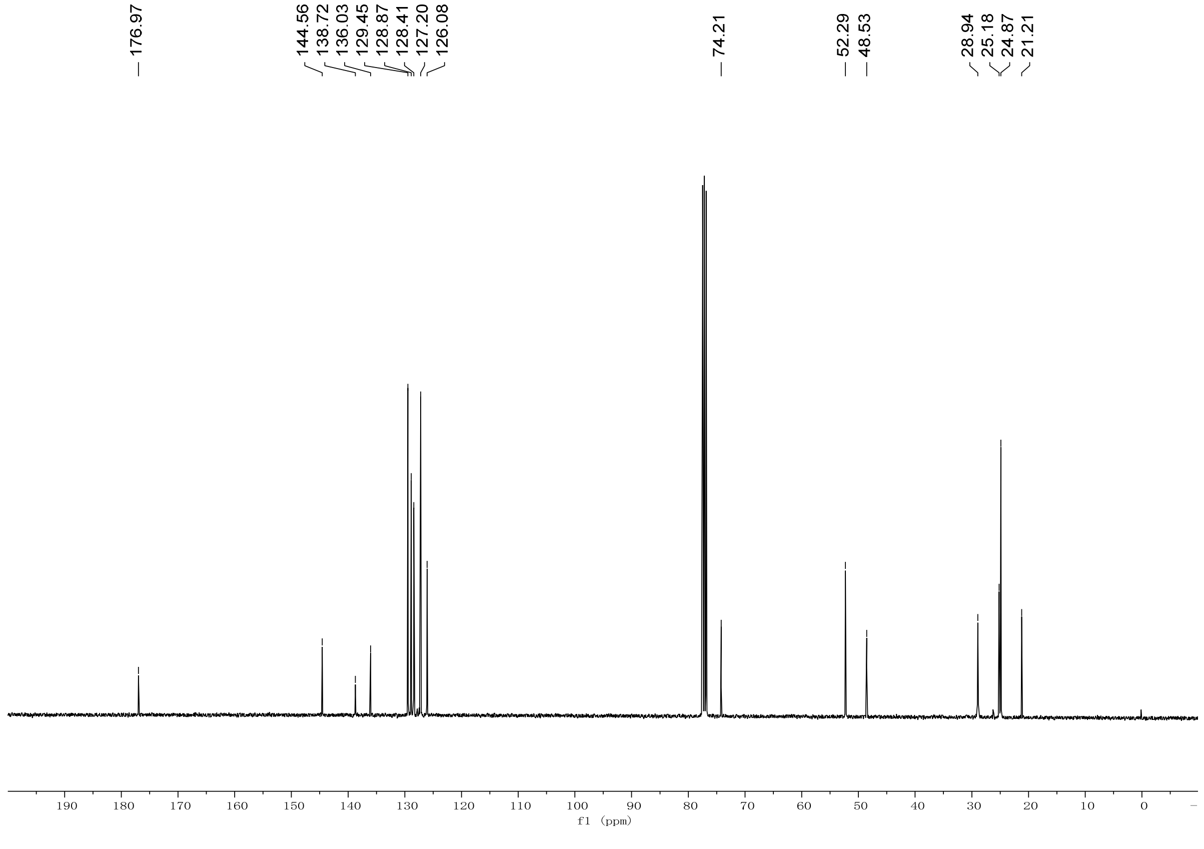

Supplementary Figure 35. ^{1}H and $^{13}C\{^{1}H\}$ NMR spectra of desired α-amino acid dicyclohexylamine salt **4ag** in $CDCl_3$

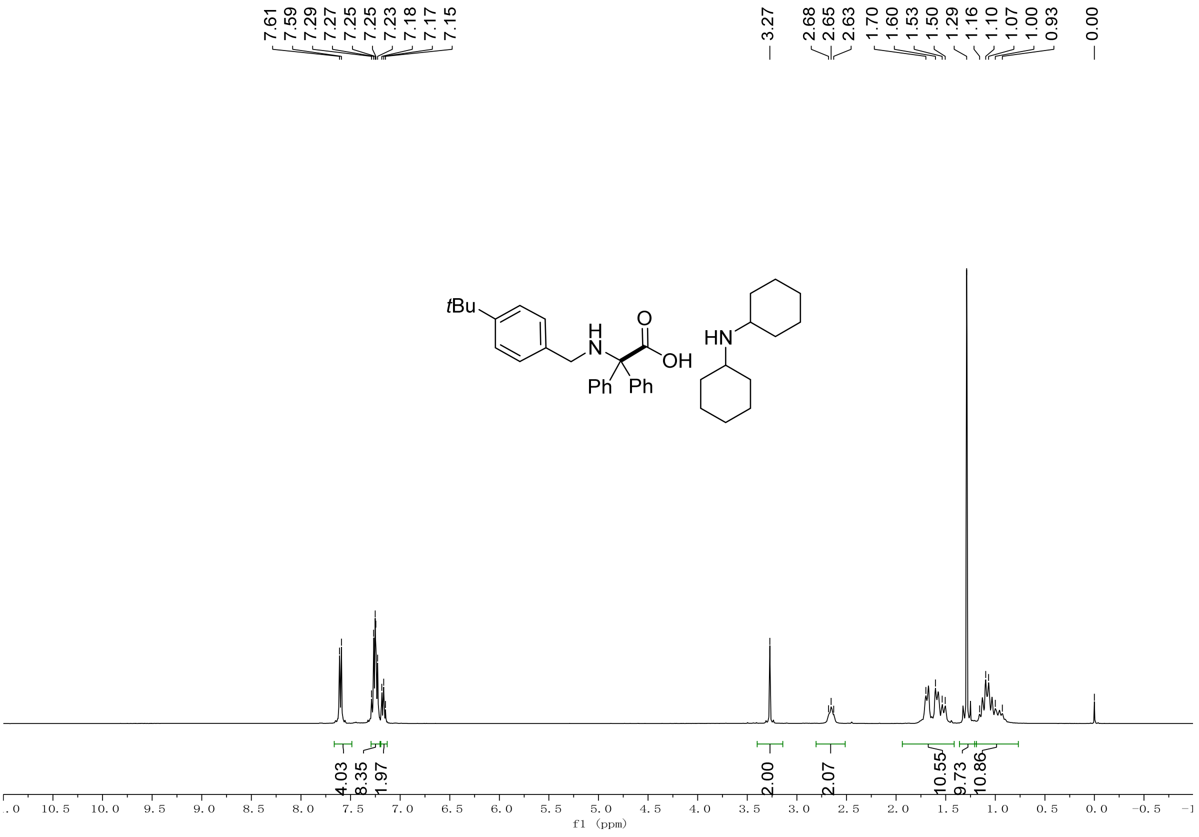

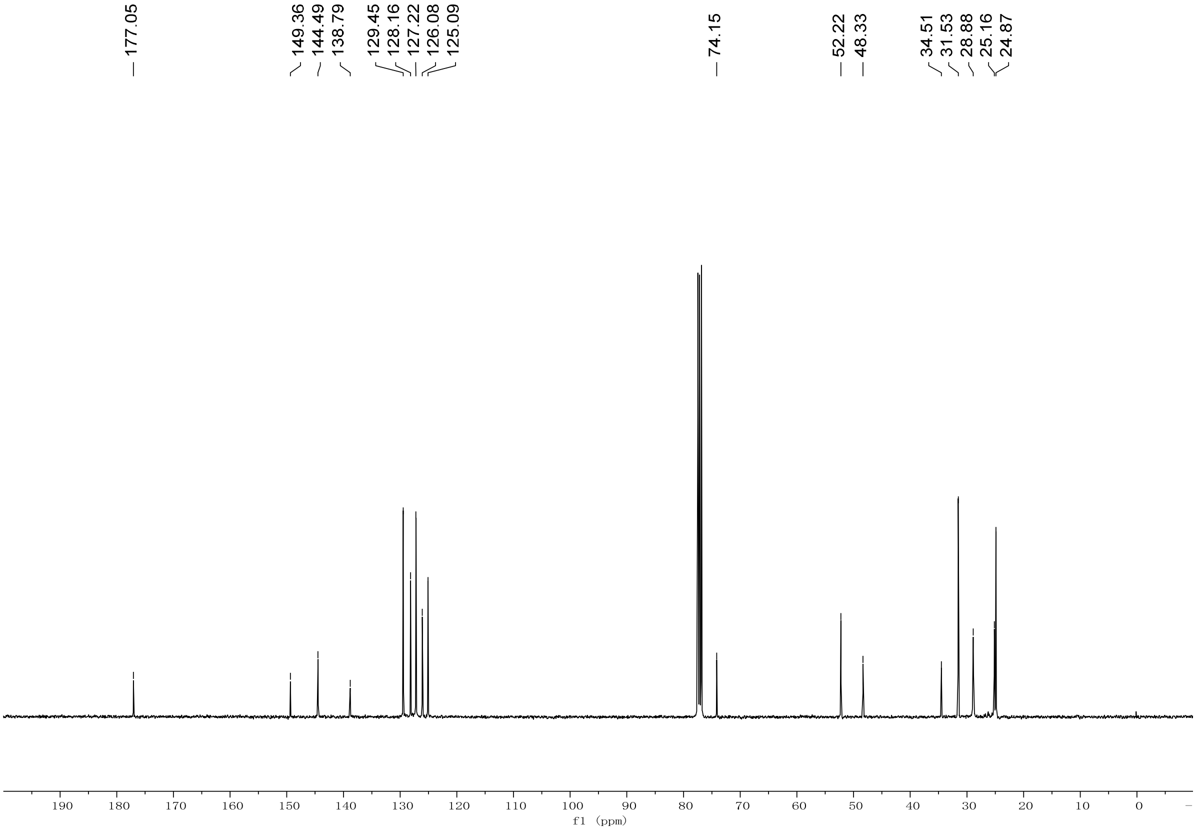

Supplementary Figure 36. ^{1}H and $^{13}C\{^{1}H\}$ NMR spectra of desired α-amino acid dicyclohexylamine salt **4ah** in $CDCl_3$

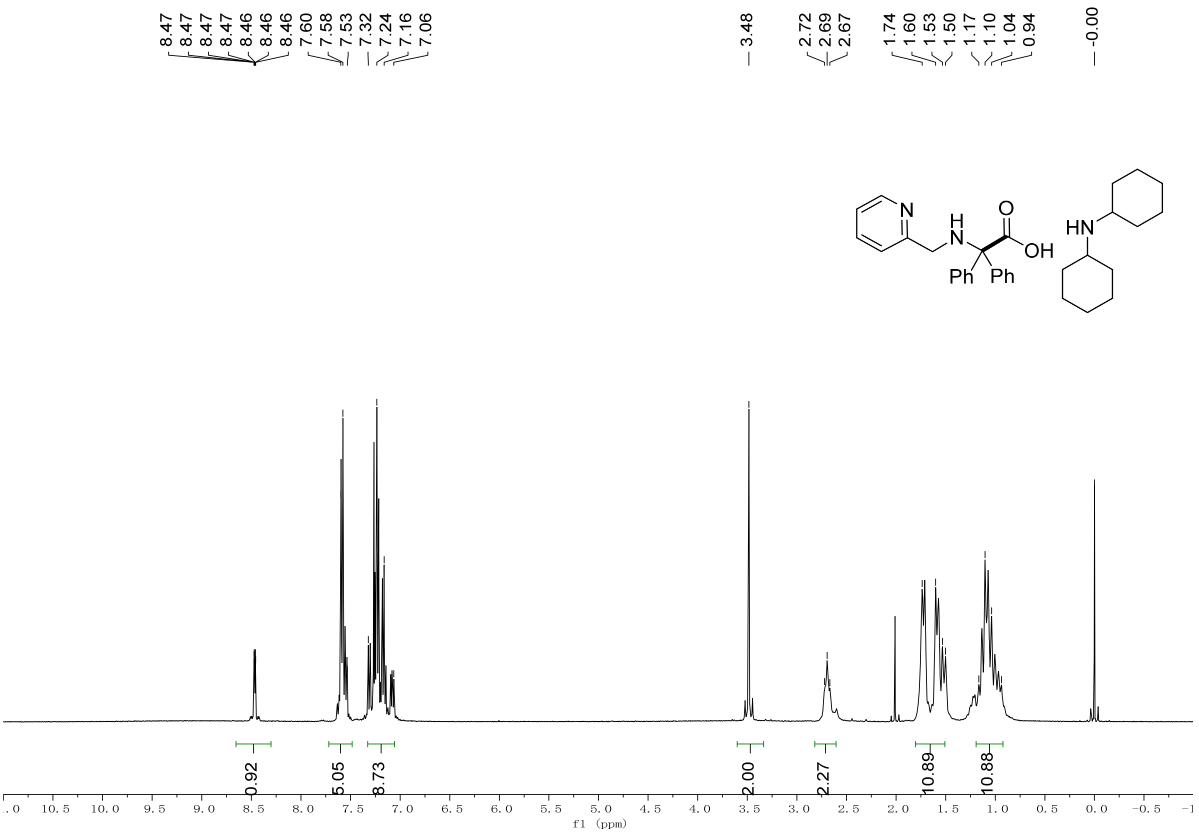

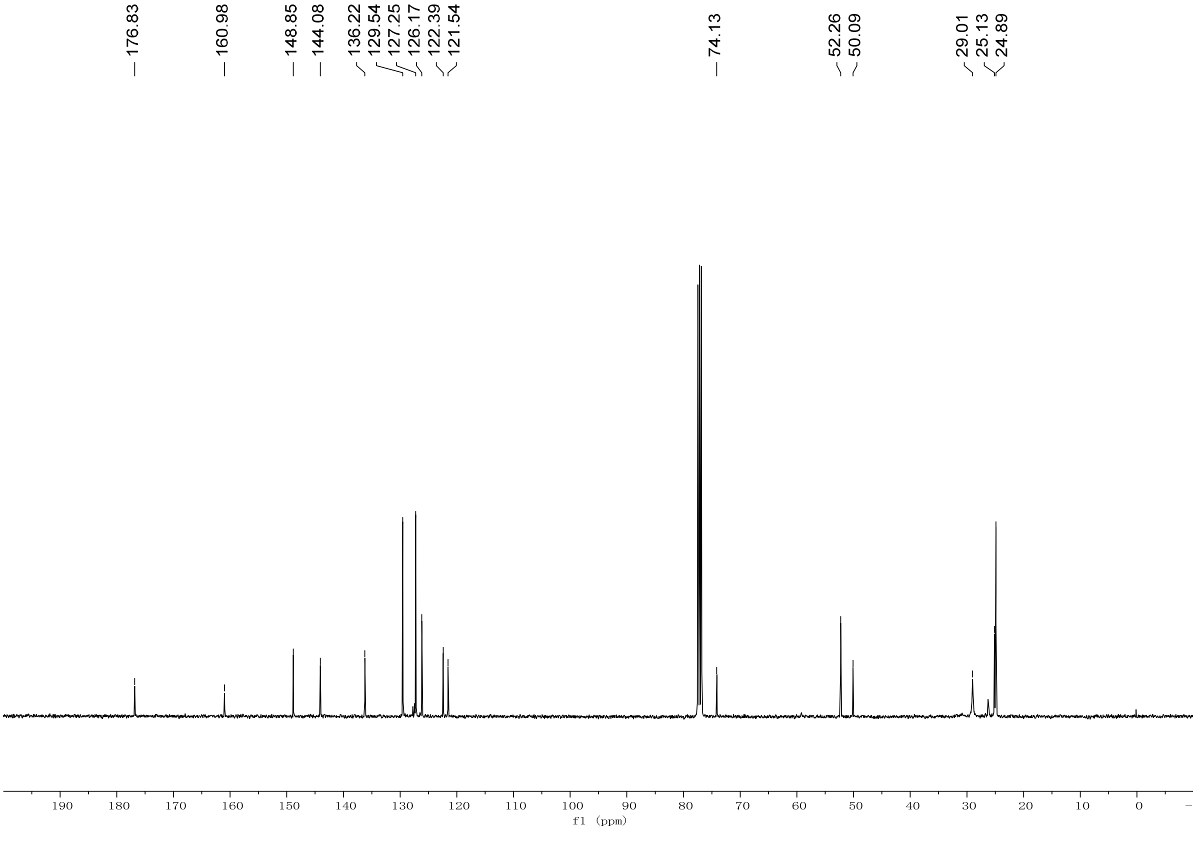

Supplementary Figure 37. ^{1}H and $^{13}C\{^{1}H\}$ NMR spectra of desired α-amino acid dicyclohexylamine salt **4ai** in $CDCl_3$

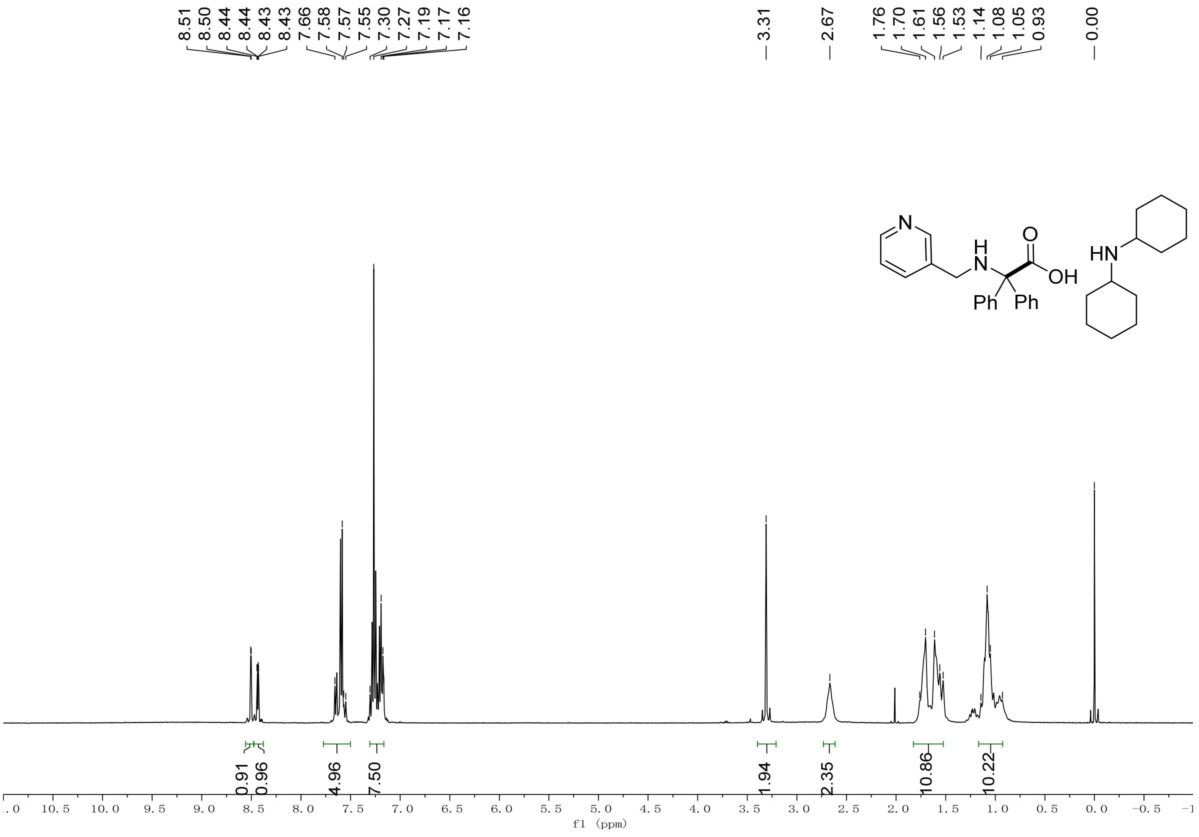

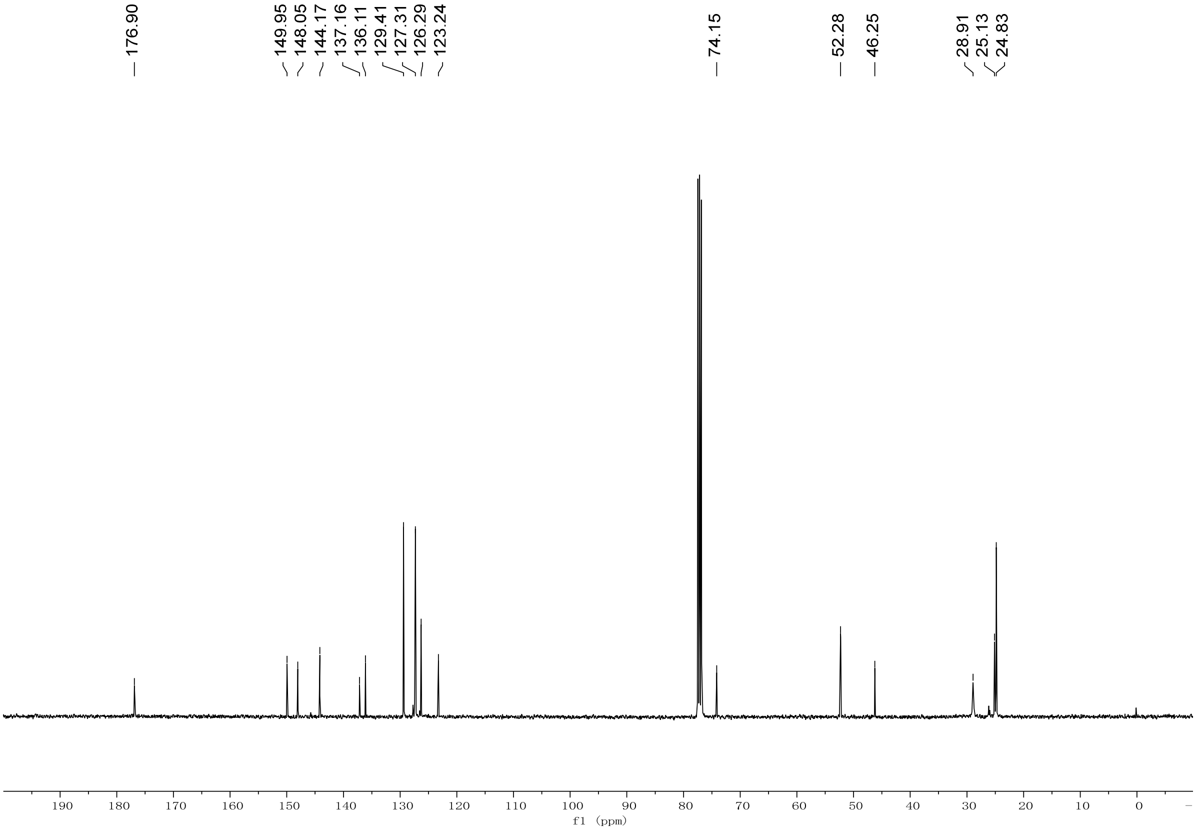

Supplementary Figure 38. ^{1}H and ^{13}C{^{1}H} NMR spectra of desired α-amino acid dicyclohexylamine salt **4aj** in $CDCl_3$

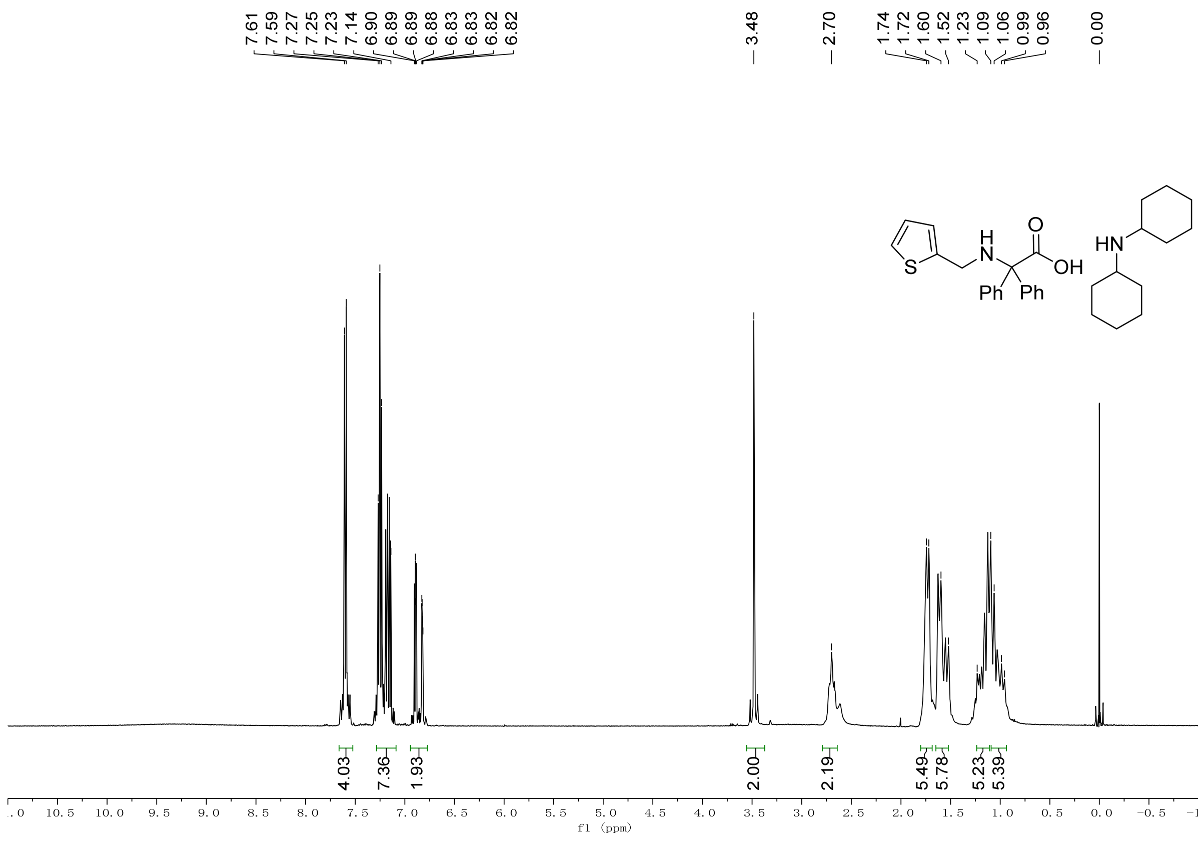

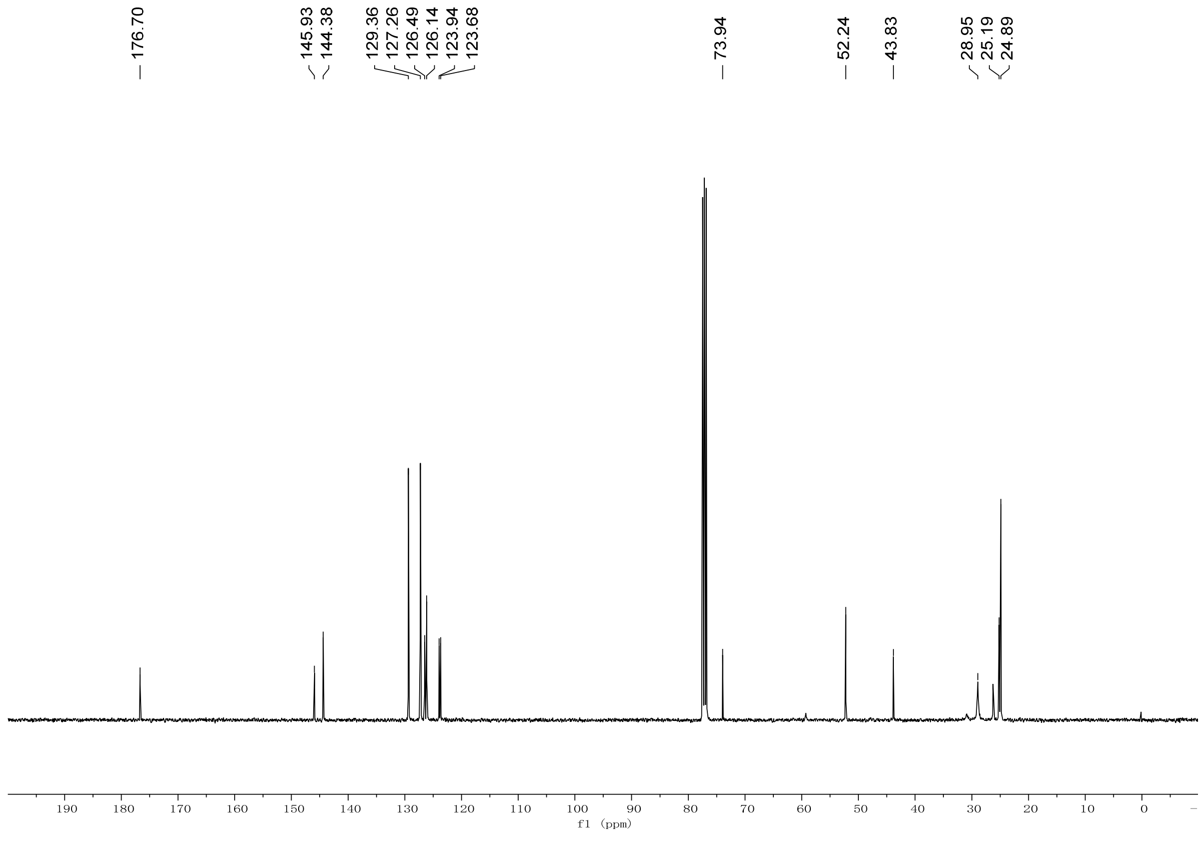

Supplementary Figure 39. ^{1}H and ^{13}C{^{1}H} NMR spectra of desired α-amino acid dicyclohexylamine salt **4ak** in $CDCl_3$

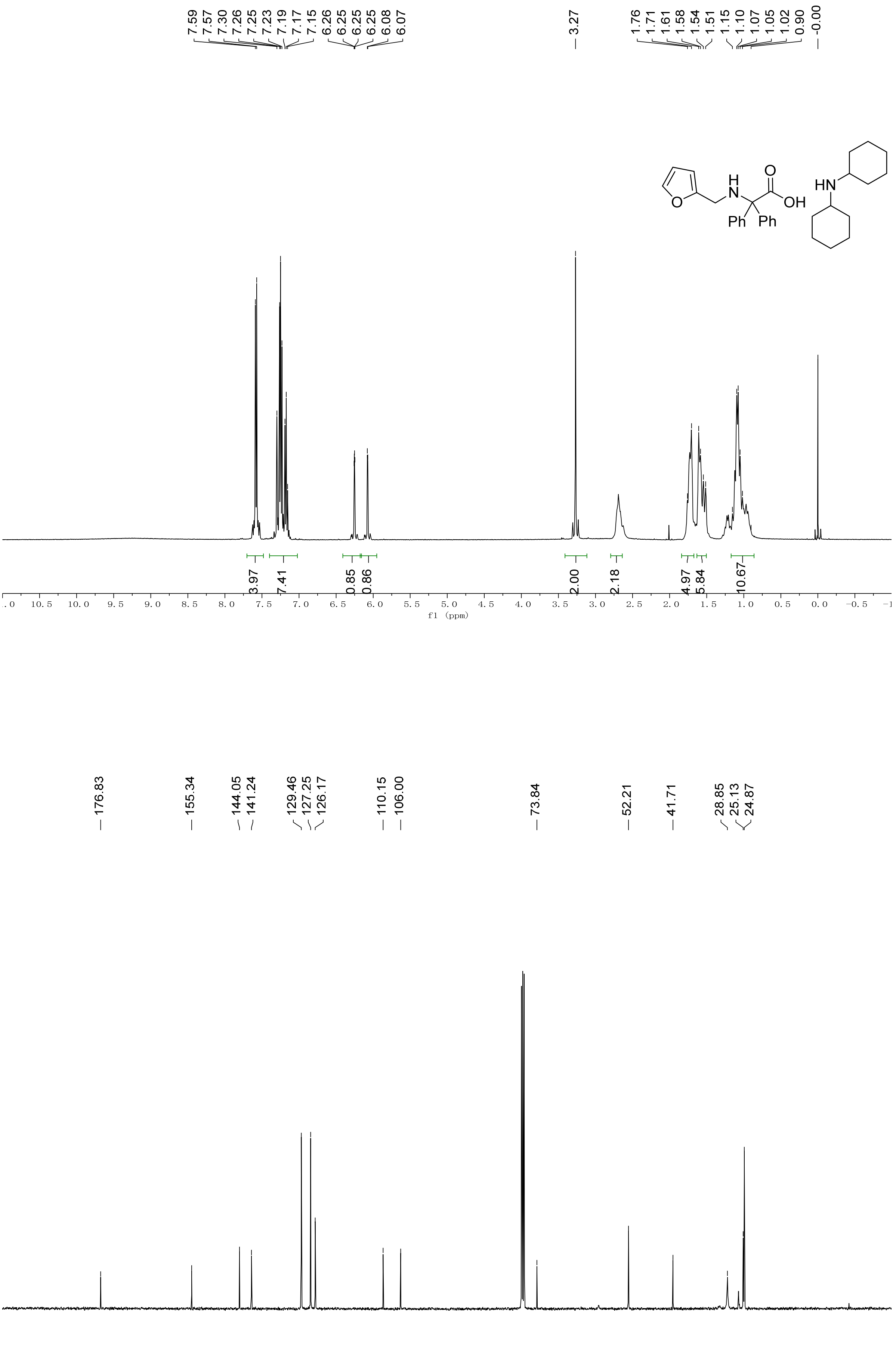

Supplementary Figure 40. ^{1}H and $^{13}C\{^{1}H\}$ NMR spectra of desired α-amino acid dicyclohexylamine salt **4al** in $CDCl_3$

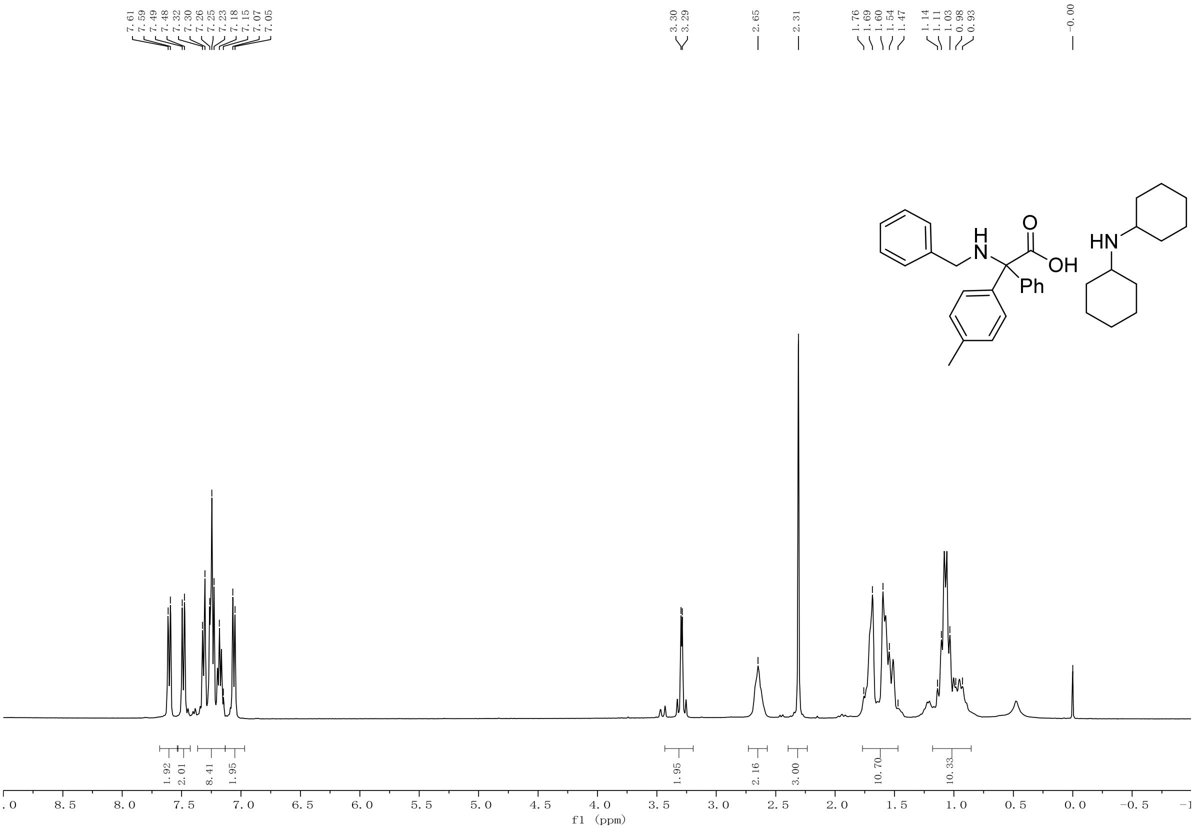

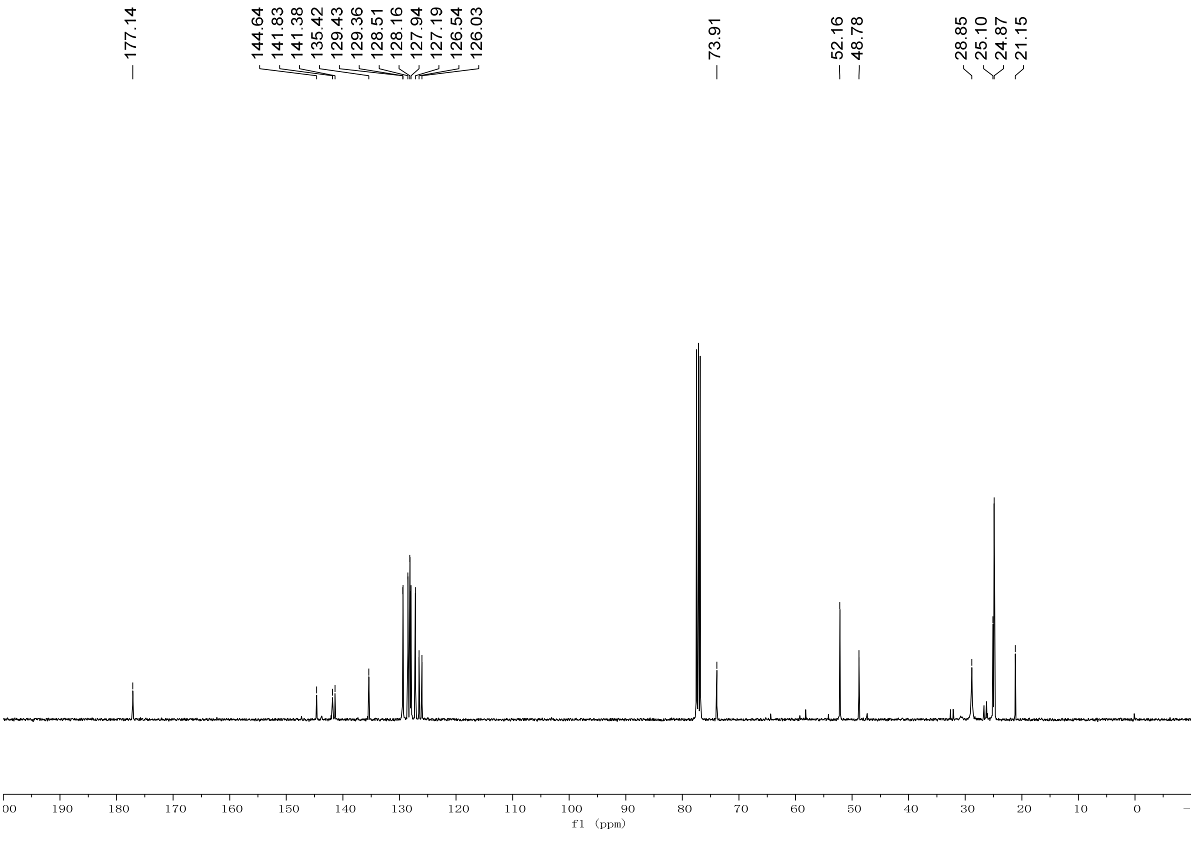

Supplementary Figure 41. ^{1}H and ^{13}C{^{1}H} NMR spectra of **5a** in $CDCl_3$

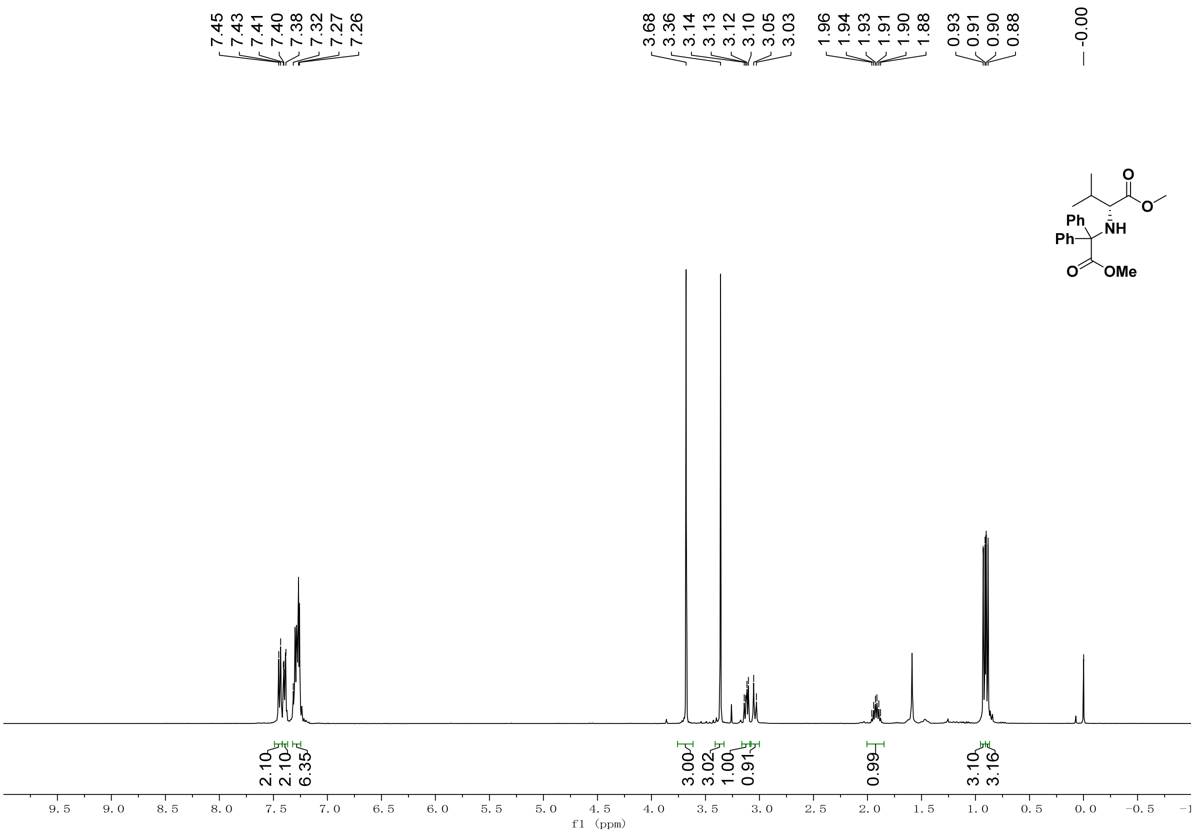

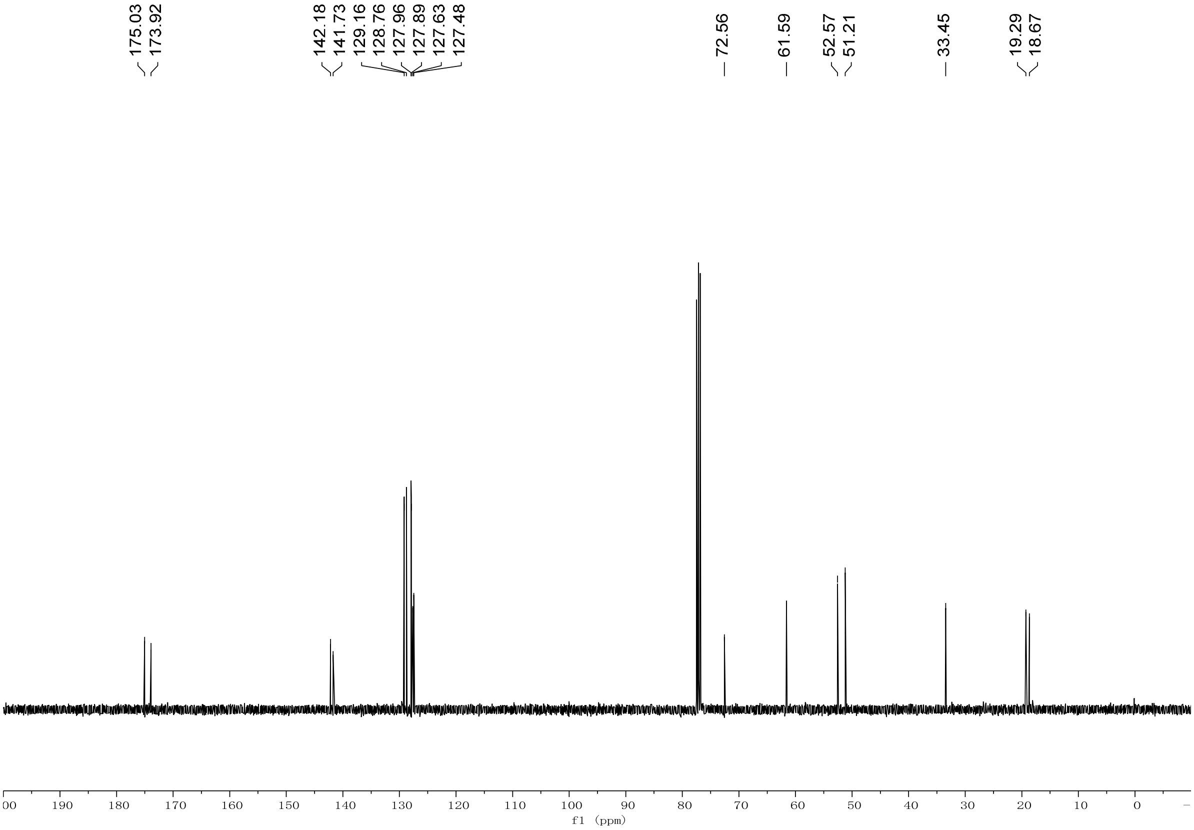

Supplementary Figure 42. ^{1}H and ^{13}C{^{1}H} NMR spectra of **5b** in $CDCl_3$

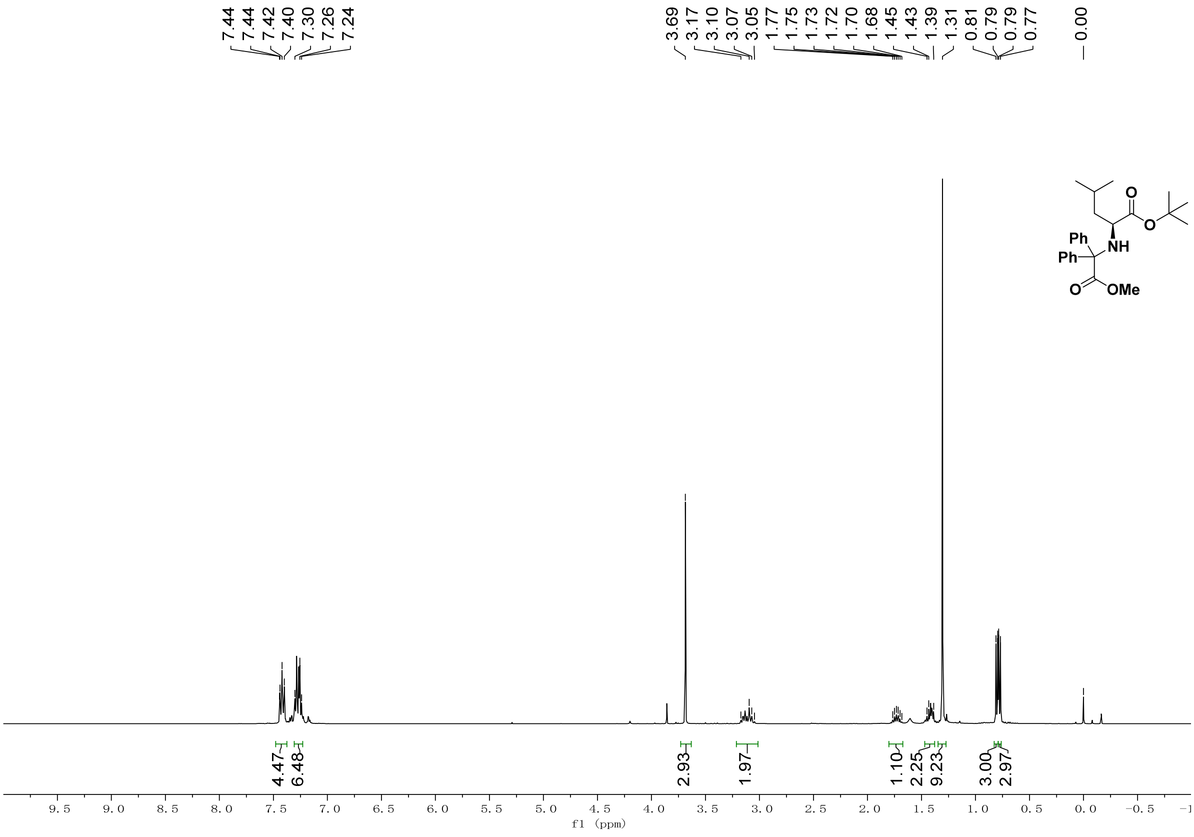

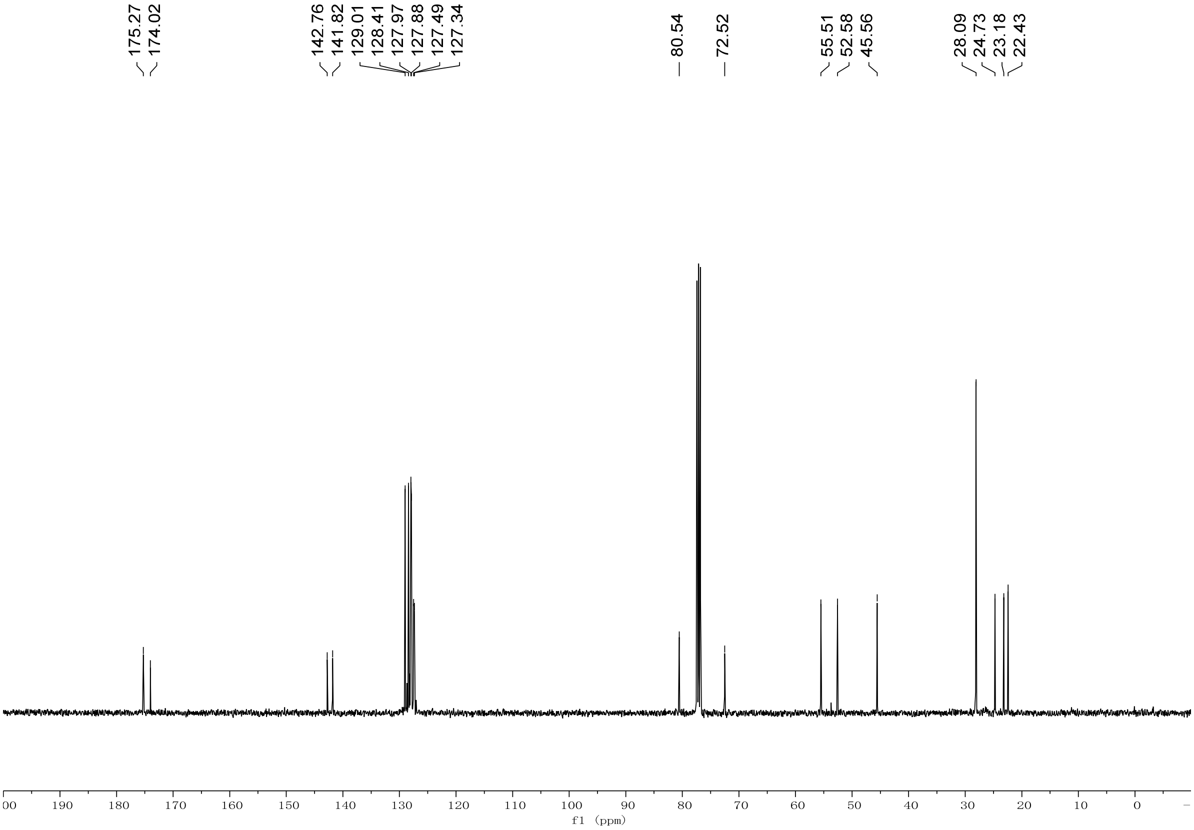

Supplementary Figure 43. ^{1}H and ^{13}C{^{1}H} NMR spectra of **5c** in $CDCl_3$

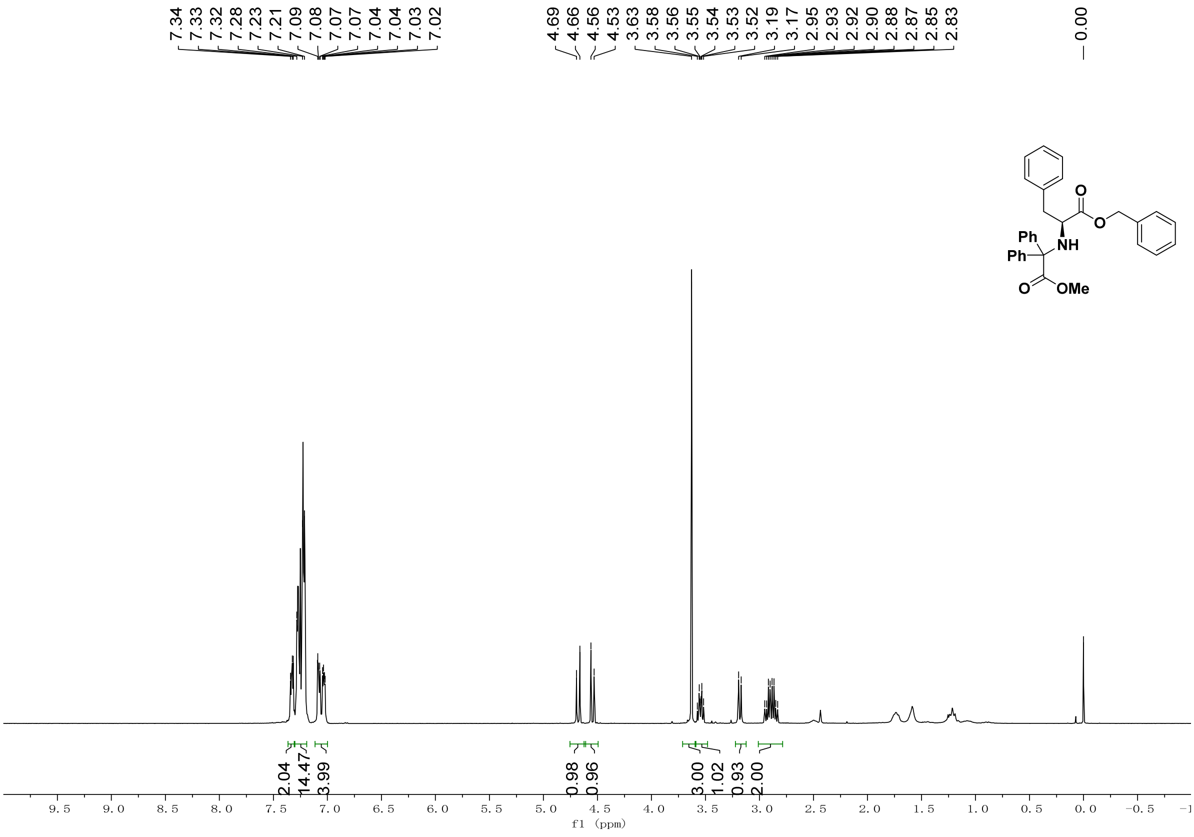

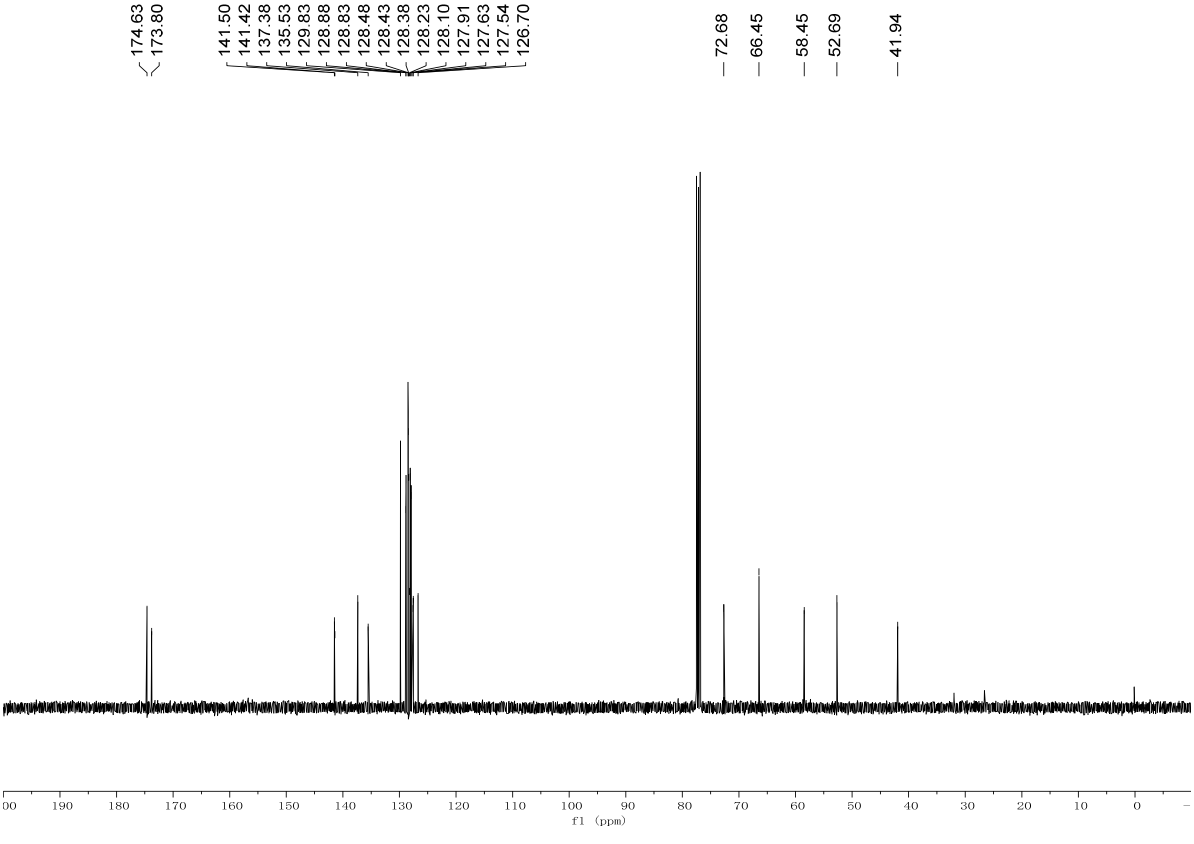

Supplementary Figure 44. ^{1}H and ^{13}C{^{1}H} NMR spectra of **5d** in $CDCl_3$

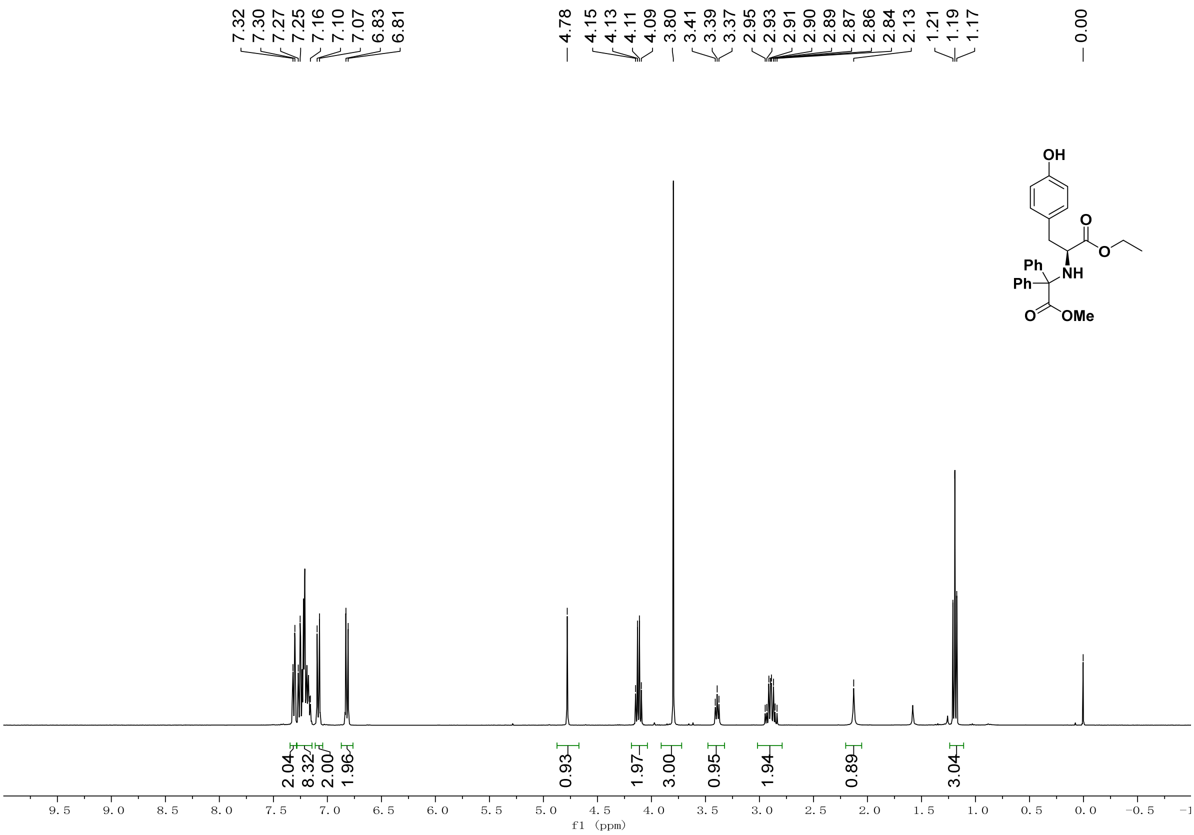

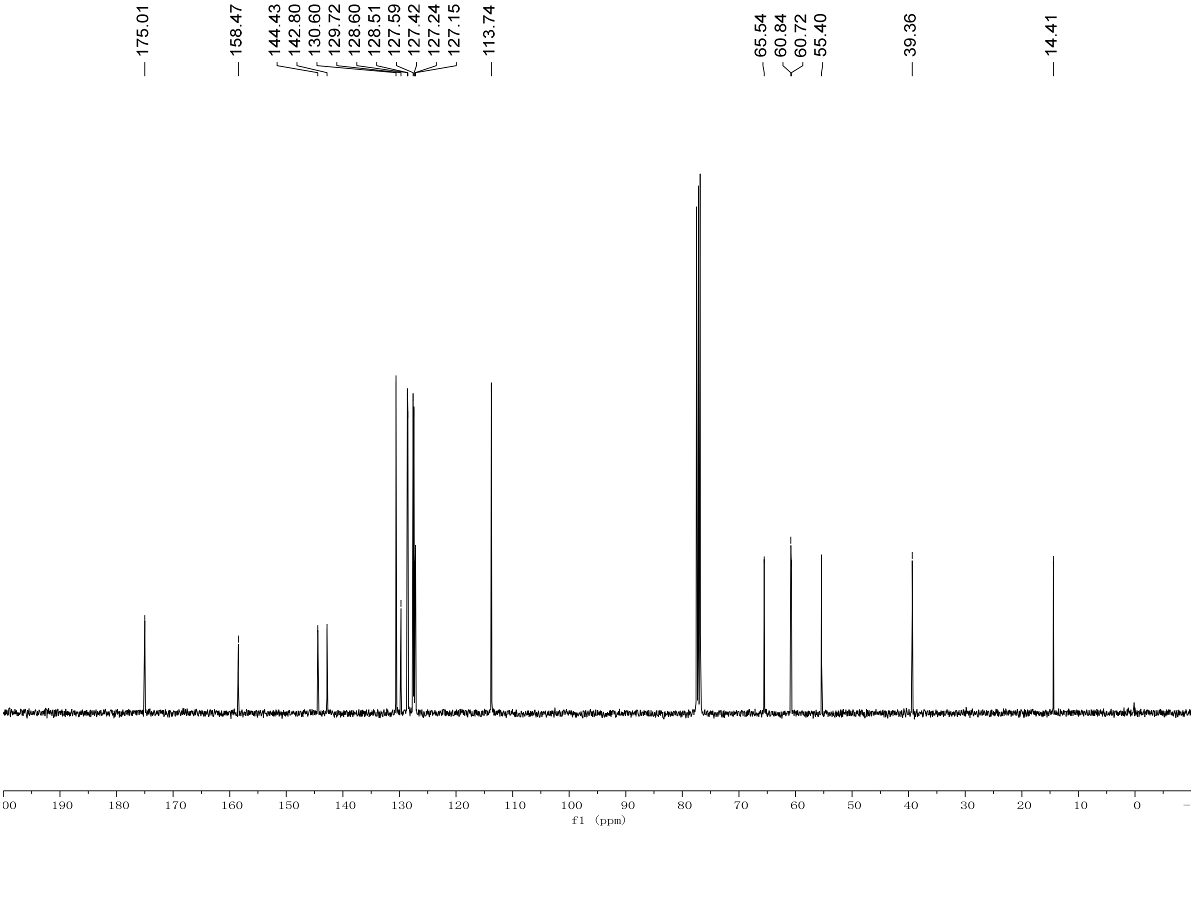

Supplementary Figure 45. ^{1}H and $^{13}C\{^{1}H\}$ NMR spectra of **6aa** in D_2O

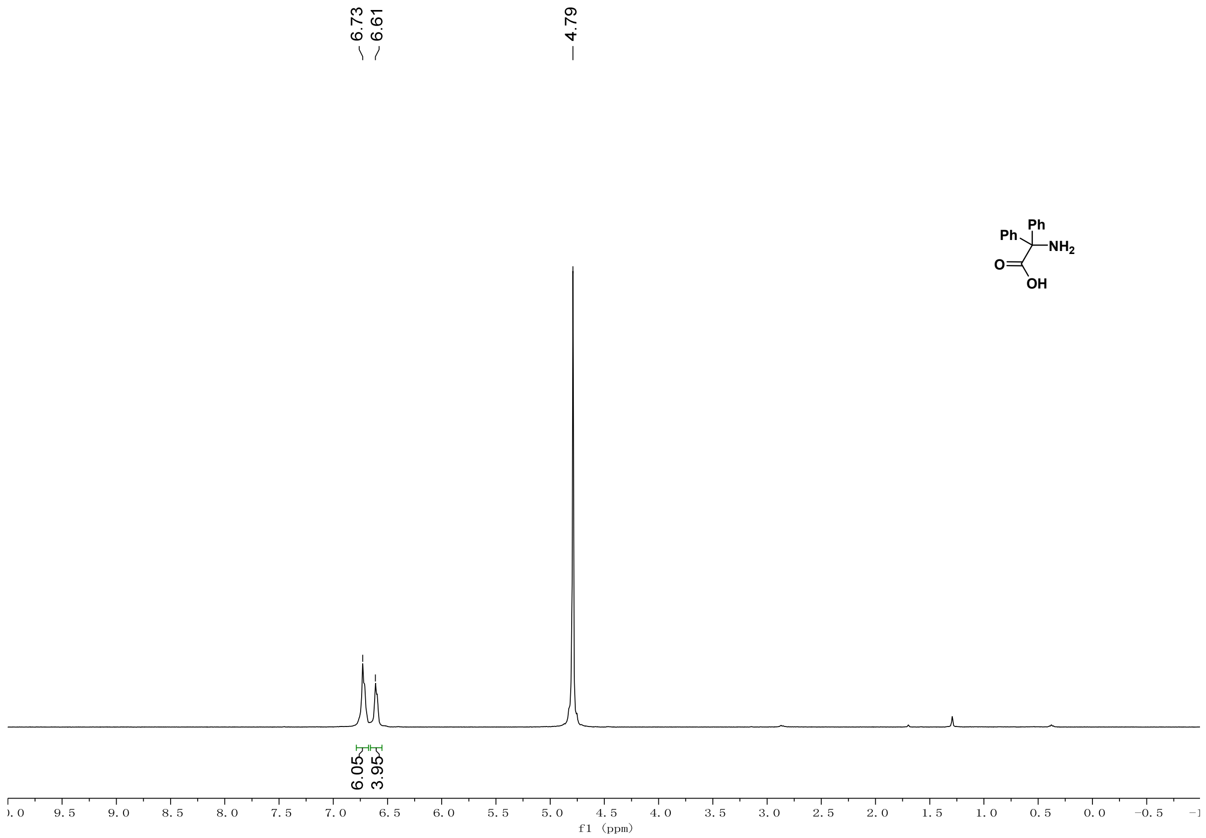

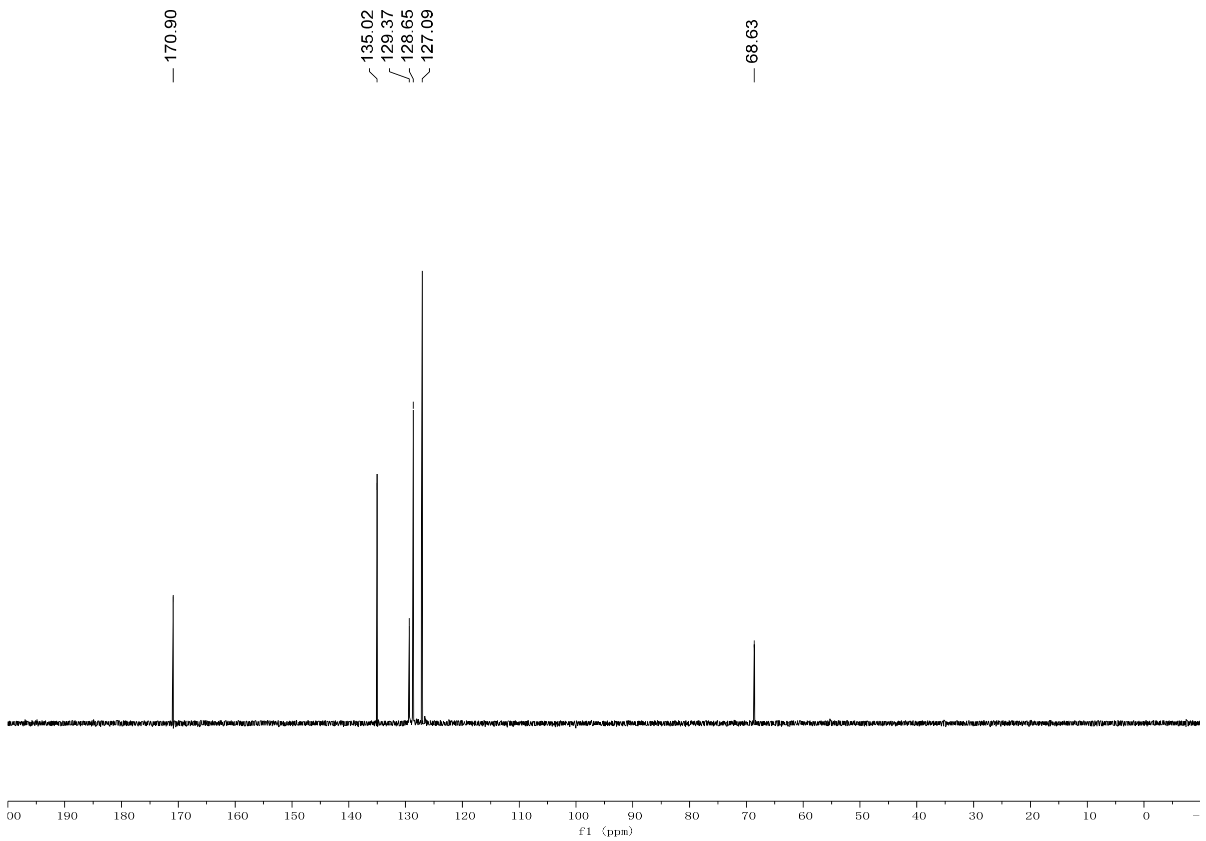

Supplementary Figure 46. ^{1}H and $^{13}C\{^{1}H\}$ NMR spectra of dipeptide in $CDCl_3$

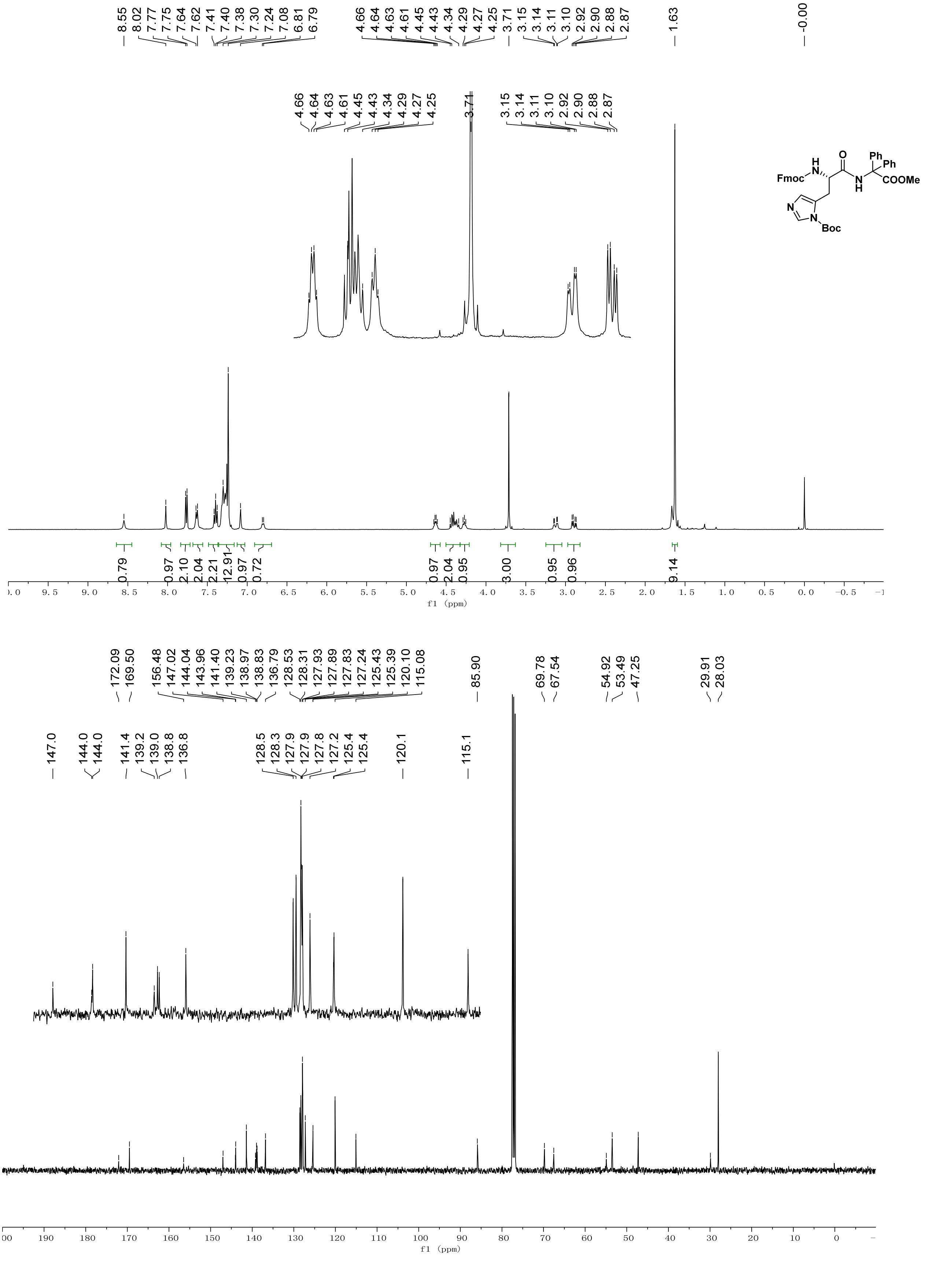

Supplementary Figure 47. HPLC Chromatography of compounds 5a.

Methyl (2-methoxy-2-oxo-1,1-diphenylethyl)-D-valinate (5a): Daicel Chiralpak OD-H column (0.5% isopropanol in hexanes, 0.3 mL/min, 254 nm, t_r (D/R, major) = 28.1 min, t_r (L/S) = 29.7 min).

Racemate:

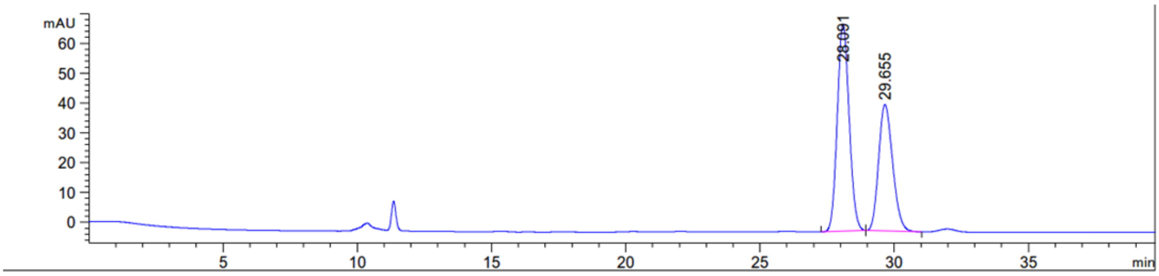

Peak #	RetTime [min]	Type	Width [min]	Area [mAU*s]	Height [mAU]	Area %
1	28.091	BB	0.4761	2133.63599	69.50897	58.0772
2	29.655	BB	0.5655	1540.15918	42.42605	41.9228

5a:

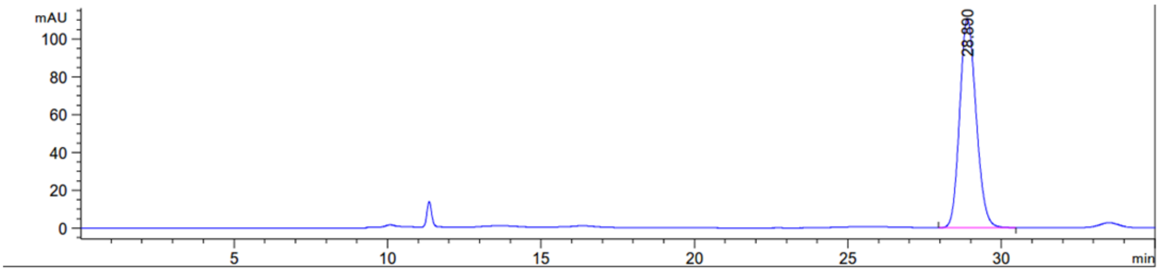

Peak #	RetTime [min]	Type	Width [min]	Area [mAU*s]	Height [mAU]	Area %
1	28.890	BB	0.5766	4102.59521	110.64766	100.0000

Supplementary Figure 48. HPLC Chromatography of compounds 5b.

***tert*-butyl (2-methoxy-2-oxo-1,1-diphenylethyl)-L-leucinate (5b)**: Daicel Chiralpak OD-H column (0.5% isopropanol in hexanes, 0.3 mL/min, 254 nm, t_r (L/S, major) = 17.2 min, t_r (D/R) = 17.9 min)..

Racemate:

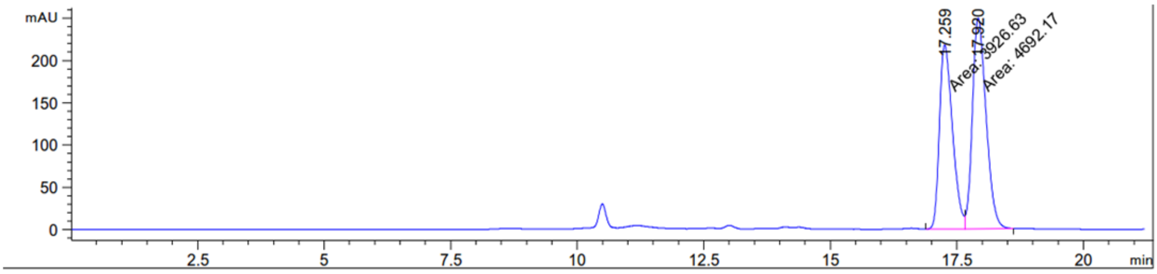

Peak #	RetTime [min]	Type	Width [min]	Area [mAU*s]	Height [mAU]	Area %
1	17.259	MF	0.2999	3926.63184	218.19261	45.5589
2	17.920	FM	0.3135	4692.17285	249.43129	54.4411

5b:

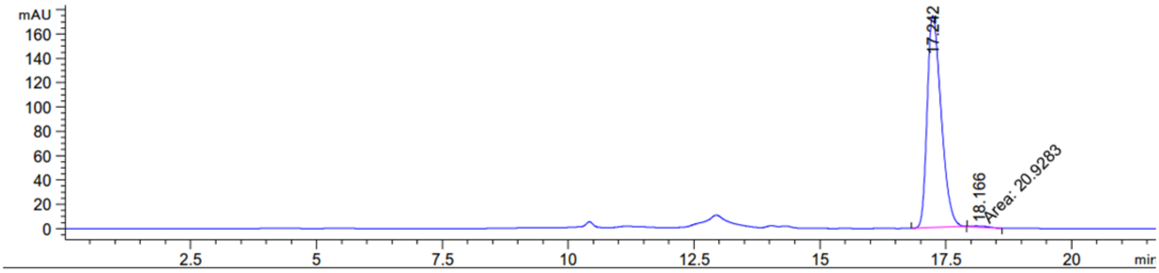

Peak #	RetTime [min]	Type	Width [min]	Area [mAU*s]	Height [mAU]	Area %
1	17.242	BB	0.2971	3367.70215	174.53494	99.3824
2	18.166	MM	0.3781	20.92834	9.22611e-1	0.6176

Supplementary Figure 49. HPLC Chromatography of compounds 5c.

Benzyl (2-methoxy-2-oxo-1,1-diphenylethyl)-D-phenylalaninate (**5c**): Daicel Chiralpak OD-H column (5% isopropanol in hexanes, 0.5 mL/min, 254 nm, t_r (D/R, major) = 19.6 min, t_r (L/S) = 29.8 min).

Racemate:

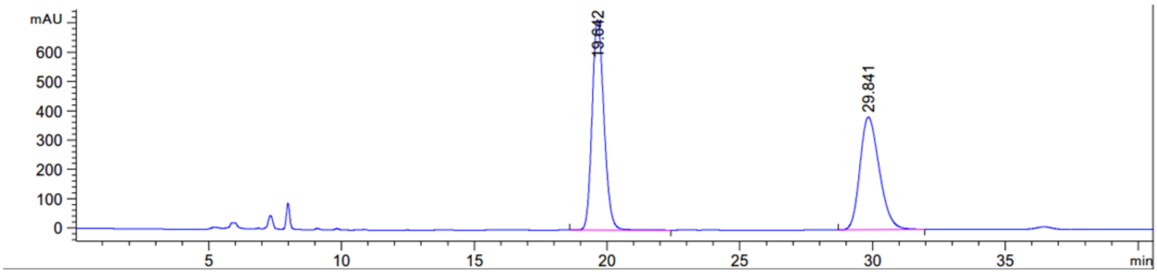

Peak #	RetTime [min]	Type	Width [min]	Area [mAU*s]	Height [mAU]	Area %
1	19.642	BB	0.4817	2.22702e4	718.41754	53.2946
2	29.841	BB	0.7834	1.95168e4	385.03534	46.7054

5c:

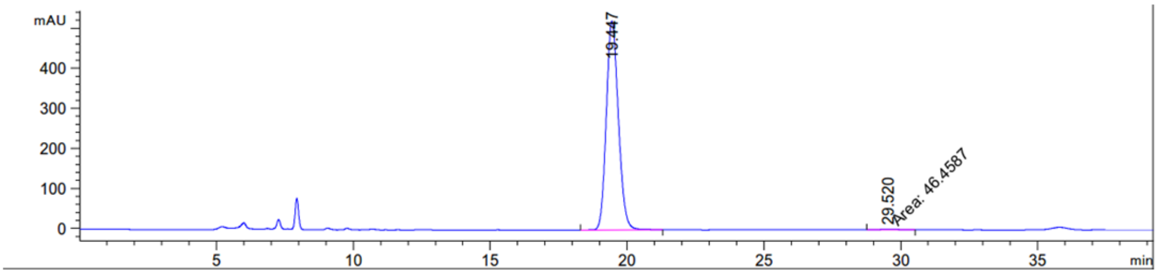

Peak #	RetTime [min]	Type	Width [min]	Area [mAU*s]	Height [mAU]	Area %
1	19.447	BB	0.4760	1.60250e4	522.27307	99.7109
2	29.520	MM	0.9286	46.45866	8.33840e-1	0.2891

Supplementary Figure 50. HPLC Chromatography of compounds 5d.

Ethyl (2-methoxy-2-oxo-1,1-diphenylethyl)-L-tyrosinate (**5d**): Daicel Chiralpak OD-H column (5% isopropanol in hexanes, 0.5 mL/min, 254 nm, t_r (L/S, major) = 13.2 min, t_r (D/R) = 13.9 min).

Racemate:

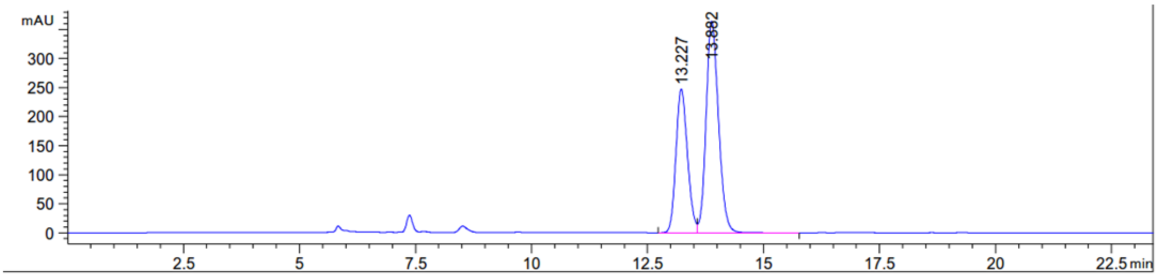

Peak #	RetTime [min]	Type	Width [min]	Area [mAU*s]	Height [mAU]	Area %
1	13.227	BV	0.2675	4274.91406	247.57033	38.5888
2	13.882	VB	0.2878	6803.19531	364.43265	61.4112

5d:

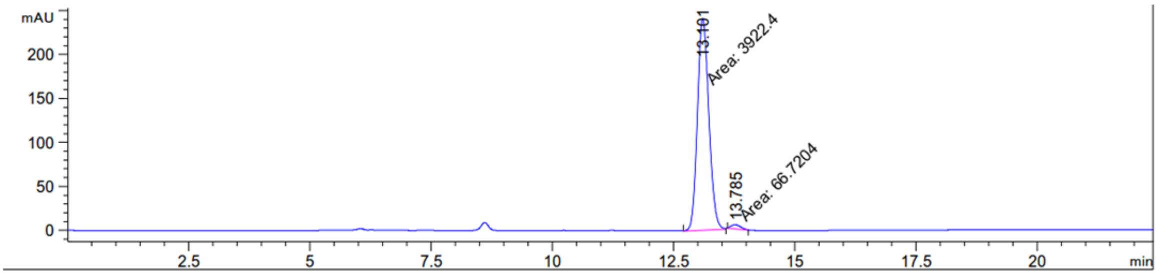

Peak #	RetTime [min]	Type	Width [min]	Area [mAU*s]	Height [mAU]	Area %
1	13.101	MM	0.2715	3922.40430	240.82463	98.3274
2	13.785	MM	0.2401	66.72041	4.63217	1.6726

Supplementary Figure 51. UV-Vis absorption analysis.

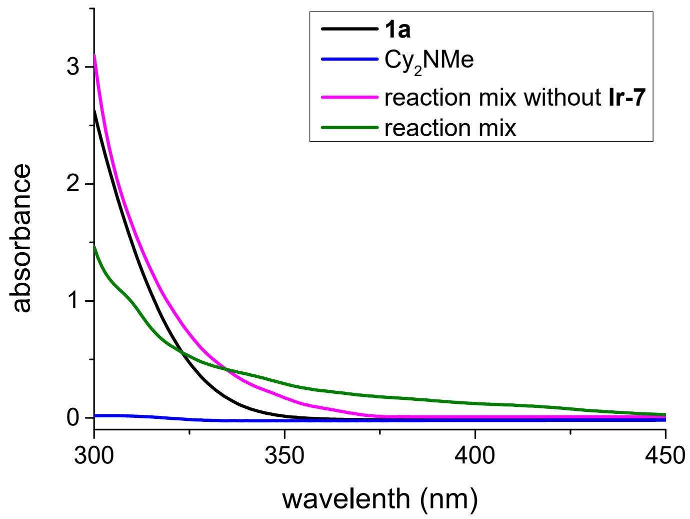

Supplementary Figure 52. Stern-Volmer fluorescence quenching analysis.

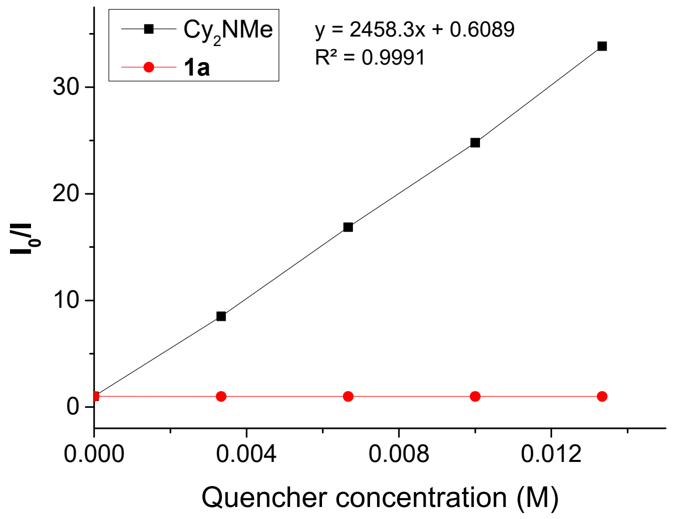

Supplementary Reference

1 Jirgensons, A., Kauss, V., Kalvinsh, I. & Gold, M. R. A Practical Synthesis of tert-Alkylamines via the Ritter Reaction with Chloroacetonitrile. *Synthesis* **2000**, 1709-1712, (2000).
